# Engineering Soluble Diketopyrrolopyrrole Chromophore Stacks from a Series of Pd(II)‐Based Ravels**

**DOI:** 10.1002/anie.202308288

**Published:** 2023-08-23

**Authors:** Irene Regeni, Rituparno Chowdhury, Kai Terlinden, Shinnosuke Horiuchi, Julian J. Holstein, Sascha Feldmann, Guido H. Clever

**Affiliations:** ^1^ Department of Chemistry and Chemical Biology TU Dortmund University Otto-Hahn-Strasse 6 44227 Dortmund Germany; ^2^ Current address: Leiden Institute of Chemistry Leiden University 2333CC Leiden The Netherlands; ^3^ Cavendish Laboratory University of Cambridge Cambridge CB30HE UK; ^4^ Current address: Department of Basic Science, Graduate School of Arts and Sciences The University of Tokyo 3-8-1 Komaba, Meguro-ku Tokyo Japan; ^5^ Current address: Rowland Institute Harvard University Cambridge MA 02142 USA

**Keywords:** Chromophores, Coordination Cages, Energy Transfer, Self-Assembly, Supramolecular Chemistry

## Abstract

A strategy to engineer the stacking of diketopyrrolopyrrole (DPP) dyes based on non‐statistical metallosupramolecular self‐assembly is introduced. For this, the DPP backbone is equipped with nitrogen‐based donors that allow for different discrete assemblies to be formed upon the addition of Pd(II), distinguished by the number of π‐stacked chromophores. A Pd_3_L_6_ three‐ring, a heteroleptic Pd_2_L_2_L′_2_ ravel composed of two crossing DPPs (flanked by two carbazoles), and two unprecedented self‐penetrated motifs (a Pd_2_L_3_ triple and a Pd_2_L_4_ quadruple stack), were obtained and systematically investigated. With increasing counts of stacked chromophores, UV/Vis absorptions red‐shift and emission intensities decrease, except for compound Pd_2_L_2_L′_2_, which stands out with an exceptional photoluminescence quantum yield of 51 %. This is extraordinary for open‐shell metal containing assemblies and explainable by an intra‐assembly FRET process. The modular design and synthesis of soluble multi‐chromophore building blocks offers the potential for the preparation of nanodevices and materials with applications in sensing, photo‐redox catalysis and optics.

## Graphical abstract

Stacking of one, two, three, or four diketopyrrolopyrrole (DPP) dyes was achieved by employing recent progress in Pd(II)‐mediated self‐assembly. Two unprecedented self‐penetrated motifs have been identified: a Pd_2_L_3_ triple and a Pd_2_L_4_ quadruple stack. With increasing stack size, the absorption bathochromically shifts and emission quenches, except for Pd_2_L_2_L′_2_, which stands out with an exceptional luminescence quantum yield of 51 %.

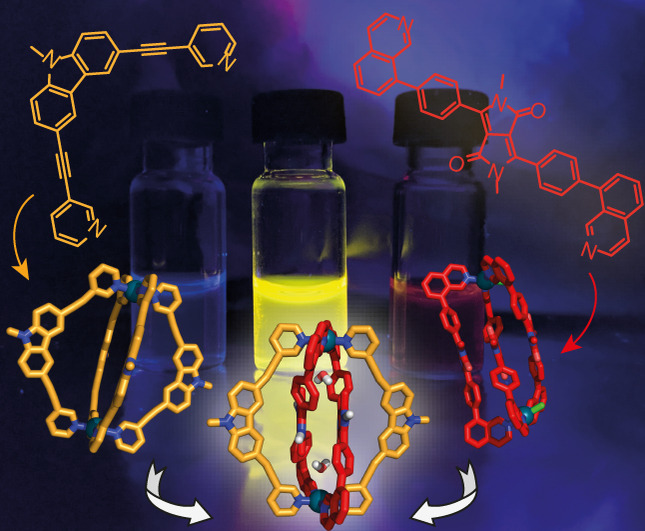

## Introduction

The precise molecular engineering of dye‐based assemblies with control over the spatial co‐arrangement of chromophores is a promising approach towards tailormade optical materials.^[1]^ Understanding and adjusting intermolecular effects between neighboring chromophores in such aggregates is crucial for the development of functional nanoarchitectures for application in fields such as solar energy harvesting and transfer, charge separation, optoelectronics and photo‐redox catalysis. The rational implementation of specific photophysical processes such as FRET pathways, aggregation‐induced emission and non‐linear effects into engineered materials requires control over the spatial placement and mutual orientation of interacting chromophores. Nature masters the precise arrangement of hundreds of photofunctional units, chlorophylls and carotinoids, in its light‐harvesting photosynthetic machinery.^[2]^ From a synthetic side, a variety of different strategies has been developed during the past decades to mimic this high degree of spatial chromophore organization. Besides crystal engineering,[^3^, ^4^] the design of liquid crystalline phases and supramolecular polymers allowing to arrange chromophores in determined patterns has made significant progress.[^5^, ^6^, ^7^] While such dye‐based condensed phases have found entry into many applications, from organic photovoltaic films over OLEDs to laser materials,[^8^, ^9^] there are areas where discrete and soluble multi‐chromophore stacks are beneficial, either because applications are carried out in homogeneous solution (e.g. in photo‐redox catalysis), single entities are immobilized on functional surfaces (e.g. in sensor devices with optical readout) or the solvent‐based delivery of molecular building blocks enables new dimensions in material processing (e.g. hierarchically structured composite films).[^10^, ^11^, ^12^] For example, DNA scaffolds can organize multi‐perylene semiconductor architectures^[13]^ and water‐soluble, dye‐decorated nanoparticles are employed in immunodiagnostics and bioimaging.^[14]^ While covalent organic synthesis is frequently applied to realize discrete multi‐chromophore systems[^15^, ^16^, ^17^, ^18^] supramolecular assembly strategies avoid lengthy syntheses but allow for modular generation of discrete composites.[^19^, ^20^, ^21^] In this respect, molecular tweezers stabilize dye aggregates in nanoscopic clefts,^[22]^ and cucurbit[10]urils incorporate benzothiadiazole trimers with tunable multicolor fluorescence.^[23]^

Recently, coordination‐driven self‐assembly excels over other approaches to realize discrete stacks of aromatic systems under precise control over number and arrangement of components in homo‐ and heteromeric aggregates.[^24^, ^25^, ^26^] Examples include Fujita's “molecular flasks” for combining electron‐donating coronenes and ‐accepting triazines^[27]^ and Jin's alternating stacks of pyrenes and naphthalenes in a metallosupramolecular framework.^[28]^ Lützen et al. created a unique Pd_2_L_4_@Pd_4_L_8_ “cage‐in‐ring” assembly consisting of 12 BODIPY chromophores^[29]^ and we assembled Pd_2_L_4_ cages from a series of coal‐tar dyes such as methylene blue and rhodamine.^[30]^

**Figure 1:**
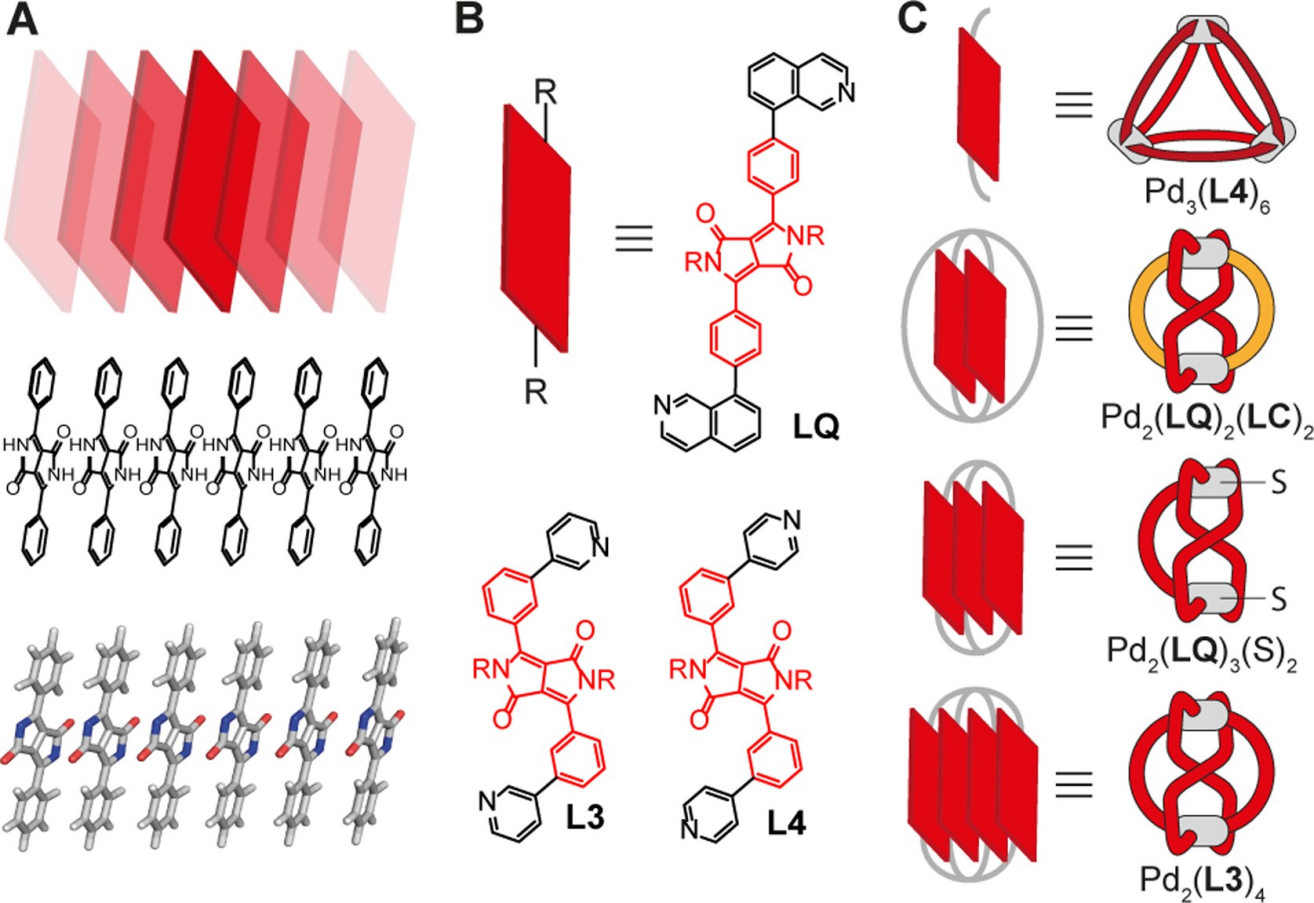
(A) Infinite stack of DPP dyes in a typical pigment's solid‐state structure.^[53]^ (B) DPP‐based ligands **LQ**, **L3** and **L4** equipped with nitrogen‐based donor groups. (C) Coordination of the DPP‐based ligands to Pd(II) cations yields discrete structures characterized by an increasing number of stacked chromophores, namely a Pd_3_(**L4**)_6_ ring, a heteroleptic doubly bridged Pd_2_(**LC**)_2_(**LQ**)_2_ as well as homoleptic singly Pd_2_(**LQ**)_3_(Solvent)_2_ and a doubly bridged figure‐eight Pd_2_(**L3**)_4_ ravel.

Among the “blockbuster” organic chromophores with widespread application, the red diketopyrrolopyrrole (DPP) dyes stand out owing to their low molecular weight, simple synthetic derivatization and exceptional thermal and photo‐stability. They show a strong tendency to form π‐aggregates (Figure 1A), have an electron‐accepting character and high fluorescence quantum yields.[^31^, ^32^, ^33^] DPP derivatives have been widely used as high‐performance pigments (e.g. in plastics, inks, paints and red Ferrari cars), in fluorescent sensors, field‐effect transistors and photovoltaic cells.[^34^, ^35^, ^36^, ^37^, ^38^, ^39^, ^40^, ^41^, ^42^, ^43^, ^44^, ^45^, ^46^, ^47^, ^48^] Relative orientation and interaction between neighboring chromophores control absorption and emission properties and the fate of electronically excited states.[^49^, ^50^] Hence, finding ways to rationally control the stacking of DPP dyes bears potential to develop new materials with tailored properties.

In this direction, we here report a coordination‐driven self‐assembly strategy for the precise engineering of DPP chromophore stacks by incorporating the dye core into three bis‐monodentate ligands, featuring different bonding vectors, that coordinate to square‐planar Pd(II) cations. We employ “coordination sphere engineering” (CSE), allowing to control heteroleptic assembly through steric congestion close to the metal sites, and “shape complementary assembly” (SCA), enabling the integrative combination of building blocks with matching geometry, to engineer a series of unique DPP‐based compounds.[^51^, ^52^] Four motifs with an increasing count of stacked chromophores (no stacking, stacks of two, three, or four DPPs) were obtained, structurally characterized and their optical properties studied (Figure 1B, C). The latter two, a singly and a doubly bridged Figure‐eight ravel, embody homoleptic Pd(II)‐based assembly motifs that have never been reported before.

## Results and Discussion

The facile derivatization of the DPP moiety allows for multiple designs in which two N‐donor groups can be attached to form bis‐monodentate ligands. Depending on the substitution position and orientation, different angles between the bonding vectors can be achieved, leading to defined self‐assembled structures of different shape and size upon addition of square‐planar Pd(II) cations.^[54]^ Here, DPP units were incorporated into bis‐monodentate ligands **L3**, **L4** and **LQ**. Ligands **L4** and **L3** are isomers that only differ in the pyridines’ nitrogen positions (*para* for **L4** and *meta* for **L3**). This difference leads to outward pointing bonding vectors for **L4** and inward pointing ones for **L3**. Ligand **LQ** adopts a much more linear structure and is equipped with two isoquinoline donors which are attached to the para position of the phenyl rings protruding from the DPP core. This leads to a bis‐monodentate ligand with a strongly inward pointing donor orientation.

**Figure 2:**
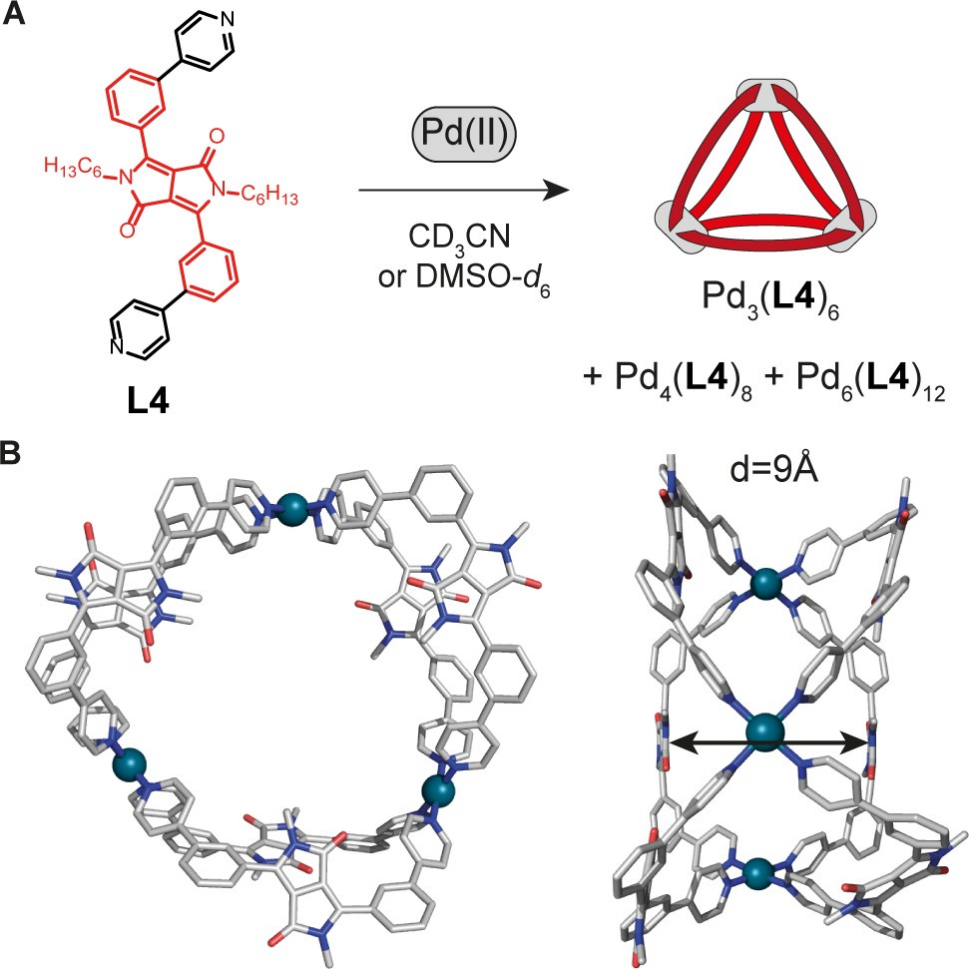
(A) Self‐assembly of ligand **L4** with Pd(II) cations. The major product is the ring [Pd_3_(**L4**)_6_](BF_4_)_6_, minor components are assemblies [Pd_4_(**L4**)_8_](BF_4_)_8_ and [Pd_6_(**L4**)_12_](BF_4_)_12_. (B) X‐ray crystal structure of ring [Pd_3_(**L4**)_6_](BF_4_)_6_ with the shortest distance between neighboring DPP units indicated. Alkyl chains and counterions are omitted for clarity.

Ligands **L4**, **L3** and **LQ** were all synthetized following a similar three‐step process. Starting from succinic acid diisopropyl ester and the properly substituted nitriles (3‐, and 4‐bromobenzonitrile), the core structures of the chromophores were obtained. The solubility of the compounds was improved by N‐alkylation to hinder their H‐bonding ability. Lastly, the ligands were obtained by Suzuki–Miyaura cross‐coupling with 4‐ and 3‐pyridine‐boronic acid 1,3‐propanediol ester or 8‐isoquinoline‐boronic acid, respectively. When **L4** is mixed in a 2 : 1 ratio with [Pd(CH_3_CN)_4_](BF_4_)_2_ in CD_3_CN and heated at 70 °C for 30 min, it forms a mixture of differently sized rings, with the most prominent species being a triangular [Pd_3_(**L4**)_6_](BF_4_)_6_ structure (Figure 2A). In its ^1^H NMR spectra (Figure S15), a typical downfield shift can be observed, indicating coordination of the pyridines to the Pd(II) cations, with the shift being larger the closer the corresponding protons are located to the coordination centers. The spectrum shows two sets of signals, each for one distinct Pd‐based structure, with an integration ratio of 1 : 5. High resolution electrospray ionization mass spectrometry (HR‐ESI‐MS) reveals the [Pd_3_(**L4**)_6_](BF_4_)_6_ stoichiometry as major component while minor amounts of [Pd_4_(**L4**)_8_](BF_4_)_8_ and [Pd_6_(**L4**)_12_](BF_4_)_12_ species could also be detected (Figure S20). By slow diffusion of diethyl ether into the acetonitrile solution, orange crystals were obtained, suitable for synchrotron X‐ray diffraction. The structure is shown in Figure 2B and confirms a three‐membered ring [Pd_3_(**L4**)_6_](BF_4_)_6_. While packing analysis of the solid‐state structure reveals linear inter‐assembly π‐stacking of the DPP backbones along an arrangement of nested rings (Figure S53), no π‐stacking is possible within the individual rings in solution, given the shortest backbone‐to‐backbone distance being about 9 Å (Figure 2B).

**Figure 3:**
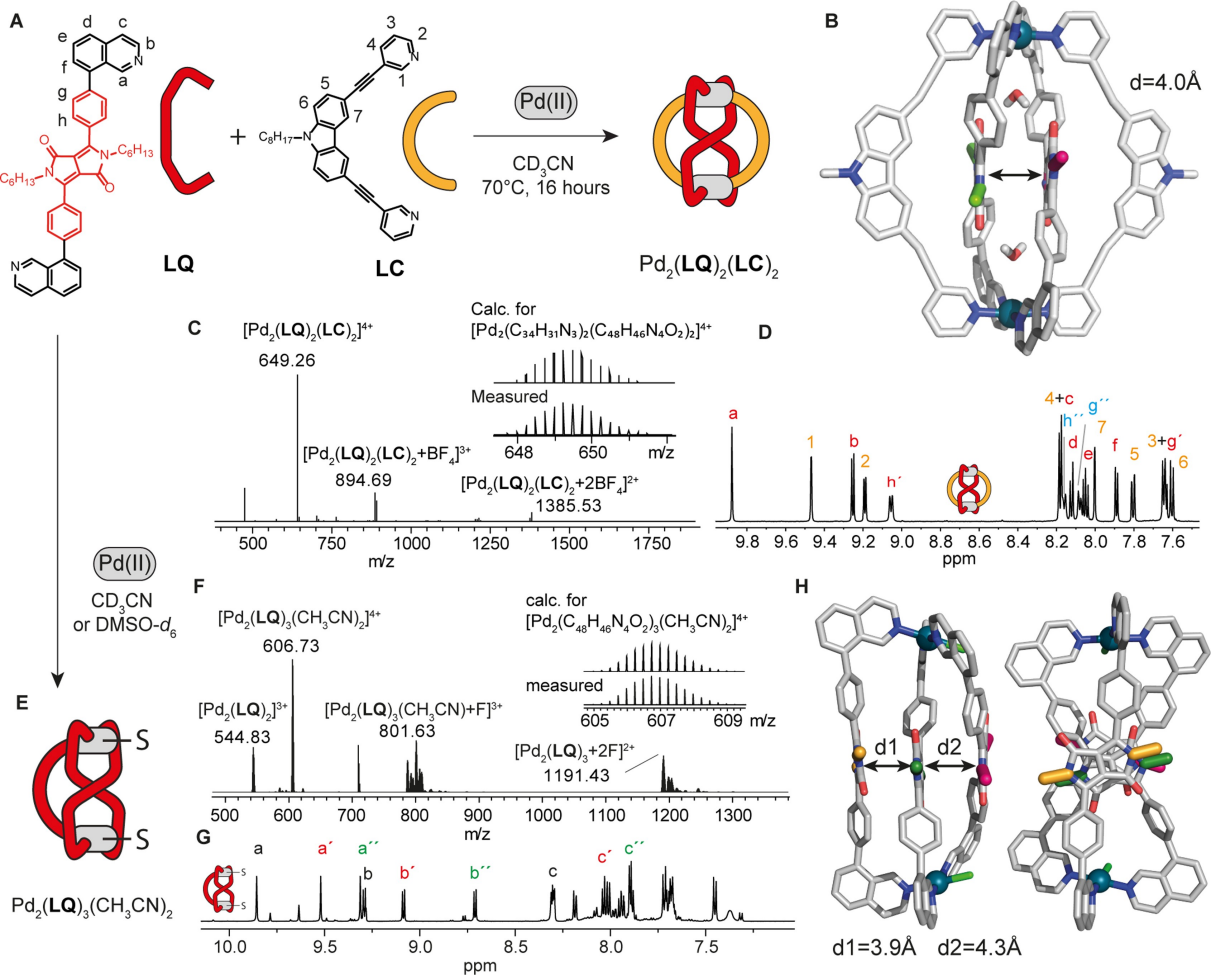
(A) Self‐assembly of ligand **LQ** with **LC** in the presence of a stoichiometric amount of Pd(II) into heteroleptic cage [Pd_2_(**LQ**)_2_(**LC**)_2_](BF_4_)_4_. (B) X‐ray crystal structure of [Pd_2_(**LQ**)_2_(**LC**)_2_](BF_4_)_4_ with the distance between the two DPP backbones indicated. Alkyl chains and counterions are omitted for clarity. N‐CH_2_ moieties of the DPP backbones are colored differently to highlight relative orientation in space. (C) ESI‐MS spectrum of [Pd_2_(**LQ**)_2_(**LC**)_2_+*n*BF_4_]^(4−*n*)+^ with *n*=0–2 (meas. and calc. isotope patterns of [Pd_2_(**LQ**)_2_(**LC**)_2_]^4+^ shown as inset). (D) Enlargement of the aromatic region of the ^1^H NMR spectrum of [Pd_2_(**LQ**)_2_(**LC**)_2_] in CD_3_CN. (E) Self‐assembly of **LQ** with 0.67 equiv. of Pd(II) cations into [Pd_2_(**LQ**)_3_(CH_3_CN)_2_](BF_4_)_4_. (F) ESI‐MS spectrum of [Pd_2_(**LQ**)_3_(CH_3_CN)_2_+*n*F]^(4−*n*)+^ with *n*=0–2 (meas. and calc. isotope patterns for [Pd_2_(**LQ**)_3_(CH_3_CN)_2_]^4+^ shown as inset). (G) Enlarged aromatic region of the ^1^H NMR spectrum of [Pd_2_(**LQ**)_3_(CH_3_CN)_2_](BF_4_)_4_ in CD_3_CN. (H) DFT (B3LYP/LanL2DZ) calculated structure of [Pd_2_(**LQ**)_3_(Cl)_2_]^2+^ with the distances between the DPP backbones indicated.

Next, we aimed at a system that allowed for stacking of exactly two DPP dyes. Therefore, our design was based on a strategy previously reported by our group where bis‐isoquinoline ligands were shown to form self‐penetrating heteroleptic Pd_2_L_2_L′_2_ assemblies when combined with a second ligand.^[55]^ Here, we mixed DPP ligand **LQ**, featuring inward pointing donors, with carbazole‐based bis‐pyridine ligand **LC** in a 1 : 1 ratio and added stoichiometric amounts of [Pd(CH_3_CN)_4_](BF_4_)_2_. Indeed, a single heteroleptic cage product of [Pd_2_(**LQ**)_2_(**LC**)_2_](BF_4_)_4_ stoichiometry was obtained as confirmed by NMR, MS and a crystal structure analysis (Figure 3A—D). The ^1^H NMR spectrum of this assembly (Figure 3D) shows one set of signals equally integrating for each ligand which does neither correspond to free ligands nor to the corresponding homoleptic assemblies (see Figure S24 in the Supporting Information). Signals for phenylene linker protons g and h (Figure 3D) both split two‐fold because ring rotation is hindered and inward and outward pointing hydrogen substituents are distinguished by different chemical environments. Two distinct crystal forms were grown in the same vial by slow vapor diffusion of methyl *tert*‐butyl ether into the solution of [Pd_2_(**LQ**)_2_(**LC**)_2_](BF_4_)_4_ in acetonitrile at room temperature. One of which were needle‐shaped crystals that solved in triclinic space group $P\bar{1}$ with eight cages in the asymmetric unit, the other, with block‐shaped crystals, solved in orthorhombic space group Pbcn with half a cage in the asymmetric unit. One water molecule was found in each of the cage's two small cavities near the Pd sites, modelled with hydrogen bonds to the close‐by (2.8–3.0 Å) carbonyl function of the DPP dyes. Both crystal structures show how the two longer ligands **LQ** cross through the assembly's center from opposite faces, with ligands **LC** bridging the two Pd(II) cations from the outside, to form a *trans*‐[Pd_2_(*anti*‐**LQ**)_2_(**LC**)_2_](BF_4_)_4_ arrangement. Compared to our previously reported assembly Pd_2_L_2_L′_2_ of equal topology,^[55]^ found locked in a distorted, *C*_1_‐symmetrical conformation, the here obtained structure is of higher symmetry (*D*_2_) with planar chirality owing to the crosswise arrangement of the two π‐stacked DPP backbones. Both polymorphs crystallize in a centrosymmetric space group with the two cage enantiomers equally contained.

Having achieved stacking of two backbones in this way, we then further exploited the high tendency of DPP units to π‐stack to construct an assembly containing three stacked DPP chromophores. Therefore, **LQ** alone was combined with Pd(II) cations in a 3 : 2 ratio, leading to a structure where only three of the bis‐monodentate isoquinoline ligands are coordinating the two metal centers, as confirmed by ESI mass spectrometry (Figure 3F). The fourth coordination site on each of the square‐planar palladium cations was found to be occupied by a solvent molecule (or halide ligand). Reason for the formation of this highly unsymmetrical assembly is the large steric demand of the isoquinoline donors around the Pd(II) centers which, in line with our previous observations on comparable systems,[^56^, ^57^] leads to the formation of a [Pd_2_(**LQ**)_3_(CH_3_CN)_2_](BF_4_)_4_ system, even if this violates the “principle of maximum site occupancy”.^[58]^ The ^1^H NMR spectrum recorded in CD_3_CN shows that all ligand signals split into three equally integrating sets (Figure 3G). For other Pd_2_L_3_X_2_ structures (X=solvent or halide), characterized by us before as “bowl structures”,[^56^, ^57^] signals split into two sets of 2 : 1 integral ratio because two oppositely arranged ligands experience an identical chemical environment while the third one in the middle is in a different environment. Here, the most reasonable explanation for the threefold splitting is the strong tendency of this ligand to form a self‐penetrated, π‐stacked arrangement equal to what is observed in the X‐ray structure of [Pd_2_(**LQ**)_2_(**LC**)_2_](BF_4_)_4_, with a further ligand **LQ** adding to the stack from only one side. This decreases the symmetry of the overall structure as schematically depicted in Figure 3E to point group *C*_2_ with the twofold axis going through the middle of the π‐stack. A DFT model of [Pd_2_(**LQ**)_3_(Cl)_2_]^2+^ is shown in Figure 3H.

**Figure 4:**
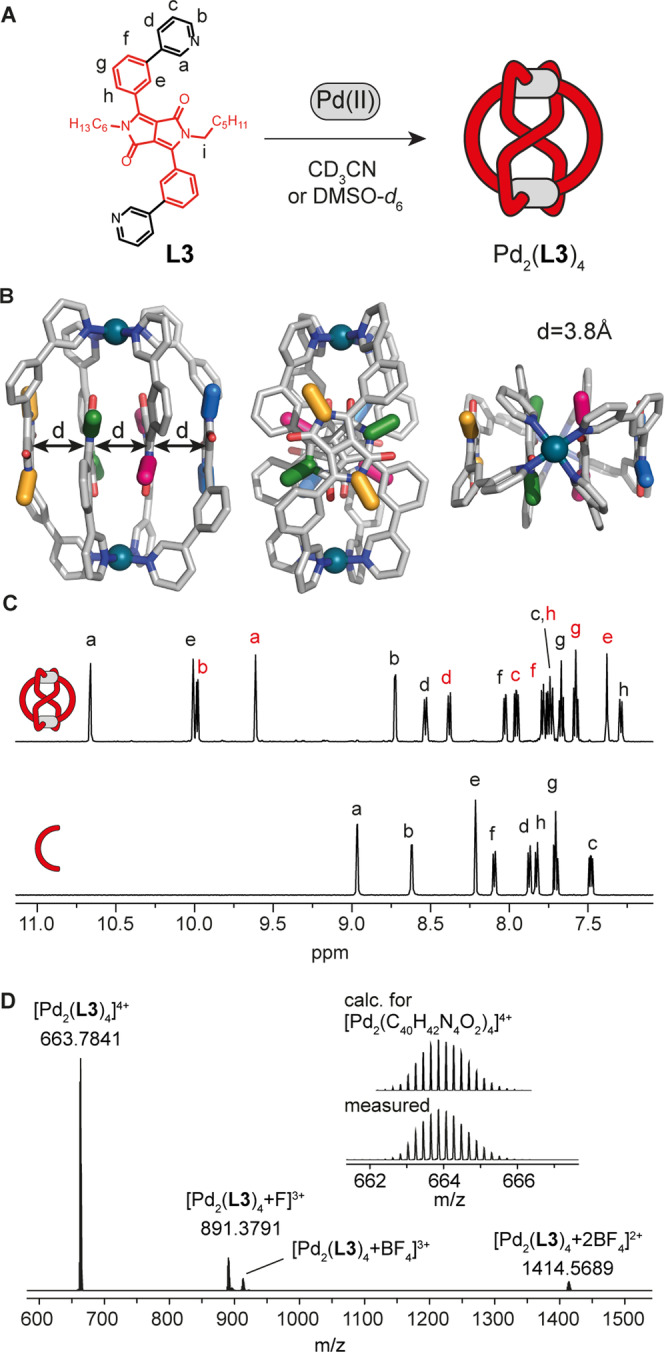
(A) Self‐assembly of ligand **L3** with 0.5 equiv. of Pd(II) cations to give [Pd_2_(**L3**)_4_](BF_4_)_4_. (B) Different views of the X‐ray crystal structure of [Pd_2_(**L3**)_4_](BF_4_)_4_ with the distances between the DPP backbones indicated. Alkyl chains and counterions are omitted for clarity. (C) Enlargement of the aromatic region of the ^1^H NMR spectra in CD_3_CN of **L3** and corresponding Pd‐based assembly [Pd_2_(**L3**)_4_](BF_4_)_4_. (D) ESI‐MS spectrum of [Pd_2_(**L3**)_4_+*n*BF_4_]^(4−*n*)+^ with *n*=0–2. The observed and calculated isotopic patterns of [Pd_2_(**L3**)_4_]^4+^ are shown in the inset.

Lastly, a structure containing an array of four stacked DPP moieties could be obtained employing ligand **L3**. **L3** differs from ligand **L4** in the position of the nitrogen atoms of the pyridine donors, rendering the angle between the bonding vectors much narrower than in **L4**. When ligand **L3** is mixed with 0.5 equiv. of [Pd(CH_3_CN)_4_](BF_4_)_2_, a peculiar combination of a ^1^H NMR spectrum (Figure 4C) and HR‐ESI mass spectrum (Figure 4D) is observed. While the latter clearly indicates formation of a dinuclear [Pd_2_(**L3**)_4_](BF_4_)_4_ species, pointing to the usual lantern‐shaped cage motif,[^54^, ^55^, ^56^, ^57^, ^59^, ^60^] the ^1^H NMR spectrum features splitting of all signals into two sets with equal integration. This observation suggested the formation of an uncommon structural motif in which two different chemical environments for the ligands can be distinguished. Indeed, the solid‐state X‐ray structure revealed again formation of a self‐penetrated Pd_2_L_4_ topology, this time of homoleptic nature, with two inner ligands crossing the assembly's center. The structure, chiral and belonging to point group *D*_2_, is formed as racemic mixture. Consequently, signal splitting for the diastereotopic ‐CH_2_‐ protons Hi is observed (see NMR spectrum in Figure S38). The overall structure is dominated by the π‐stacking of the four ligand DPP backbones, which are facing each other in an orientation rotated by roughly 90° from one to the next backbone.

**Figure 5:**
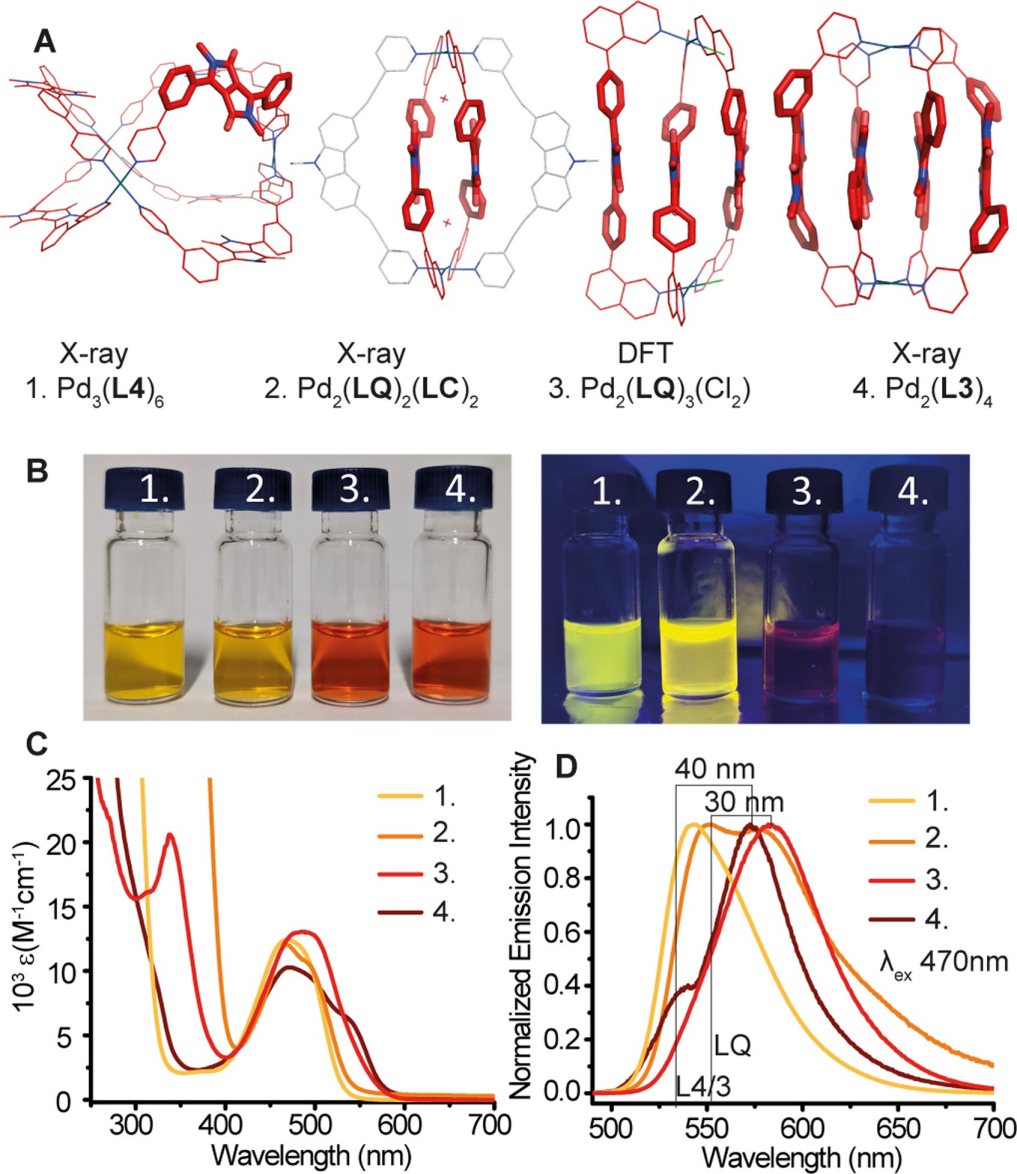
(A) Comparison of the structures of Pd_3_(**L4**)_6_ (X‐ray), Pd_2_(**LQ**)_2_(**LC**)_2_ (X‐ray), [Pd_2_(**LQ**)_3_(Cl)_2_ (DFT) and Pd_2_(**L3**)_4_ (X‐ray) with DPPs highlighted by thicker sticks. (B) Photos of assemblies under ambient (left) and UV light (right). (C) Absorption spectra of the four assemblies in CH_3_CN normalized for a chromophore concentration of 0.35 mM. (D) Normalized emission spectra of the four assemblies in CH_3_CN (λ_ex_=470 nm). Emission maxima of ligands **L4**/**3** and **LQ** indicated to visualize the bathochromic shift, especially for Pd_2_(**LQ**)_3_(CH_3_CN)_2_ and Pd_2_(**L3**)_4_.

In Figure 5A, all four obtained structures with increasing number of stacked dyes are lined up for comparison. Concerning the question, whether the stepwise increase of the number of interacting DPP cores is reflected in the compound's photophysical properties, absorption and emission spectra of the assemblies were recorded (Figure 5C–D). Indeed, the data revealed significant changes depending on the number of directly interacting dyes in the discrete structures. More precisely, the higher the number of stacked chromophores, the more bathochromically shifted are both absorption and emission and the more quenched is the emission. This stacking‐mediated bathochromic shift in the photoluminescence is commonly attributed to the interplay of the short‐ and long‐range couplings between identical chromophores. Increasing the stacking will lead to a splitting of the excited state energy levels and further exciton‐vibronic coupling. As a consequence, the emission will be of lower energy, with multiple vibronic overtones broadening the spectra and allow for additional non‐radiative relaxation pathways.^[61]^ This is indeed already visible to the naked eye (see pictures of the corresponding vials in Figure 5B). Table 1 reports the measured photoluminescence quantum efficiencies (PLQE) for all systems. While the values support the general tendency of increasing emission quenching with growing number of stacked dyes, we observed a notable exception for heteroleptic compound [Pd_2_(**LQ**)_2_(**LC**)_2_](BF_4_)_4_ whose PLQE is with 51 % much higher than expected. To gain further insight, time‐resolved photoluminescence spectra (Figures S48–52, 1 kHz repetition rate, 100 fs long 400 nm excitation pulses, 5 μJ cm^−2^) of the assemblies were compared to those of the respective ligands. Three‐membered ring [Pd_3_(**L4**)_6_](BF_4_)_6_ shows a red‐shifted emission compared to free **L4**. Moreover, ligand **L4** shows a much longer‐lived emission compared to the assembly. On the other hand, [Pd_2_(**LQ**)_2_(**LC**)_2_](BF_4_)_4_ has almost no shift in the emission compared to free **LQ** but here the complex features a longer emission lifetime than the ligand. This, together with the very high PLQE of [Pd_2_(**LQ**)_2_(**LC**)_2_](BF_4_)_4_, suggests a strong communication between the two contained chromophores, carbazole and DPP, which points to an intra‐assembly excitation transfer processes from **LC** to **LQ** upon irradiation, which also appears reasonable in view of the strong overlap between the emission band of **LC** (λ_em_=394 nm) and the absorption band of **LQ** (λ_abs_=474 nm). Next in the series, the emission of [Pd_2_(**LQ**)_3_(CH_3_CN)_2_](BF_4_)_4_ was found to be highly quenched and red‐shifted with respect to the emission of **LQ** (30 nm), which could be indicative of coupling of chromophores or the formation of charge transfer states. In this case, the decay of the emission is again very fast, much faster than for the ligand. The absorption spectrum of [Pd_2_(**L3**)_4_](BF_4_)_4_ is characterized by the observation of two bands, both strongly quenched. The longer wavelength emission is pronouncedly red‐shifted as compared to **L3** (40 nm). Again, both effects point to an electronic coupling of the stacked chromophores or the formation of charge transfer states. Also in this case, the decay of the emission is very fast, much faster than for the ligand.

**Table 1:** Photoluminescence quantum efficiencies (PLQE) for ravels and ligands.^[a]^

Composition	PQLE(%)^[a]^	λ_em_	Δλ
[Pd_3_(**L4**)_6_](BF_4_)_6_	21.0±1.6	543 nm	10 nm
[Pd_2_(**LQ**)_2_(**LC**)_2_](BF_4_)_4_	51.3±1.7	551 nm	0 nm
[Pd_2_(**LQ**)_3_(CH_3_CN)_2_](BF_4_)_4_	3.0±0.4	583 nm	30 nm
[Pd_2_(**L3**)_4_](BF_4_)_4_	0.72±0.07	573 nm	40 nm
**L4**	94.1±0.4	533 nm	–
**L3**	92.9±1.5	533 nm	–
**LQ**	96.1±1.7	551 nm	–

[a] Upon CW excitation at 405 nm at an irradiance of 3 mW cm^−2^. Δλ indicates the shift in emission from the free ligand to the corresponding assemblies.

## Conclusion

Recent progress in non‐statistical assembly strategies towards (multi)functional metal‐mediated compounds were employed to obtain a series of diketopyrrolopyrrole‐based discrete and soluble architectures showing tunable absorption and photoluminescence properties. By combining “coordination sphere engineering” (CSE) and “shape complementary assembly” (SCA) on combinations of different bis‐monodentate N‐donor ligands, we were able to precisely engineer the formation of chromophore stacks with up to four interacting DPPs in dilute solution. Four new compounds, two of which represent never reported homoligand metallosupramolecular motifs (a *C*_2_‐symmetric [Pd_2_(**LQ**)_3_(CH_3_CN)_2_](BF_4_)_4_ “self‐penetrated bowl” and a *D*_2_‐symmetric [Pd_2_(**L3**)_4_](BF_4_)_4_ “self‐penetrated ravel”) were structurally and photophysically characterized. Changes in the optical properties could be related to the number of dyes stacked in the structures. While absorption band shifts and emission quantum yields follow general trends from none to four stacked dyes, compound [Pd_2_(**LQ**)_2_(**LC**)_2_](BF_4_)_4_ was found to show an exceptionally strong emission, in particular for a Pd(II)‐based structure, suggesting the occurrence of an excitation transfer processes in the heteroleptic assembly. The tailored and high‐yielding assembly of prominent chromophores into soluble multi‐dye entities promises to find application in photo‐redox chemistry, optical nano devices and materials, operating in or casted from homogeneous solution phases. Furthermore, gaining precise control over the combination of a low number of interacting chromophores will allow to study photophysical processes in defined multi‐dye systems that are difficult to address in extended materials or ill‐defined polydisperse aggregate mixtures.

## Supporting Information

All data supporting the findings of this study are available within this article and supplemental information files. The accession numbers for the crystallographic data reported in this paper are CCDC: 2168202, 2168203, 2168204, 2168205, 2168206. Copies of these data can be obtained free of charge from the Cambridge Crystallographic Data Centre via www.ccdc.cam.ac.uk/data_request/cif.

## Conflict of interest

The authors declare no conflict of interest.

1

## Supporting information

As a service to our authors and readers, this journal provides supporting information supplied by the authors. Such materials are peer reviewed and may be re‐organized for online delivery, but are not copy‐edited or typeset. Technical support issues arising from supporting information (other than missing files) should be addressed to the authors.

Supporting Information

## Data Availability

The data that support the findings of this study are available from the corresponding author upon reasonable request.

## References

[1] D. Bialas , E. Kirchner , M. I. S. Röhr , F. Würthner , J. Am. Chem. Soc. 2021, 143, 4500. 33719435 10.1021/jacs.0c13245

[2] D. I. G. Bennett , K. Amarnath , G. R. Fleming , J. Am. Chem. Soc. 2013 135, 9164. 23679235 10.1021/ja403685a

[3] R. Haldar , A. Mazel , M. Krstić , Q. Zhang , M. Jakoby , I. A. Howard , B. S. Richards , N. Jung , D. Jacquemin , S. Diring , W. Wenzel , F. Odobel , C. Wöll , Nat. Commun. 2019, 10, 2048. 31053704 10.1038/s41467-019-10011-8 PMC6499792

[4] P. Yu , Y. Zhen , H. Dong , W. Hu , Chem 2019, 5, 2814.

[5] E. Weyandt , L. Leanza , R. Capelli , G. M. Pavan , G. Vantomme , E. W. Meijer , Nat. Commun. 2022, 13, 248. 35017511 10.1038/s41467-021-27831-2 PMC8752679

[6] F. Würthner , C. R. Saha-Möller , B. Fimmel , S. Ogi , P. Leowanawat , D. Schmidt , Chem. Rev. 2016, 116, 962. 26270260 10.1021/acs.chemrev.5b00188

[7] C. R. Benson , L. Kacenauskaite , K. L. VanDenburgh , W. Zhao , B. Qiao , T. Sadhukhan , M. Pink , J. Chen , S. Borgi , C.-H. Chen , B. J. Davis , Y. C. Simon , K. Raghavachari , B. W. Laursen , A. H. Flood , Chem 2020, 6, 1978.

[8] K. Sharma , V. Sharma , S. S. Sharma , Nanoscale Res. Lett. 2018, 13, 381. 30488132 10.1186/s11671-018-2760-6 PMC6261913

[9] Y. Jiang , Y.-Y. Liu , X. Liu , H. Lin , K. Gao , W.-Y. Lai , W. Huang , Chem. Soc. Rev. 2020, 49, 5885. 10.1039/d0cs00037j 32672260

[10] V. Y. Chang , C. Fedele , A. Priimagi , A. Shishido , C. J. Barrett , Adv. Opt. Mater. 2019, 7, 1900091.

[11] R. O. Yathisha , Y. A. Nayaka , SN Appl. Sci. 2020, 2, 451.

[12] M. Gastaldi , F. Cardano , M. Zanetti , G. Viscardi , C. Barolo , S. Bordiga , S. Magdassi , A. Fin , I. Roppolo , ACS Materials Lett. 2021, 3, 1.

[13] J. Gorman , S. R. E. Orsborne , A. Sridhar , R. Pandya , P. Budden , A. Ohmann , N. A. Panjwani , Y. Liu , J. L. Greenfield , S. Dowland , V. Gray , S. T. J. Ryan , S. De Ornellas , A. H. El-Sagheer , T. Brown , J. R. Nitschke , J. Behrends , U. F. Keyser , A. Rao , R. Collepardo-Guevara , E. Stulz , R.-H. Friend , F. Auras , J. Am. Chem. Soc. 2022, 144, 368. 34936763 10.1021/jacs.1c10241 PMC8759064

[14] V. Gubala , G. Giovannini , F. Kunc , M. P. Monopoli , C. J. Moore , Cancer Nanotechnol. 2020, 11, 1.

[15] E. Sebastian , M. Hariharan , ACS Energy Lett. 2022, 7, 696.

[16] J. Nomrowski , X. Guo , X. O. S. Wenger , Chem. Eur. J. 2018, 24, 14084. 30091488 10.1002/chem.201804037

[17] M. Chen , Y. J. Bae , C. M. Mauck , A. Mandal , R. M. Young , M. R. Wasielewski , J. Am. Chem. Soc. 2018, 140, 9184. 29949371 10.1021/jacs.8b04830

[18] J. M. Serin , D. W. Brousmiche , J. M. J. Fréchet , Chem. Commun. 2002, 2605. 10.1039/b207905d 12510259

[19] M. Mahl , M. A. Niyas , K. Shoyama , F. Würthner , Nat. Chem. 2022, 14, 457. 35132223 10.1038/s41557-021-00861-5

[20] H. Gotfredsen , J.-R. Deng , J. M. Van Raden , M. Righetto , J. Hergenhahn , M. Clarke , A. Bellamy-Carter , J. Hart , J. O'Shea , T. D. W. Claridge , F. Duarte , A. Saywell , L. M. Herz , H. L. Anderson , Nat. Chem. 2022, 14, 1436. 36253501 10.1038/s41557-022-01032-w

[21] E. Ubasart , O. Borodin , C. Fuertes-Espinosa , Y. Xu , C. García-Simón , L. Gómez , J. Juanhuix , F. Gándara , I. Imaz , D. Maspoch , M. von Delius , X. Ribas , Nat. Chem. 2021, 13, 420. 33859394 10.1038/s41557-021-00658-6

[22] A. Lohr , M. Grüne , F. Würthner , Chem. Eur. J. 2009, 15, 3691. 19229936 10.1002/chem.200802391

[23] L.-P. Zhang , C.-Z. Liu , M. Liu , S. Lu , S.-B. Yu , Q.-Y. Qi , G.-Y Yang , X. Li , B. Yang , Z.-T Li , Org. Chem. Front. 2022, 9, 6281.

[24] W. Meng , B. Breiner , K. Rissanen , J. D. Thoburn , J. K. Clegg , J. R. Nitschke , Angew. Chem. Int. Ed. 2011, 50, 3479–3483. 10.1002/anie.201100193 21394866

[25] N. Singh , J.-H. Jo , Y. H. Song , H. Kim , D. Kim , M. S. Lah , K.-W Chi , Chem. Commun. 2015, 51, 4492. 10.1039/c4cc09494h 25682749

[26] T. K. Ronson , W. Meng , J. R. Nitschke , J. Am. Chem. Soc. 2017, 139, 9698. 28682628 10.1021/jacs.7b05202

[27] Y. Yamauchi , M. Yoshizawa , M. Akita , M. Fujita , J. Am. Chem. Soc. 2010, 132, 960. 20041719 10.1021/ja904063r

[28] L. Zhang , L. Lin , D. Liu , Y.-J. Lin , Z.-H. Li , G.-X Jin , J. Am. Chem. Soc. 2017, 139, 1653. 28068072 10.1021/jacs.6b11968

[29] M. Käseborn , J. J. Holstein , G. H. Clever , A. Lützen , Angew. Chem. Int. Ed. 2018, 57, 12171. 10.1002/anie.201806814 30040180

[30] I. Regeni , B. Chen , M. Frank , A. Baksi , J. J. Holstein , G. H. Clever , Angew. Chem. Int. Ed. 2021, 60, 5673. 10.1002/anie.202015246 PMC7986857 33245206

[31] M. Grzybowski , D. T. Gryko , Adv. Opt. Mater. 2015, 3, 280.

[32] A. Iqbal , M. Jost , R. Kirchmayr , J. Pfenninger , A. Rochat , O. Wallquist , Bull. Soc. Chim. Belg. 2010, 97, 615.

[33] V. A. S. Almodôvar , A. C. Tomé , Molecules 2021, 26, 4758. 34443350 10.3390/molecules26164758 PMC8401603

[34] M. Grzybowski , E. Glodkowska-Mrowka , T. Stoklosa , D. T. Gryko , Org. Lett. 2012, 14, 2670. 22582959 10.1021/ol300674v

[35] A. Tang , C. Zhan , J. Yao , E. Zhou , Adv. Mater. 2017, 29, 1600013. 10.1002/adma.201600013 27859743

[36] Q. Liu , S. E. Bottle , P. Sonar , Adv. Mater. 2020, 32, 1903882. 10.1002/adma.201903882 31797456

[37] A. Qu , H. Tian , Chem. Commun. 2012, 48, 3039. 10.1039/c2cc17886a 22343975

[38] C. Zhao , Y. Guo , Y. Zhang , N. Yan , S. You , W. Li , J. Mater. Chem. A 2019, 7, 10174.

[39] W. Li , L. Wang , H. Tang , D. Cao , Dyes Pigm. 2019, 162, 934.

[40] M. Kaur , D. H. Choi , Chem. Soc. Rev. 2015, 44, 58. 25186723 10.1039/c4cs00248b

[41] H. Ftouni , F. Bolze , H. de Rocquigny , J.-F. Nicoud , Bioconjugate Chem. 2013, 24, 942. 10.1021/bc300623q 23578090

[42] E. D. Głowacki , H. Coskun , M. A. Blood-Forsythe , U. Monkowius , L. Leonat , M. Grzybowski , D. Gryko , M. S. White , A. Aspuru-Guzik , N. S. Sariciftci , Org. Electron. 2014, 15, 3521. 25642158 10.1016/j.orgel.2014.09.038 PMC4307998

[43] A. Ruiz-Carretero , N. R. Á. Rovelo , S. Militzer , P. J. Mésini , J. Mater. Chem. A 2019, 7, 23451.

[44] Z. Liu , G. Zhang , D. Zhang , Acc. Chem. Res. 2018, 51, 1422. 29771491 10.1021/acs.accounts.8b00069

[45] G. Zhang , K. Liu , Y. Li , M. Yang , Polym. Int. 2009, 58, 665.

[46] V. A. S. Almodôvar , A. C. Tomé , J. Porphyrins Phthalocyanines 2020, 24, 43.

[47] Y. Patil , R. Misra , J. Mater. Chem. C 2019, 7, 13020.

[48] Y.-Z. Wu , Y.-C. Zhang , J.-J. Chen , L.-J Fan , Chin. J. Polym. Sci. 2019, 37, 1092.

[49] M. Más-Montoya , R. A. J. Janssen , Adv. Funct. Mater. 2017, 27, 1605779.

[50] R. Wawrzinek , X. Zhou , M. Ullah , E. B. Namdas , S.-C Lo , Dyes Pigm. 2017, 136, 678.

[51] S. Pullen , J. Tessarolo , G. H. Clever , Chem. Sci. 2021, 12, 7269. 34163819 10.1039/d1sc01226f PMC8171321

[52] W. M. Bloch , G. H. Clever , Chem. Commun. 2017, 53, 8506. 10.1039/c7cc03379f PMC5672845 28661517

[53] J. Mizuguchi , J. Phys. Chem. 2000, 104, 1817.

[54] S. Saha , I. Regeni , G. H. Clever , Coord. Chem. Rev. 2018, 374, 1.

[55] W. M. Bloch , J. J. Holstein , W. Hiller , G. H. Clever , Angew. Chem. Int. Ed. 2017, 56, 8285. 10.1002/anie.201702573 PMC5499718 28544072

[56] R.-J. Li , J. Tessarolo , H. Lee , G. H. Clever , J. Am. Chem. Soc. 2021, 143, 3865. 33673736 10.1021/jacs.0c12188 PMC7975281

[57] B. Chen , J. J. Holstein , S. Horiuchi , W. G. Hiller , G. H. Clever , J. Am. Chem. Soc. 2019, 141, 8907. 31067401 10.1021/jacs.9b02207 PMC6609009

[58] R. Krämer , J. M. Lehn , A. Marquis-Rigault , Proc. Natl. Acad. Sci. USA 1993, 90, 5394. 11607405 10.1073/pnas.90.12.5394 PMC46726

[59] G. H. Clever , W. Kawamura , M. Shionoya , Inorg. Chem. 2011, 50, 4689. 21513283 10.1021/ic200517r

[60] M. Han , D. M. Engelhard , G. H. Clever , Chem. Soc. Rev. 2014, 43, 1848. 24504200 10.1039/c3cs60473j

[61a] T. Kim , C. Lin , J. D. Schultz , R. M. Young , M. R. Wasielewski , J. Am. Chem. Soc. 2022, 144, 11386; 35699940 10.1021/jacs.2c03993

[61b] N. Tang , J. Zhou , L. Wang , M. Stolte , G. Xie , X. Wen , L. Liu , F. Würthner , J. Gierschner , Z. Xie , Nat. Commun. 2023, 14, 1922; 37024474 10.1038/s41467-023-37171-y PMC10079835

[61c] C. Kaufmann , D. Bialas , M. Stolte , F. Würthner , J. Am. Chem. Soc. 2018, 140, 9986; 29992819 10.1021/jacs.8b05490

[61d] A. J. Musser , P. P. Neelakandan , J. M. Richter , H. Mori , R. H. Friend , J. R. Nitschke , J. Am. Chem. Soc. 2017, 139, 12050; 28753299 10.1021/jacs.7b06709 PMC5579544

[61e] I. Heckelmann , Z. Lu , J. C. A. Prentice , F. Auras , T. K. Ronson , R. H. Friend , J. R. Nitschke , S. Feldmann , Angew. Chem. Int. Ed. 2023, 62, e202216729. 10.1002/anie.202216729 PMC10947190 36652344

[62] Deposition numbers 2168202 (for Pd_3_(**L4**)_6_), 2168203 (for Pd_2_(**LC**)_2_(**LQ**)_2_), 2168204 (for Pd_2_(**LC**)_2_(**LQ**)_2_), 2168205 (for Pd_2_(**L3**)_4_) and, 2168206 (for Pd_2_(**L3**)_4_) contain the supplementary crystallographic data for this paper. These data are provided free of charge by the joint Cambridge Crystallographic Data Centre and Fachinformationszentrum Karlsruhe Access Structures service.

