# Supplementary material for: Engineering Soluble Diketopyrrolopyrrole Chromophore Stacks from a Series of Pd(II)‐Based Ravels
Source: Angew Chem Int Ed Engl. 2023 Aug 23;62(40):e202308288. doi: 10.1002/anie.202308288 (PMC10952814; doi:10.1002/anie.202308288)
Supplement: Supplementary file 1 — Supporting Information [file ANIE-62-0-s001.pdf]

Angewandte Chemie
Eine Zeitschrift der Gesellschaft Deutscher Chemiker

Supporting Information

Engineering Soluble Diketopyrrolopyrrole Chromophore Stacks from a Series of Pd(II)-Based Ravels

*I. Regeni, R. Chowdhury, K. Terlinden, S. Horiuchi, J. J. Holstein, S. Feldmann, G. H. Clever**

1. General Methods 2
1.1 NMR 2
1.2 Mass spectrometry 2
1.3 UV-Vis absorption and emission spectroscopy 2
1.4 Computational studies 2
2. Experimental Procedures 2
2.1 Synthesis of the ligands 2
2.1.1 Ligand **L4** 2
2.1.2 Ligand **L3** 5
2.1.3 Ligand **LQ** 6
2.2 Synthesis of the assemblies 9
2.2.1 $[Pd_3(\mathbf{L4})_6](BF_4)_6$ (+ minor components $[Pd_4(\mathbf{L4})_8](BF_4)_8$ + $[Pd_6(\mathbf{L4})_{12}](BF_4)_{12}$) 9
2.2.2 $[Pd_2(\mathbf{LC})_2(\mathbf{LQ})_2](BF_4)_4$ 13
2.2.3 $[Pd_2(\mathbf{LQ})_3(CD_3CN)_2](BF_4)_4$ 16
2.2.4 $[Pd_2(\mathbf{L3})_4](BF_4)_4$ 20
3. Optical Properties and Spectral Characterization 24
3.1 Photoluminescence Quantum Efficiency (PLQE) Measurements 27
4. X-ray crystal structure analysis 31
5. Supplemental References 41

1. General Methods

1.1 NMR

NMR spectroscopic data was measured on the spectrometers Bruker AV 500 Avance NEO and AV 600 Avance III HD. For ^{1}H and ^{13}C NMR spectra, chemical shifts were calibrated to the solvent lock signal. Chemical shifts δ are given in ppm, coupling constants *J* in Hz. The following abbreviations are used to describe signal multiplicity for ^{1}H NMR spectra: s: singlet, d: doublet, t: triplet, dd: doublet of doublets; dt: doublet of triplets; m: multiplet, br: broad. All proton and carbon signals were assigned with the aid of 2D NMR spectra. All spectra were recorded in standard 5 mm NMR tubes at the indicated temperature.

1.2 Mass spectrometry

Mass spectra were measured on Bruker ESI-timsTOF (electrospray ionization-trapped ion mobility-time of flight) and Bruker compact high-resolution LC mass spectrometers (positive mode). For calibration of the TIMS and TOF devices, Agilent ESI-Low Concentration Tuning Mix was used.

1.3 UV-Vis absorption and emission spectroscopy

UV-vis spectra were recorded on a DAD HP-8453 UV-Vis spectrometer; wavelength: 240 nm – 700 nm, step size: 1 nm. Emission spectra were recorded on a Jasco FP-8300 fluorimeter. Cuvette path length 0.2 cm in absorption and 1 cm in emission.

1.4 Computational studies

A model of structure $[Pd_2(\mathbf{LQ})_3(Cl)_2]^{2+}$ was constructed using Wavefunction SPARTAN'18.[1] The MMFF-optimized model was then optimized on semiempirical PM6 level of theory without constraints and then further refined by DFT optimization (B3LYP/LanL2DZ) using GAUSSIAN 16.[2]

2. Experimental Procedures

Where necessary, experiments were performed under argon atmosphere using standard Schlenk techniques. Chemicals and standard solvents were purchased from Sigma Aldrich, Acros Organics, Carl Roth, TCI Europe, VWR, ABCR and used as received, if not mentioned differently. Dry solvents were purchased or purified and dried over absorbent-filled columns on a GS-Systems solvent purification system (SPS). Reactions were monitored with thin layer chromatography (TLC) using silica coated aluminium plates (Merck, silica 60, fluorescence indicator F254, thickness 0.25 mm). For column chromatography, silica (Merck, silica 60, 0.02–0.063 mesh ASTM) was used as the stationary phase. Ligand **LC** and corresponding homoleptic cage $[Pd_2(\mathbf{LC})_4](BF_4)_4$ were synthesized as previously described.[3,4]

2.1 Synthesis of the ligands

2.1.1 Ligand **L4**

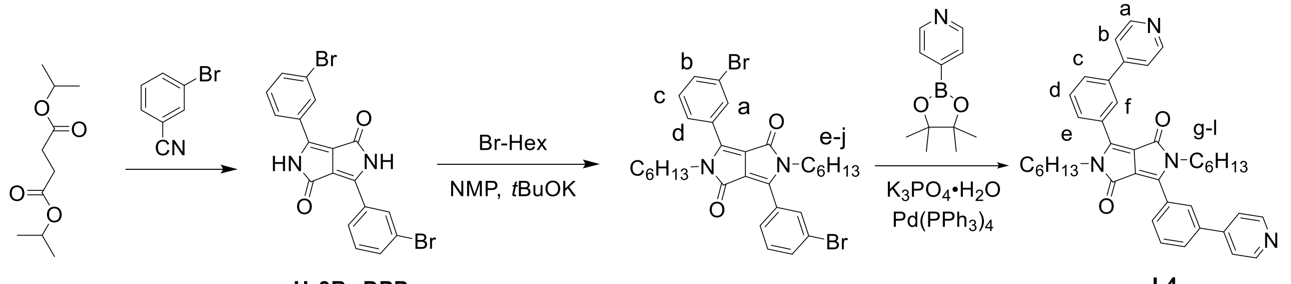

Figure S1: Synthesis of ligand L4.

Synthesis of $H_2$3Br-DPP

Under argon atmosphere, $FeCl_3$ (catalytic amount) was dissolved in 20 mL dry 1-pentanol. Sodium (1.0 g, 43.5 mmol, 4.4 eq.) was added in small chunks and stirred at 90 °C for one hour until it was completely dissolved. The mixture was cooled to 60 °C and 3-bromobenzonitrile (3.92 g, 21.5 mmol, 2.2 eq.) was added and then the mixture heated again to 90 °C. Succinic acid diisopropyl ester (1.98 g, 2 ml, 9.8 mmol, 1 eq.) was added dropwise and the temperature increased to 120 °C for 16 h. After cooling down to room temperature, 10 ml of a 1:1:1-mixture of methanol, water and acetic acid were added and the reaction was

again heated at 120 °C for an hour. The resulting deep red solid was filtered and washed with methanol, water, dichloromethane and diethyl ether. Drying at 70 °C overnight resulted in **$H_2$3BrDPP** as a deep red solid (1.11 g, 2.5 mmol, 25%).

Due to the very poor solubility of the obtained compound, it was not possible to perform a meaningful analytical characterization or to further purify it; therefore, it was directly used for the next step.

Synthesis of 3Br-DPP
$H_2$3Br-DPP (3.4 g, 1.0 equiv., 7.6 mmol) and potassium *tert*-butoxide (1.88 g, 2.2 equiv., 16.8 mmol) in *N*-methyl-2-pyrrolidone (NMP; 30 mL) were heated to 60 °C. 1-bromohexane (6.42 mL, 6 equiv., 45.7 mmol) was slowly added and the mixture was stirred at 60 °C for 24 h. After cooling to room temperature, toluene (150 mL) was added into the reaction mixture and the mixture was washed with water to remove NMP. The organic phase was concentrated using a rotary evaporator. The crude product was purified by column chromatography on silica using dichloromethane as solvent to yield **3Br-DPP** as orange-red polycrystalline powder (1.2 g, 1.9 mmol, 25%).

^{1}H NMR (500 MHz, chloroform-*d*) δ 7.93 (t, *J* = 1.8 Hz, 1H, Ha), 7.77 (dt, *J* = 7.8, 1.3 Hz, 1H, Hb), 7.65 (dd, *J* = 7.3, 1.8 Hz, 1H, Hd), 7.41 (t, *J* = 7.9 Hz, 1H, Hc), 3.76 – 3.69 (m, 2H, He), 1.61 – 1.55 (m, 2H, Hf), 1.22 (qq, *J* = 8.9, 5.3, 3.6 Hz, 6H, Hg-i), 0.88 – 0.80 (m, 3H, Hj).

^{13}C NMR (126 MHz, chloroform-*d*) δ 162.42 (C=O), 147.19 (N-**C**=C), 134.24 (Cd), 131.37 (Ca), 130.62 (Cc), 130.13 (C^q, C-Br), 127.53 (C^q, Ca-**C**-Cd), 123.09 (Cb), 110.34 (N-C=**C**), 42.07(Ce), 31.31 (Cf), 29.55 (Cg), 26.46 (Ch), 22.60 (Ci), 14.08 (Cj).

HR ESI-MS: measured for $(C_{30}H_{34}Br_2N_2O_2)H^+$: 615.1154

calculated: 615.1042

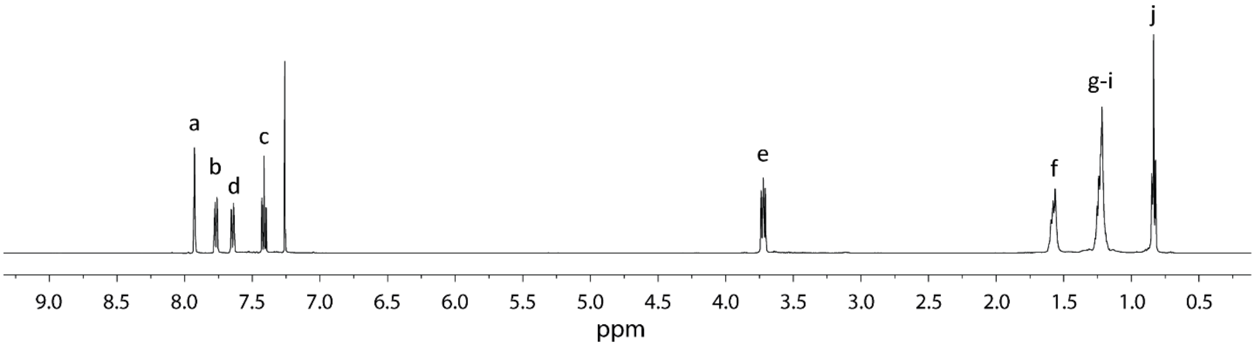

Figure S2: ^{1}H NMR spectrum (500 MHz, 298K, $CDCl_3$) of 3Br-DPP.

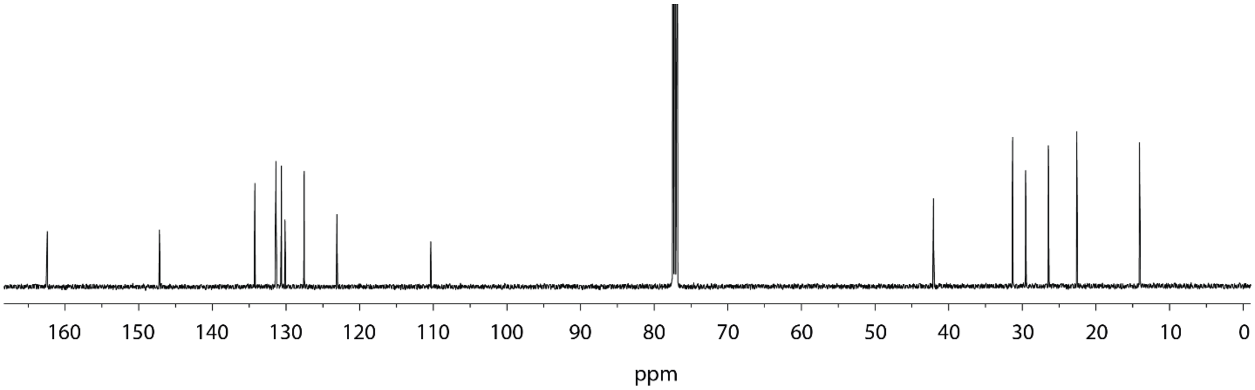

Figure S3: ^{13}C NMR spectrum (126 MHz, 298K, $CDCl_3$) of 3Br-DPP.

Synthesis of L4

4-Pyridine-boronic acid 1,3-propanediol ester (200 mg, 1 mmol, 3.0 equiv.), **3Br-DPP** (200 mg, 0.325 mmol, 1.0 equiv.), and $K_3PO_4 \cdot H_2O$ (2.25 g, 9.77 mmol, 30 equiv.) were suspended in a mixture of 1,4-dioxane/H_2O (4:1, 10 mL). This mixture was degassed using the *Freeze-Pump-Thaw*-method. $Pd(PPh_3)_4$ (23 mg, 0.02 mmol, 0.06 equiv.) was added and the mixture was stirred for 24 h at 90 °C. After cooling down to room temperature, dichloromethane (100 mL) was added and the organic phase was washed with water (3 x 20 mL), dried over $MgSO_4$ and the solvent was removed under reduced pressure. The crude product was purified by column chromatography on silica using dichloromethane as solvent to yield **L4** as orange-red polycrystalline powder (105 mg, 0.172 mmol, 53%). If necessary, **L4** was further purified by recrystallization from DMSO.

^{1}H NMR (500 MHz, chloroform-*d*) δ 8.73 (d, *J* = 5.1 Hz, 2H, Ha), 8.24 (d, *J* = 1.8 Hz, 1H, Hc), 7.80 (m, 2H, He, Hf), 7.66 (m, 3H, Hb, Hd), 3.98 – 3.70 (m, 2H, Hg), 1.64 (p, *J* = 7.6 Hz, 2H, Hh), 1.35 – 1.10 (m, 6H, Hi, Hj, Hk), 0.81 (t, *J* = 6.7 Hz, 3H, Hl).

^{13}C NMR (126 MHz, chloroform-*d*) δ 162.78 (C=O), 150.35 (Ca), 148.17 (N-**C**=C), 147.77 (C^q, Cc-**C**-Cf), 139.12 (C^q, C-Cb), 129.87 (Cd), 129.75 (C^q, Cc-**C**-Cd), 129.22 and 128.62 (Ce and Cf), 128.24 (Cc), 121.96 (Cb), 110.39 (N-C=**C**), 42.21(Cg), 31.36(Ci), 29.67(Ch), 26.53(Cj), 22.60(Ck), 14.07(Cl).

HR ESI-MS: measured for $(C_{40}H_{42}N_4O_2)H^+$: 611.3334

calculated: 611.3381

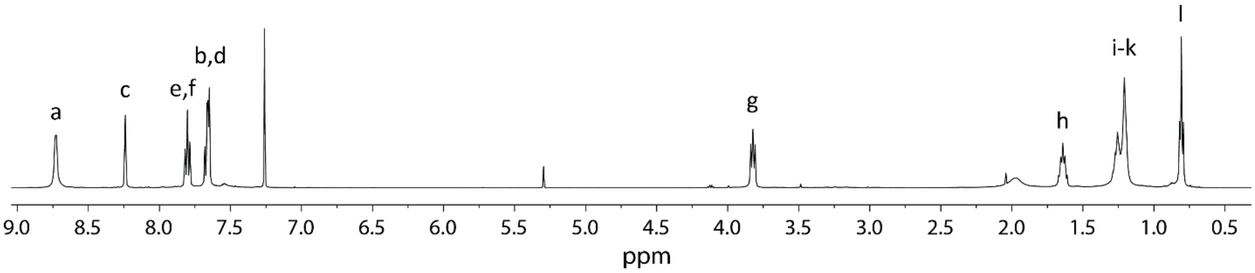

Figure S4: ^{1}H NMR spectrum (500 MHz, 298K, $CDCl_3$) of L4.

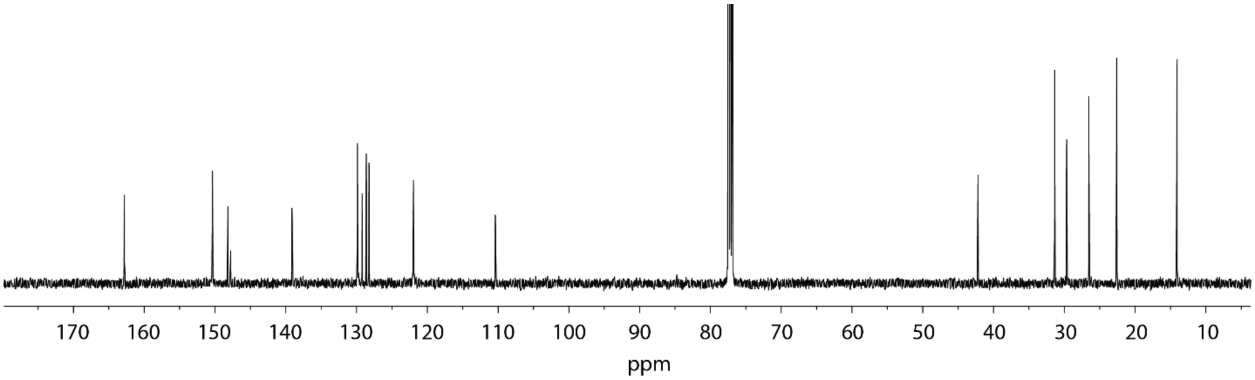

Figure S5: ^{13}C NMR spectrum (126 MHz, 298K, $CDCl_3$) of L4.

2.1.2 Ligand **L3**

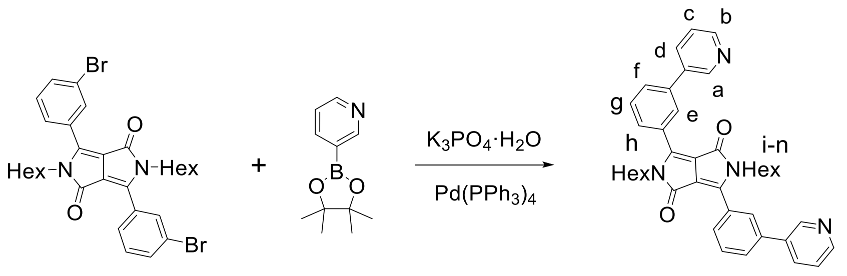

Figure S6: Synthesis of ligand L3.

3-Pyridine-boronic acid 1,3-propanediol ester (200 mg, 1 mmol, 3.0 equiv.), **3Br-DPP** (200 mg, 0.325 mmol, 1.0 equiv.), and $K_3PO_4 \cdot H_2O$ (2.25 g, 1 mmol, 30 equiv.) were suspended in a mixture of 1,4-dioxane/H_2O (4:1, 10 mL). This mixture was degassed using the *Freeze-Pump-Thaw*-method. $Pd(PPh_3)_4$ (23 mg, 0.02 mmol, 0.06 equiv.) was added and it was stirred for 24 h at 90 °C. After cooling down to room temperature, dichloromethane (100 mL) was added and the organic phase was washed with water (3 x 20 mL), dried over $MgSO_4$ and the solvent was removed under reduced pressure. The crude product was purified by column chromatography on silica using dichloromethane as solvent to yield **L3** as orange-red polycrystalline powder (90 mg, 0.147 mmol, 45%).

^{1}H NMR (600 MHz, 298 K, dimethyl sulfoxide-*d*$_6$) δ 9.02 (d, *J* = 2.4 Hz, 1H, Ha), 8.64 (dd, *J* = 4.7, 1.6 Hz, 1H, Hd), 8.25 (d, *J* = 1.8 Hz, 1H, He), 8.19 (dt, *J* = 8.0, 2.0 Hz, 1H, Hb), 7.98 (dt, *J* = 7.8, 1.3 Hz, 1H, Hf), 7.86 (dt, *J* = 7.9, 1.3 Hz, 1H, Hh), 7.75 (t, *J* = 7.8 Hz, 1H, Hg), 7.56 (dd, *J* = 7.9, 4.7 Hz, 1H, Hc), 3.79 (t, *J* = 7.5 Hz, 2H, Hi), 1.48 (p, *J* = 7.3 Hz, 2H, Hj), 1.20 – 1.02 (m, 6H, Hk-m), 0.73 (t, *J* = 6.8 Hz, 3H, Hn).

^{1}H NMR (500 MHz, 298 K, chloroform-*d*) δ 8.94 (d, *J* = 2.3 Hz, 1H), 8.65 (dd, *J* = 4.9, 1.6 Hz, 1H), 8.14 (t, *J* = 1.8 Hz, 1H), 8.03 (ddd, *J* = 7.9, 2.4, 1.6 Hz, 1H), 7.84 – 7.71 (m, 2H), 7.65 (t, *J* = 7.8 Hz, 1H), 7.43 (ddd, *J* = 7.9, 4.8, 0.8 Hz, 1H), 3.93 – 3.71 (m, 2H), 1.78 – 1.56 (m, 2H), 1.35 – 1.09 (m, 6H), 0.91 – 0.72 (m, 3H).

^{1}H NMR (600 MHz, 298 K, acetonitrile-*d*$_3$) δ 9.04 – 8.88 (m, 1H), 8.62 (dd, *J* = 4.8, 1.6 Hz, 1H), 8.21 (t, *J* = 1.9 Hz, 1H), 8.10 (ddd, *J* = 7.9, 2.4, 1.6 Hz, 1H), 7.93 – 7.79 (m, 2H), 7.70 (t, *J* = 7.8 Hz, 1H), 7.48 (ddd, *J* = 8.0, 4.8, 0.9 Hz, 1H), 3.90 – 3.72 (m, 2H), 1.54 (p, *J* = 7.4 Hz, 2H), 1.25 – 1.11 (m, 6H), 0.78 (t, *J* = 6.8 Hz, 3H).

^{13}C NMR (151 MHz, 298 K, dimethyl sulfoxide-*d*$_6$) δ 161.62 (**C**=O), 149.04 (**C**d), 147.81 (**C**a), 147.75 (N-**C**=C), 137.73 (Cf-**C**-Ce), 134.74 (Cd-**C**-Ca), 134.33 (**C**b), 129.87(**C**g), 129.76(**C**f), 128.50 (Ch-**C**-Ce), 127.76 (**C**h), 127.33 (**C**e), 124.03 (**C**c), 109.06 (N-C=**C**), 40.94 (Ci), 30.47 (Cl), 28.54 (Cj), 25.62 (Ck), 21.83 (Cm), 13.74 (Cn).

^{13}C NMR (151 MHz, 298 K, acetonitrile-*d*$_3$) δ 163.30, 150.11, 149.20, 149.01, 139.42, 136.37, 135.34, 130.72, 130.68, 130.06, 128.85, 128.81, 124.80, 110.89, 42.33, 31.87, 29.91, 26.93, 23.14, 14.20.

HR ESI-MS:	measured for $(C_{40}H_{42}N_4O_2)H^+$:	611.3334
	calculated:	611.3381

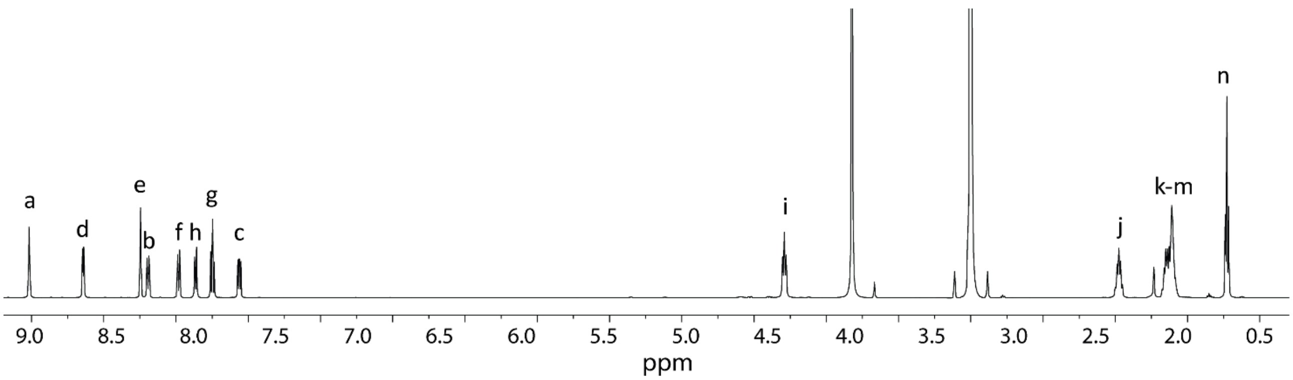

Figure S7: ^{1}H NMR spectrum (600 MHz, 298K, DMSO-d_6) of L3.

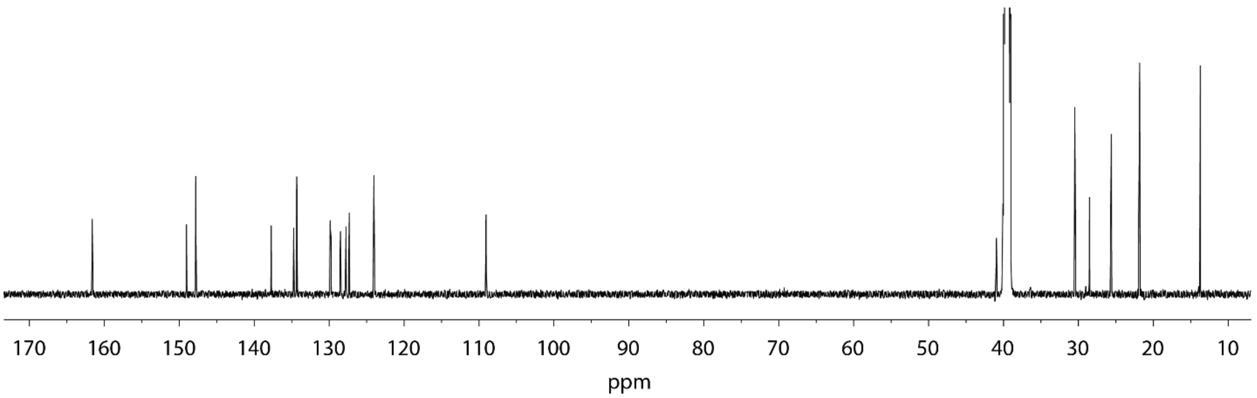

Figure S8: ^{13}C NMR spectrum (151 MHz, 298K, DMSO-d_6) of L3.

2.1.3 Ligand **LQ**

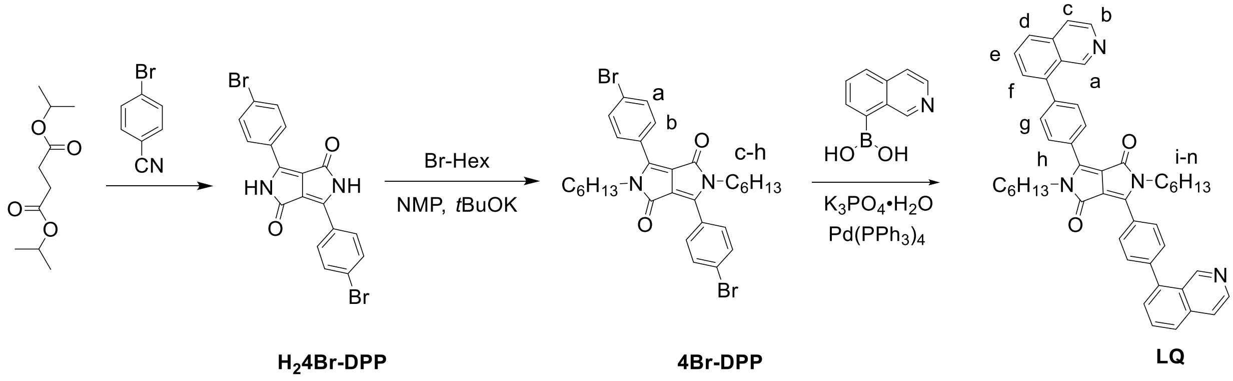

Figure S9: Synthesis of ligand LQ.

Synthesis of $H_2$4Br-DPP

Potassium *tert*-butoxide (2.77 g, 25 mmol, 2.5 equiv.) was dissolved in dry *tert*-amyl alcohol (35 mL) in a 250 mL Schlenk flask under argon atmosphere. The solution was heated to 110 °C for 5 min and then 4-bromobenzonitrile (3.96 g, 22 mmol, 2.2 equiv.) was added. Diisopropyl succinate (1.98 g, 2.0 mL, 9.8 mmol, 1 equiv.) was dissolved in *tert*-amyl alcohol (16 mL) and the solution was slowly added to the reaction mixture via a dropping funnel. The mixture was stirred at 110 °C for 14 h. After cooling to room temperature, ethanol (150 mL) and acetic acid (8 mL) were added to the mixture. The precipitate was collected by filtration and rinsed with ethanol until no colour remained in the filtrate. Subsequently, the obtained

precipitate was dried under vacuum for 24 h to obtain the product as a dark red solid (2.2 g, 4.9 mmol, 50 %).

Due to the very poor solubility of the obtained compound it was not possible to perform a meaningful analytical characterization or to further purify it; therefore, it was directly used for the next step.

Synthesis of 4Br-DPP

Under argon atmosphere **$H_2$4Br-DDP** (1.62 g, 3.63 mmol, 1 equiv.) and potassium carbonate (1.5 g, 10.8 mmol) were stirred in 30 mL of dry DMF for 1 h at 120 °C in a 120 mL Schlenk flask. Then 18-crown-6 (5 mg, 0.02 mmol) was added first followed by 1-bromohexane (1.4 g, 1.19 mL, 8.48 mmol, 2.3 equiv.) and the mixture was stirred for 6 h at 120 °C. After cooling down to room temperature, 50 mL water were added and extracted with 20 mL of chloroform. The organic phase was washed four times with 50 mL water and dried over $MgSO_4$. The crude product was purified via column chromatography on silica gel using pentane:ethyl acetate 99:1 as eluent. The product was obtained as an orange-red powder (0.98 g, 1.6 mmol, 44 %).

^{1}H NMR (700 MHz, chloroform-*d*) δ 7.73 – 7.58 (m, 4H, Ha, Hb), 3.79 – 3.66 (m, 2H, Hc), 1.67 – 1.48 (m, 2H, Hd), 1.28 – 1.15 (m, 6H, He-g), 0.83 (t, *J* = 6.8 Hz, 3H, Hh).

^{13}C NMR (176 MHz, chloroform-*d*) δ 162.56 (C=O), 147.57 (N-**C**=C), 132.38 (Ca), 130.22 (Cb), 127.09 and 125.95 (C^q, C-Br and C^q, C-Cb), 110.07 (N-C=**C**), 42.02 (Cc), 31.32 (Ce), 29.52 (Cd), 26.49 (Cf), 22.58 (Cg), 14.07 (Ch).

HR ESI-MS:	measured for $(C_{30}H_{34}Br_2N_2O_2)H^+$:	615.1084
	calculated:	615.1042

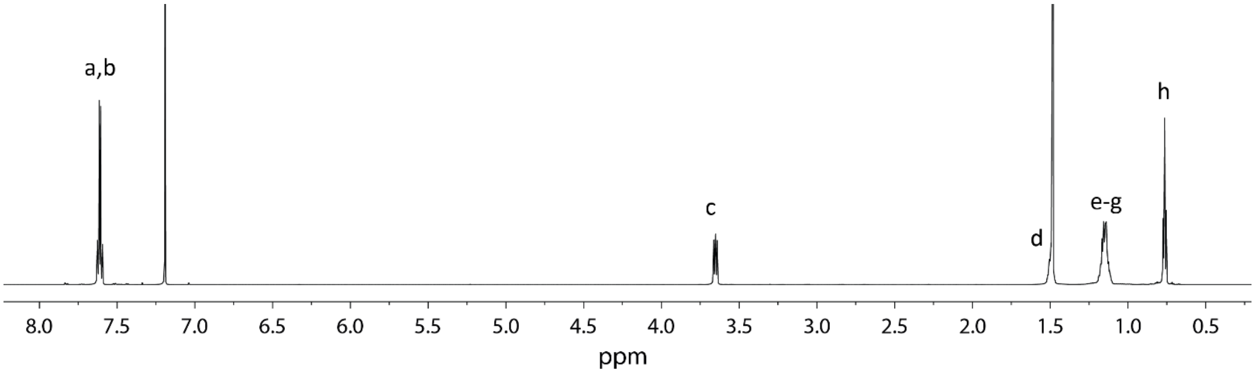

Figure S10: ^{1}H NMR spectrum (700 MHz, 298K, $CDCl_3$) of 4Br-DPP.

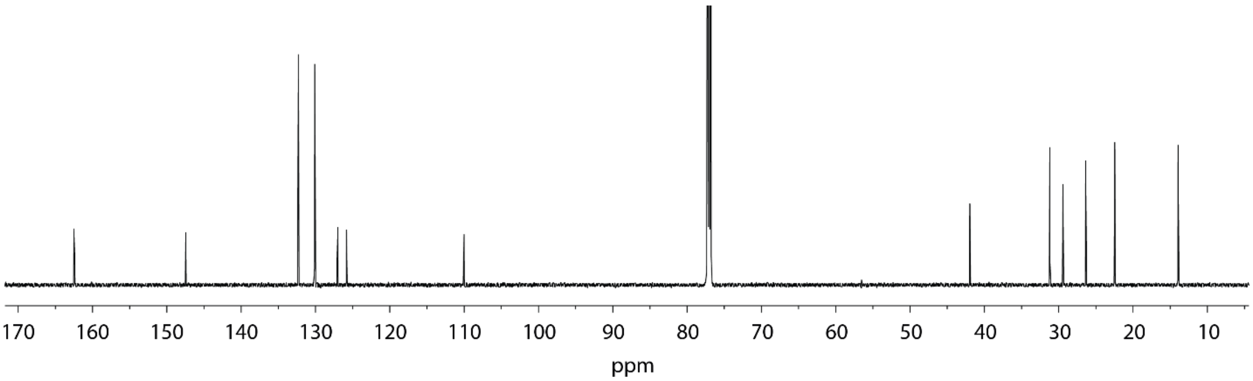

Figure S11: ^{13}C NMR spectrum (176 MHz, 298K, $CDCl_3$) of 4Br-DPP.

Synthesis of LQ

Isoquinoline-8-boronic acid (110 mg, 0.63 mmol, 3.0 equiv.), **4Br-DPP** (130 mg, 0.212 mmol, 1.0 equiv.), and $K_3PO_4 \cdot H_2O$ (1.46 g, 6.35 mmol, 30 equiv.) were suspended in a mixture of 1,4-dioxane/H_2O (4:1, 10 mL). This mixture was degassed using the *Freeze-Pump-Thaw*-method. $Pd(PPh_3)_4$ (25 mg, 0.021 mmol, 0.1 equiv.) was added and the mixture was stirred for 24 h at 90 °C. After cooling down to room temperature, dichloromethane (100 mL) was added and the organic phase was washed with water (3 x 20 mL), dried over $MgSO_4$ and the solvent was removed under reduced pressure. The crude product was purified by column chromatography on silica using dichloromethane as solvent to yield **LQ** as orange-red polycrystalline powder (55 mg, 0.77 mmol, 37%). If necessary, **LQ** was further purified by recrystallization from DMSO.

^{1}H NMR (700 MHz, chloroform-*d*) δ 9.38 (s, 1H, Ha), 8.60 (d, *J* = 5.7 Hz, 1H, Hb), 8.04 (d, *J* = 8.2 Hz, 2H, Hh), 7.89 (d, *J* = 8.3 Hz, 1H, Hd), 7.78 (dd, *J* = 8.1, 7.0 Hz, 1H, He), 7.74 (m, 3H, Hc, Hg), 7.60 (d, *J* = 6.5 Hz, 1H, Hf), 4.00 – 3.80 (m, 2H, Hi), 1.72 (p, *J* = 7.6 Hz, 2H, Hj), 1.42 – 1.19 (m, 6H, Hk, Hl, Hm), 0.85 (t, *J* = 6.9 Hz, 3H, Hn).

^{13}C NMR (176 MHz, chloroform-*d*) δ 162.82 (C=O), 150.83 (Ca), 148.08 (C-**C**=N), 143.23 (Cb), 141.64 (C^q, C-Ch), 139.87(C^q, C-Cg), 136.29 (C^q, Cc-**C**-Cd), 130.64 (Cg), 129.92 (Ce), 128.92 (Ch), 128.37 (Cf), 127.84 (C^q, C-Cf), 126.73 (Cd), 126.39 (C^q, C-Ca), 120.64 (Cc), 110.21 (**C**-C=N), 42.22 (Ci), 31.26(Cl), 29.57(Cj), 26.46 (Ck), 22.49 (Cm), 13.98 (Cn)

HR ESI-MS:	measured for $(C_{48}H_{46}N_4O_2)H^+$:	710.3566
	calculated:	710.3615

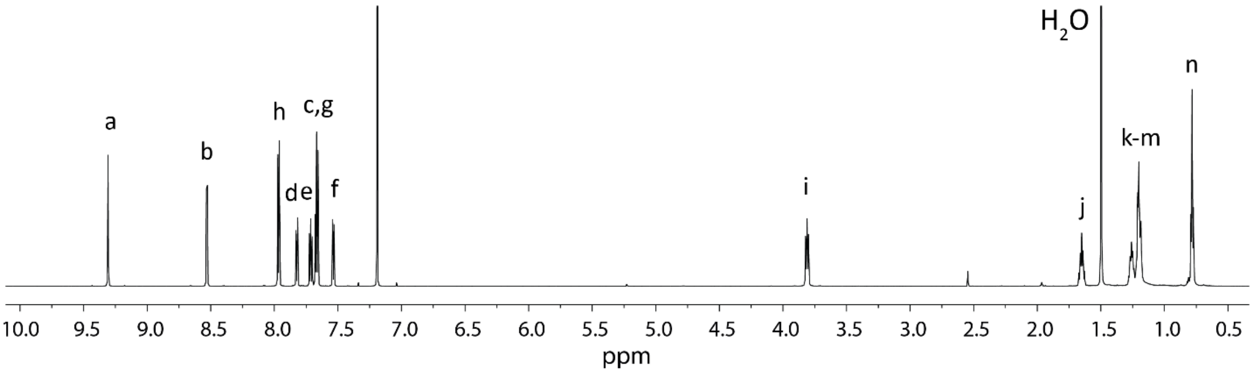

Figure S12: ^{1}H NMR spectrum (700 MHz, 298K, $CDCl_3$) of LQ.

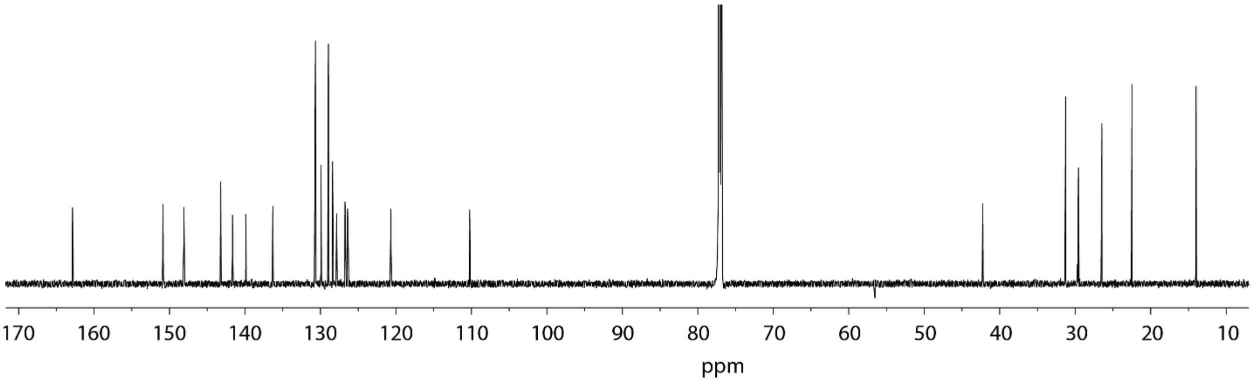

Figure S13: ^{13}C NMR spectrum (176 MHz, 298K, $CDCl_3$) of LQ.

2.2 Synthesis of the assemblies

2.2.1 $[Pd_3(\mathbf{L4})_6](BF_4)_6$ (+ minor components $[Pd_4(\mathbf{L4})_8](BF_4)_8$ + $[Pd_6(\mathbf{L4})_{12}](BF_4)_{12}$)

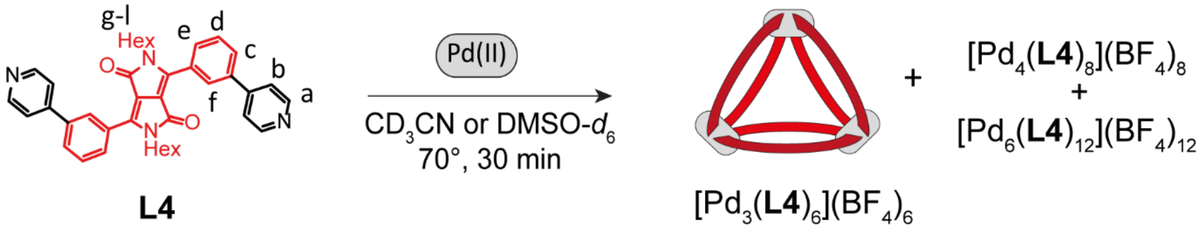

Figure S14: Formation of the trimer $[Pd_3(L4)_6](BF_4)_6$ as main component and $[Pd_4(L4)_8](BF_4)_8$ and $[Pd_6(L4)_{12}](BF_4)_{12}$ as minor components, upon addition of 0.5 equiv. Pd(II) cations to ligand L4.

To 450 µL of a 3.11 mM solution of ligand **L4** in CD_3CN, 50 µL of a 15 mM solution of $[Pd(CH_3CN)_4](BF_4)_2$ in CD_3CN are added. The mixture was than heated to 70 °C for 30 min to afford a mixture of trimer $[Pd_3(\mathbf{L4})_6](BF_4)_6$ as main component and $[Pd_4(\mathbf{L4})_8](BF_4)_8$ and $[Pd_6(\mathbf{L4})_{12}](BF_4)_{12}$ as minor components in the equilibrated solution. Using DMSO-d_6 as a solvent led to a mixture of $[Pd_3(\mathbf{L4})_6](BF_4)_6$ and $[Pd_4(\mathbf{L4})_8](BF_4)_8$ assemblies.

^{1}H NMR (700 MHz, acetonitrile-d_3) δ 9.31 – 9.15 (m, 2H, Ha), 8.20 – 8.16 (m, 1H, Hf), 8.10 – 7.84 (m, 4H, Hb, Hc, He), 7.82 – 7.70 (m, 1H, Hd), 3.79 (m, 2H, Hg), 1.42 – 1.30(m, 2H, Hh), 1.21 – 0.98 (m, 6H, Hi, Hj, Hk), 0.76 – 0.52 (m, 3H, Hl). (only signals of major component listed)

^{1}H NMR (500 MHz, dimethyl sulfoxide-d_6) δ 9.60 – 9.40 (m, 2H, Ha), 8.35 – 8.09 (m, 1H, Hf), 8.06 – 7.86 (m, 4H, Hb, Hc, He), 7.85-7.75 (m, 1H, Hd), 4.03 – 3.49 (m, 2H, Hg), 1.25 – 1.15 (m, 2H, Hh), 1.10 – 0.67 (m, 6H, Hi, Hj, Hk), 0.83 – 0.28 (m, 3H, Hl). (only signals of major component listed)

^{13}C NMR (176 MHz, acetonitrile-d_3) δ 172.52, 162.65, 151.98, 151.65, 147.81, 135.96, 125.32, 31.30, 31.28, 29.27, 26.28, 22.62, 22.57, 22.55, 13.79, 13.72. (only signals of major component listed)

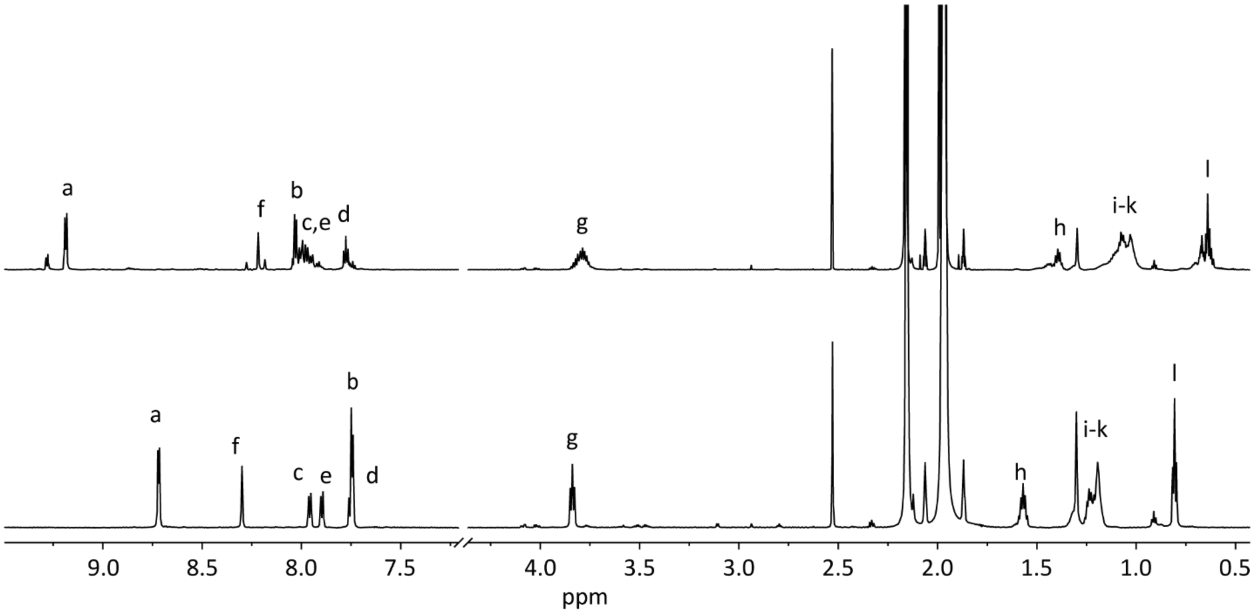

Figure S15: Stacked ^{1}H NMR spectra (700 MHz, 298K, CD_3CN) of ligand L4 and corresponding trimer $[Pd_3(L4)_6](BF_4)_6$ (as main component) upon addition of 0.5 equiv. of Pd(II) in CD_3CN .

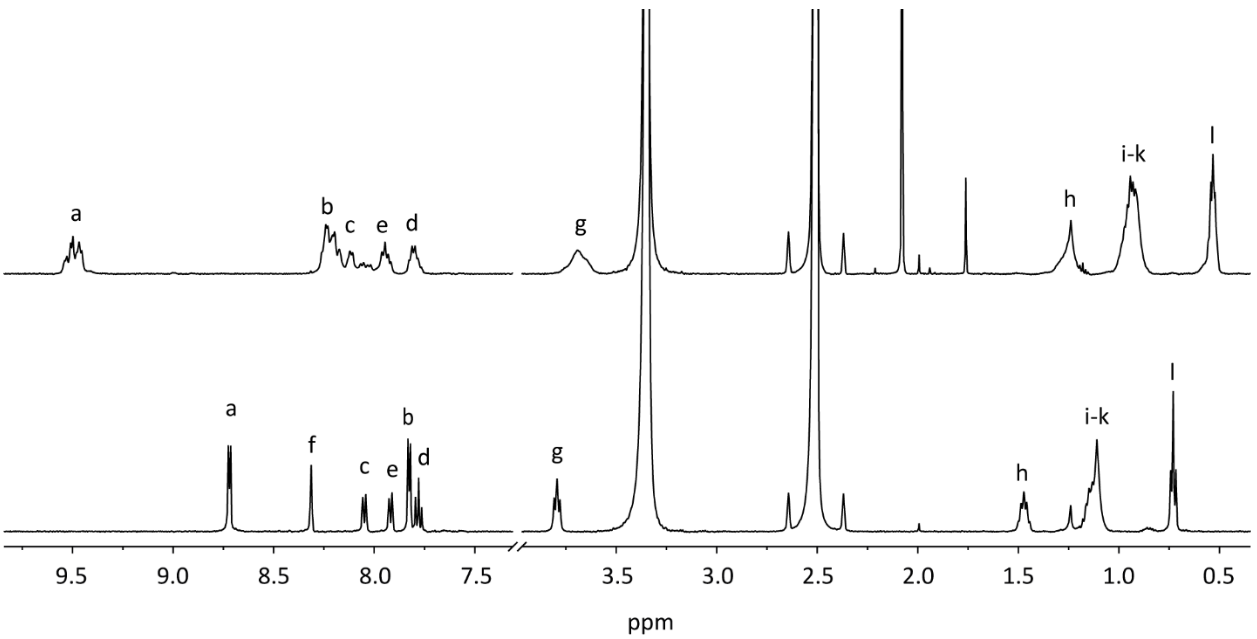

Figure S16: Stacked ^{1}H NMR (500 MHz, 298K, DMSO-d_6) spectra of ligand L4 and corresponding mixture solution of $[Pd_3(L4)_6](BF_4)_6$ and $[Pd_4(L4)_8](BF_4)_8$ upon addition of 0.5 equiv. of Pd(II) in DMSO-d_6.

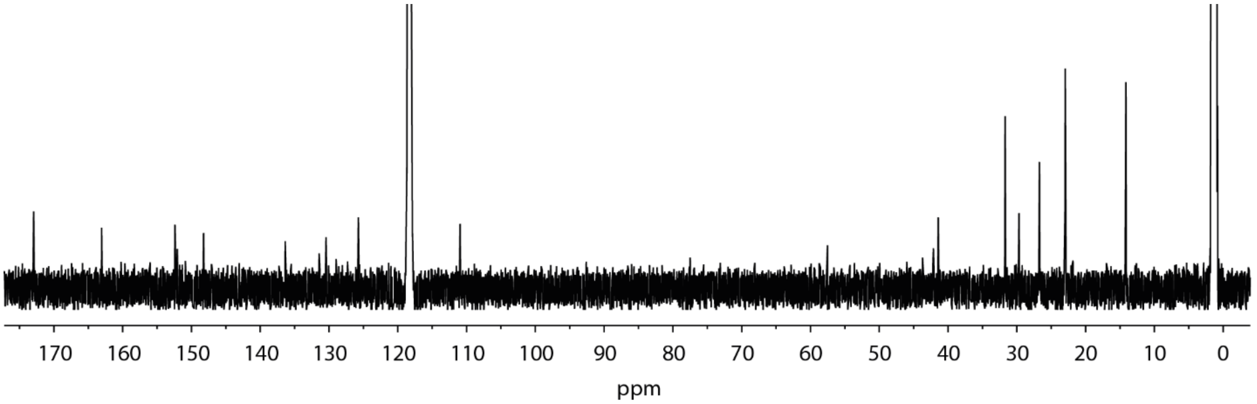

Figure S17: ^{13}C NMR spectrum (176 MHz, 298K, CD_3CN) of $[Pd_3(L4)_6](BF_4)_6$ (as main component) upon addition of 0.5 equiv. of Pd(II) in CD_3CN to L4.

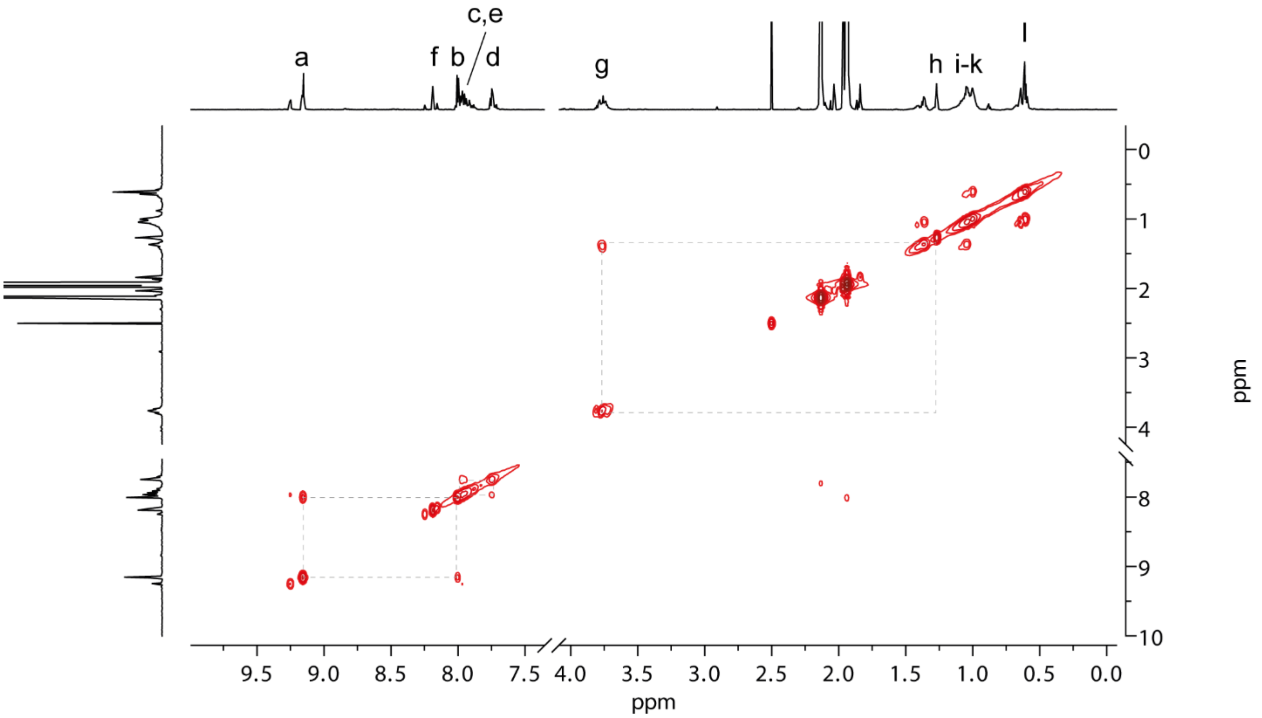

Figure S18: ^1H-^1H COSY NMR (700 MHz, 298K, CD_3CN) spectrum of $[Pd_3(L4)_6](BF_4)_6$ (as main component).

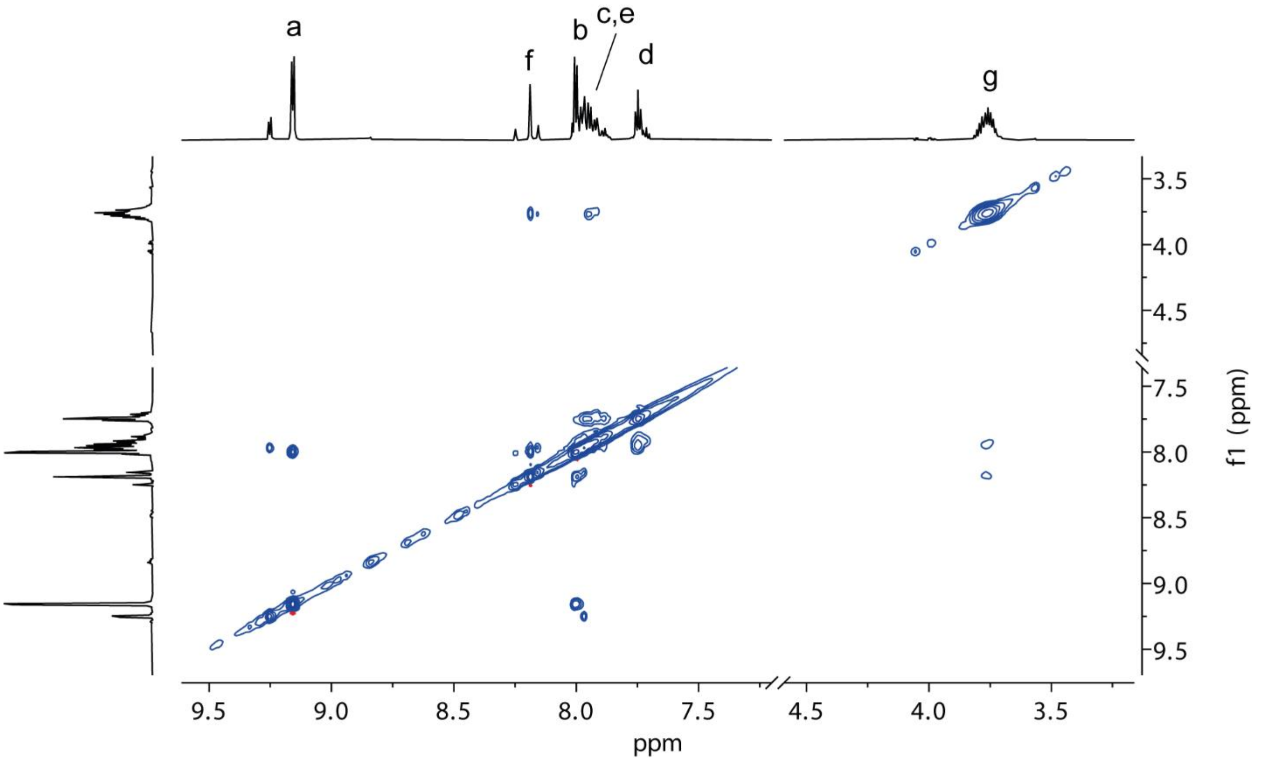

Figure S19: ^1H-^1H NOESY NMR (700 MHz, 298K, CD_3CN) spectrum of $[Pd_3(L4)_6](BF_4)_6$ (as main component).

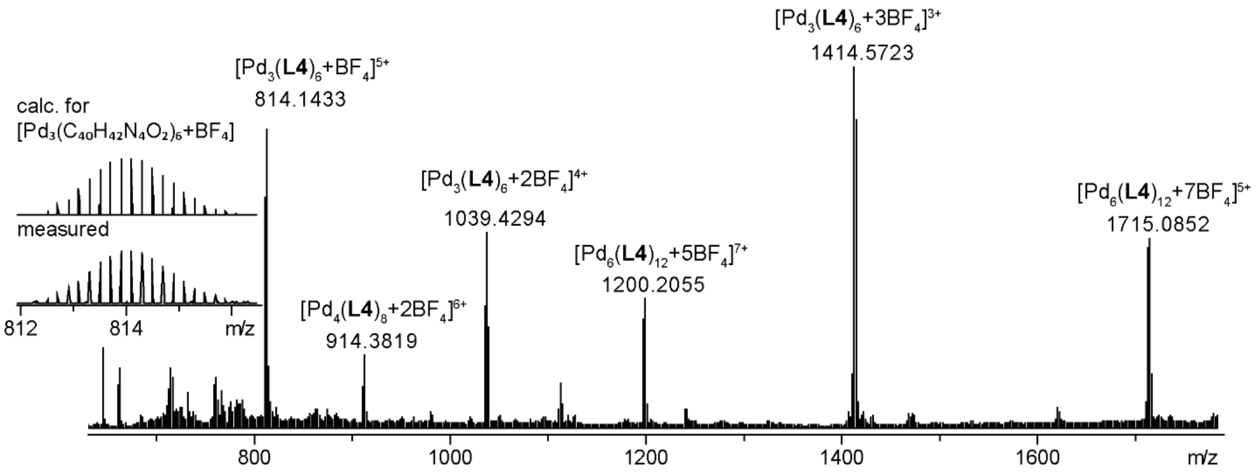

Figure S20: ESI-MS spectrum of an acetonitrile solution of L4 with 0.5 equiv. Pd(II). The observed peaks correspond to $[Pd_3(L4)_6+nBF_4]^{(6-n)+}$ with n=1-3, $[Pd_4(L4)_8+2BF_4]^{6+}$ and $[Pd_6(L4)_{12}+nBF_4]^{(12-n)+}$ with n=5,7. The observed and calculated isotopic patterns of $[Pd_3(L4)_6+BF_4]^{5+}$ are shown in the inset.

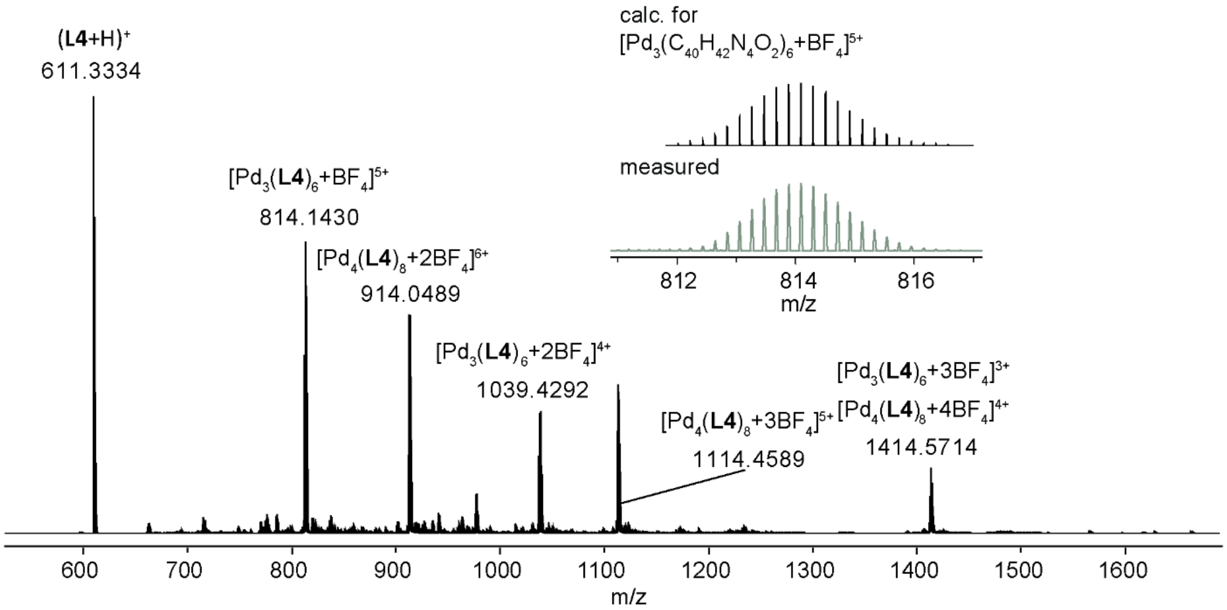

Figure S21: ESI-MS spectrum of a DMSO solution of L4 with 0.5 equiv. Pd(II). The observed peaks correspond to $[Pd_3(L4)_6+nBF_4]^{(6-n)+}$ with n=1-3 and $[Pd_4(L4)_8+nBF_4]\ ]^{(8-n)+}$ with n=2-4. The observed and calculated isotopic patterns of $[Pd_3(L4)_6+BF_4]^{5+}$ are shown in the inset.

2.2.2 $[Pd_2(\mathbf{LC})_2(\mathbf{LQ})_2](BF_4)_4$

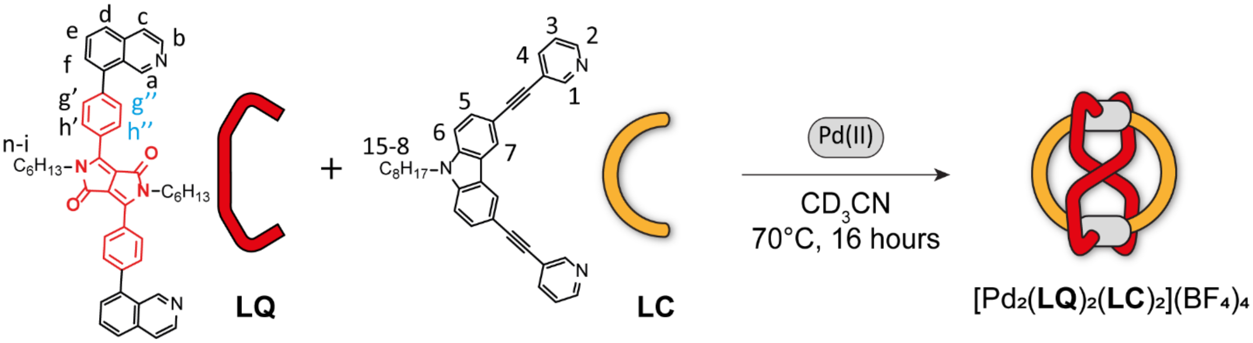

Figure S22: Formation of heteroleptic assembly $[Pd_2(LQ)_2(LC)_2](BF_4)_4$.

A mixture of ligand **LQ** (250 µL of a 2.8 mM solution in CD_3CN) and ligand **LC** (250 µL of a 2.8 mM solution in CD_3CN) and $[Pd(CH_3CN)_4](BF_4)_2$ (50 µL of a 15 mM solution in CD_3CN) was heated at 70 °C overnight to afford a 0.63 mM solution of assembly $[Pd_2(\mathbf{LQ})_2(\mathbf{LC})_2](BF_4)_4$.

^{1}H NMR (700 MHz, acetonitrile-d_3) δ 9.87 (s, 2H, Ha), 9.47 (d, J = 1.9 Hz, 2H, H1), 9.26 (d, J = 6.8 Hz, 2H, Hb), 9.20 (dd, J = 6.1, 1.4 Hz, 2H, H2), 9.05 (d, J = 7.7 Hz, 2H, Hh´), 8.21 – 8.14 (m, 6H, H4, Hc, Hh´´), 8.12 (dt, J = 8.1, 0.9 Hz, 2H, Hd), 8.10 – 8.07 (m, 2H, Hg´´), 8.05 (dd, J = 8.0, 6.8 Hz, 2H, He), 8.01 (dd, J = 1.6, 0.7 Hz, 2H, H7), 7.89 (dd, J = 6.8, 1.0 Hz, 2H, Hf), 7.80 (dd, J = 8.6, 1.6 Hz, 2H, H5), 7.66 – 7.62 (m, 4H, H3, Hg´), 7.60 (d, J = 8.6 Hz, 2H, H6), 4.31 (t, J = 7.0 Hz, 2H, H8), 4.25 – 4.12 (m, 4H, Hi), *alkyl chains* Hj-Hn and H9-H151.77-1.70 (m, 2H), 1.44 – 1.34 (m, 2H), 1.34 – 1.06 (m, 14H), 0.81 – 0.68 (m, 5H), 0.66-0.58 (m, 2H), 0.37 – 0.24 (m, 2H), 0.21 – 0.10 (m, 2H), 0.07 – -0.14 (m, 8H).

^{13}C NMR (176 MHz, acetonitrile-d_3) δ 164.47 (C=O), 154.71 (Ca), 151.40 (C^q), 151.03 (C1), 150.63 (C2), 145.04 (C4), 142.39 (C^q), 142.25 (C^q),142.24 (Cb), 140.65 (C^q), 137.86 (C^q), 134.75 (Ce), 133.99 (C5), 132.96 (Cg'), 131.85 (Ch'), 131.60(Cg''), 130.29 (Cf), 129.97 (Ch''), 129.04 (C^q), 128.30(C3), 128.06 (Cd), 127.96 (C^q), 126.21 (Cc), 125.88 (C^q), 122.87 (C^q), 122.25 (C7), 113.07 (C^q), 111.89 (C6), 110.60 (C^q), 98.77 (C^q, **C**≡C-Py), 85.12 (C^q, C≡**C**-Py), 43.83 (C8), 43.75 (Ci), 32.29, 31.42, 29.89, 29.81, 29.71, 29.41, 27.53, 27.26, 23.16, 22.25,14.19, 13.61.

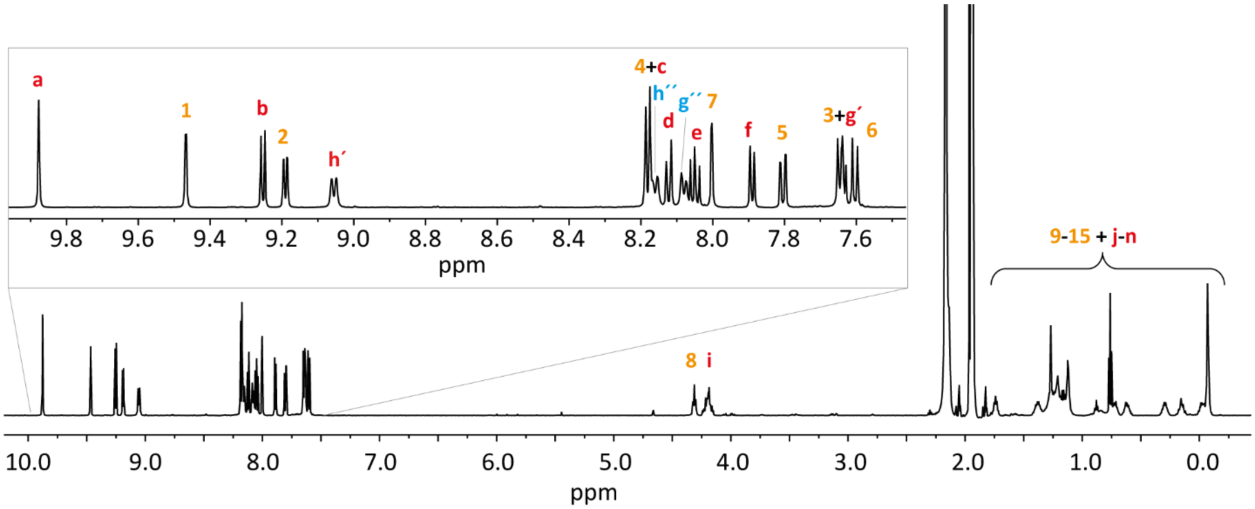

Figure S23: ^{1}H NMR spectrum (700 MHz, 298K, CD_3CN) of heteroleptic assembly $[Pd_2(LQ)_2(LC)_2](BF_4)_4$.

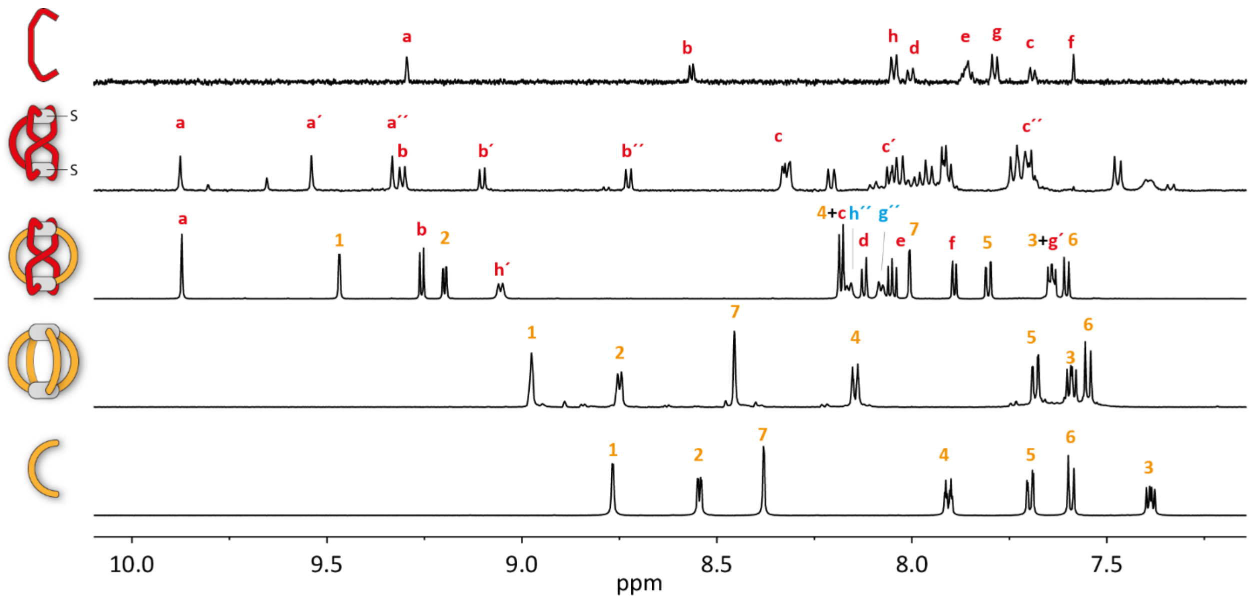

Figure S24: ^{1}H NMR stacked spectra in CD_3CN of, from the bottom, ligand LC, homoleptic cage $[Pd_2(LC)_4](BF_4)_4$, heteroleptic assembly $[Pd_2(LQ)_2(LC)_2](BF_4)_4$, homoleptic assembly $[Pd_2(LQ)_3(CH_3CN)_2](BF_4)_4$, ligand LQ.

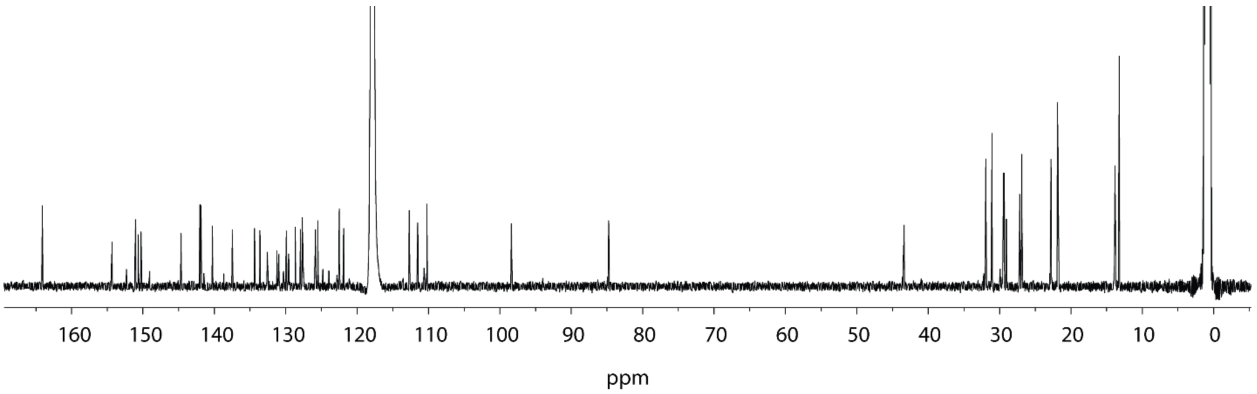

Figure S25: ^{13}C NMR spectrum (176 MHz, 298K, CD_3CN) of heteroleptic assembly $[Pd_2(LQ)_2(LC)_2](BF_4)_4$.

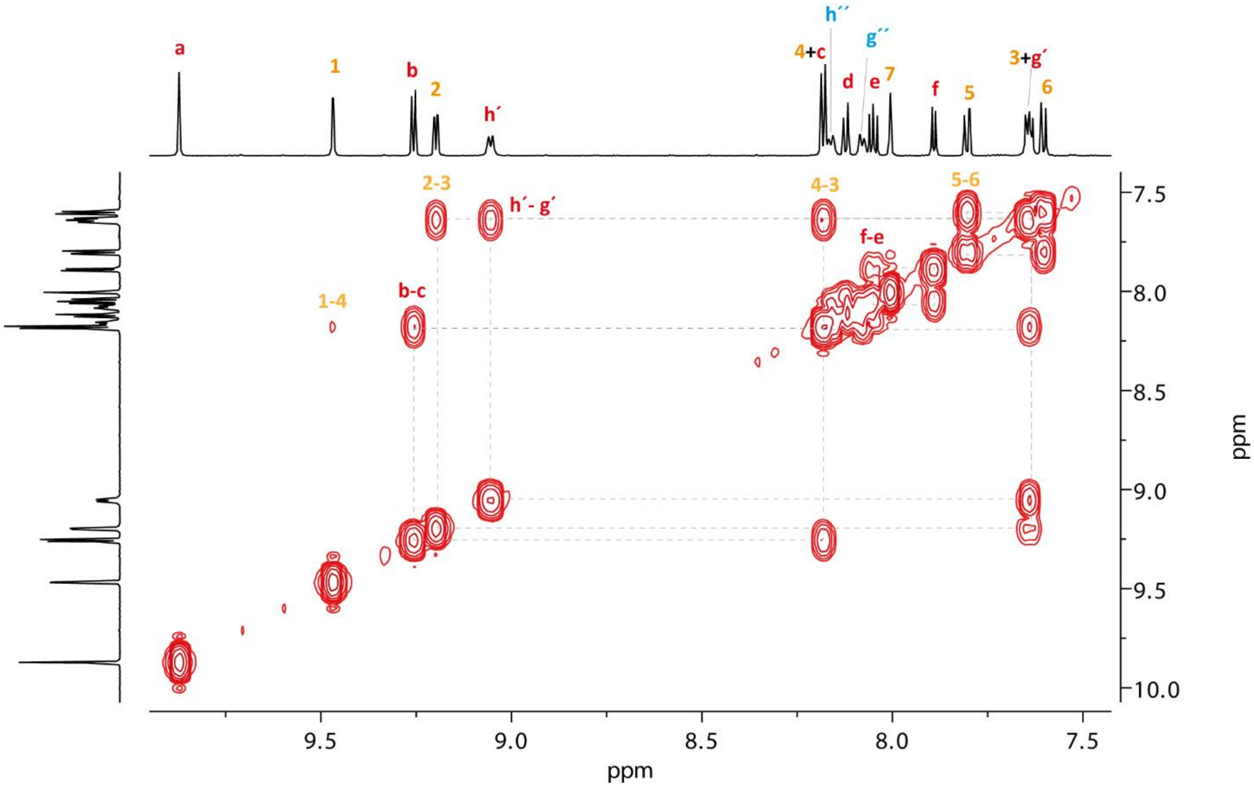

Figure S26: Enlargement of the aromatic region of the ^{1}H-^{1}H COSY (700 MHz, 298K, CD_3CN) spectrum of the heteroleptic assembly $[Pd_2(LQ)_2(LC)_2](BF_4)_4$.

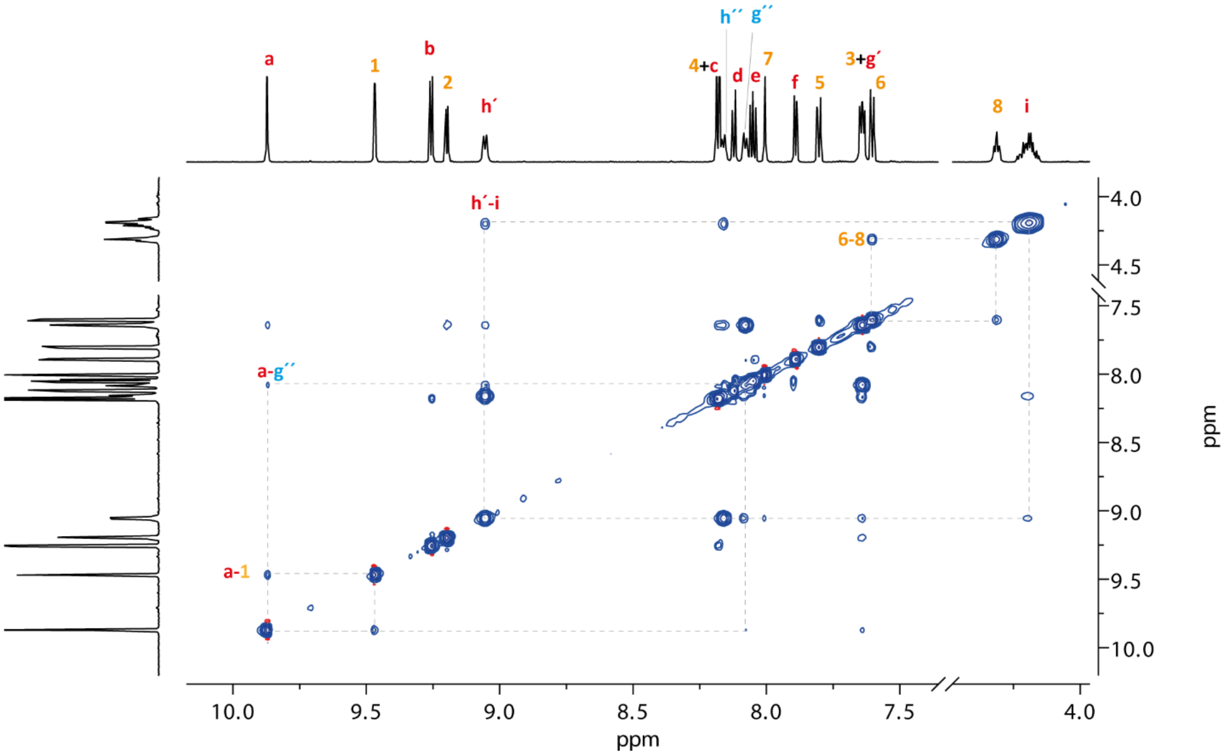

Figure S27: Enlargement of the aromatic region of the ^{1}H-^{1}H NOESY (700 MHz, 298K, CD_3CN) spectrum of the heteroleptic assembly $[Pd_2(LQ)_2(LC)_2](BF_4)_4$.

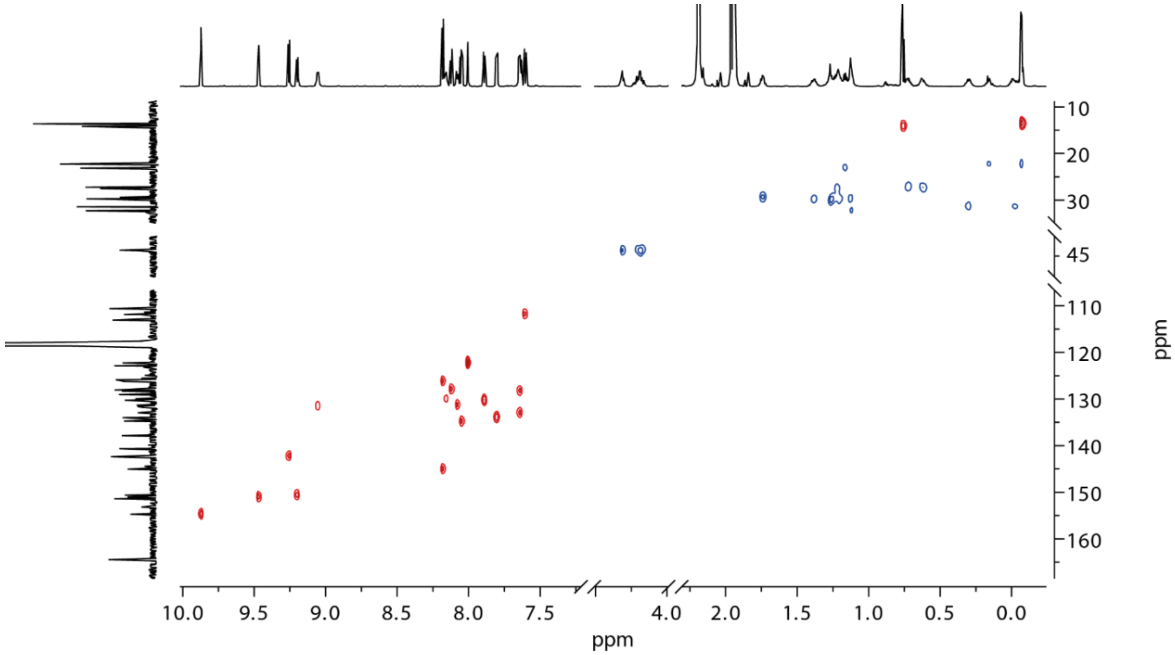

Figure S28: Enlargement of the aromatic region of the HSQC (700 MHz, 298K, CD_3CN) spectrum of the heteroleptic assembly $[Pd_2(LQ)_2(LC)_2](BF_4)_4$.

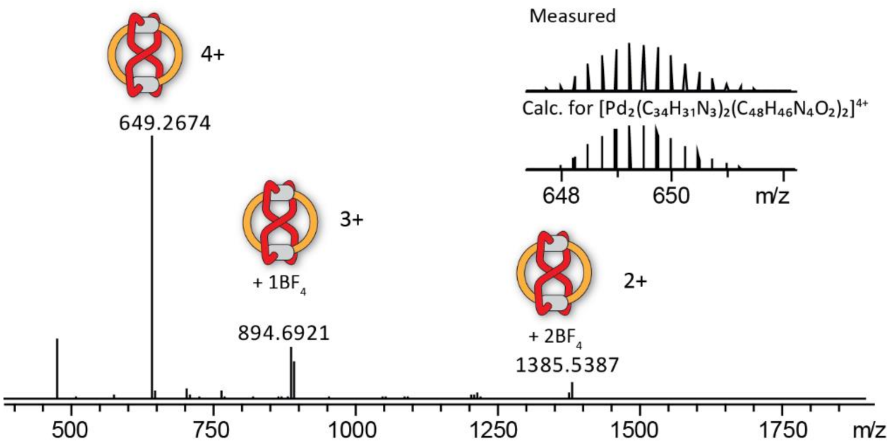

Figure S29: ESI-MS spectrum of $[Pd_2(LQ)_2(LC)_2+nBF_4]^{(4-n)+}$ with n=0-2. The observed and calculated isotopic patterns of $[Pd_2(LQ)_2(LC)_2]^{4+}$ are shown in the inset.

2.2.3 $[Pd_2($**LQ**$)_3(CD_3CN)_2](BF_4)_4$

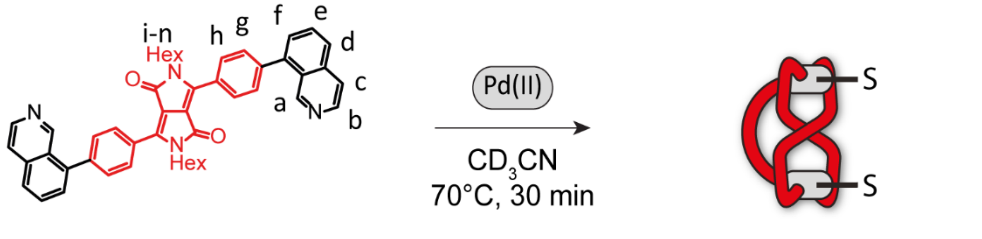

LQ

$[Pd_2($**LQ**$)_3(CH_3CN)_2](BF_4)_4$

Figure S30: Formation of assembly $[Pd_2(LQ)_3(CH_3CN)_2](BF_4)_4$ (S = solvent molecule).

A mixture of ligand **LQ** (450 µL of a 3.11 mM solution in CD_3CN) and $[Pd(CH_3CN)_4](BF_4)_2$ (75 µL of a 15 mM solution in CD_3CN) was equilibrated at room temperature for 6 h and then heated at 70 °C for 2 hours to afford a 0.89 mM solution of bowl $[Pd_2(\mathbf{LQ})_3(CH_3CN)_2](BF_4)_4$.

^{1}H NMR (600 MHz, acetonitrile-d_3) δ 9.91 (s, 1H, Ha), 9.58 – 9.55 (m, 1H, Ha'), 9.38 – 9.32 (m, 2H, Ha'', Hb), 9.13 (d, *J* = 6.7 Hz, 1H, Hb'), 8.76 (d, *J* = 6.7 Hz, 1H, Hb''), 8.38 – 8.33 (m, 3H, Hc), 8.26 – 8.21 (m, 1H), 8.15 – 8.01 (m, 4H, Hc'), 8.03 – 7.91 (m, 5H, Hc''), 7.79 – 7.69 (m, 7H), 7.53 – 7.48 (m, 2H), 7.42 (s, 2H), 5.51 (s, 0H), 4.01 – 3.95 (m, 1H), 3.83 – 3.72 (m, 1H), 3.72 – 3.65 (m, 0H), 3.60 (ddd, *J* = 14.7, 9.6, 4.9 Hz, 0H), 3.48 (t, *J* = 4.2 Hz, 1H), 2.84 – 2.62 (m, 2H), 2.18 (s, 118H), 2.15 (s, 6H), 1.76 – 1.70 (m, 1H), 1.53 (t, *J* = 7.2 Hz, 1H), 1.45 (s, 10H), 1.40 (d, *J* = 6.8 Hz, 1H), 1.34 (dt, *J* = 7.2, 2.9 Hz, 3H), 1.21 – 1.15 (m, 2H), 1.14 – 1.06 (m, 2H), 0.99 – 0.92 (m, 7H), 0.91 – 0.85 (m, 4H), 0.82 – 0.76 (m, 1H), 0.69 (t, *J* = 7.3 Hz, 3H). Partial assignment form ^{1}H-^{1}H COSY spectral analysis.

^{13}C NMR (151 MHz, acetonitrile-d_3) δ 162.94, 162.83, 162.81, 154.53, 154.40, 153.95, 149.08, 148.77, 148.39, 147.85, 143.76, 142.46, 142.24, 141.21, 141.18, 140.72, 140.52, 140.31, 139.95, 137.80, 137.61, 137.42, 134.53, 134.45, 132.56, 132.14, 131.72, 131.48, 131.25, 131.08, 130.99, 130.78, 130.37, 130.24, 128.97, 128.83, 128.67, 127.91, 127.67, 127.47, 126.00, 125.39, 111.17, 110.89, 109.89, 43.79, 43.42, 43.31, 31.85, 31.79, 31.58, 31.31, 30.97, 30.86, 29.90, 29.20, 28.97, 27.14, 27.03, 26.73, 26.53, 22.99, 22.93, 22.85, 22.70, 22.64, 14.01, 13.87, 13.78, 13.73.

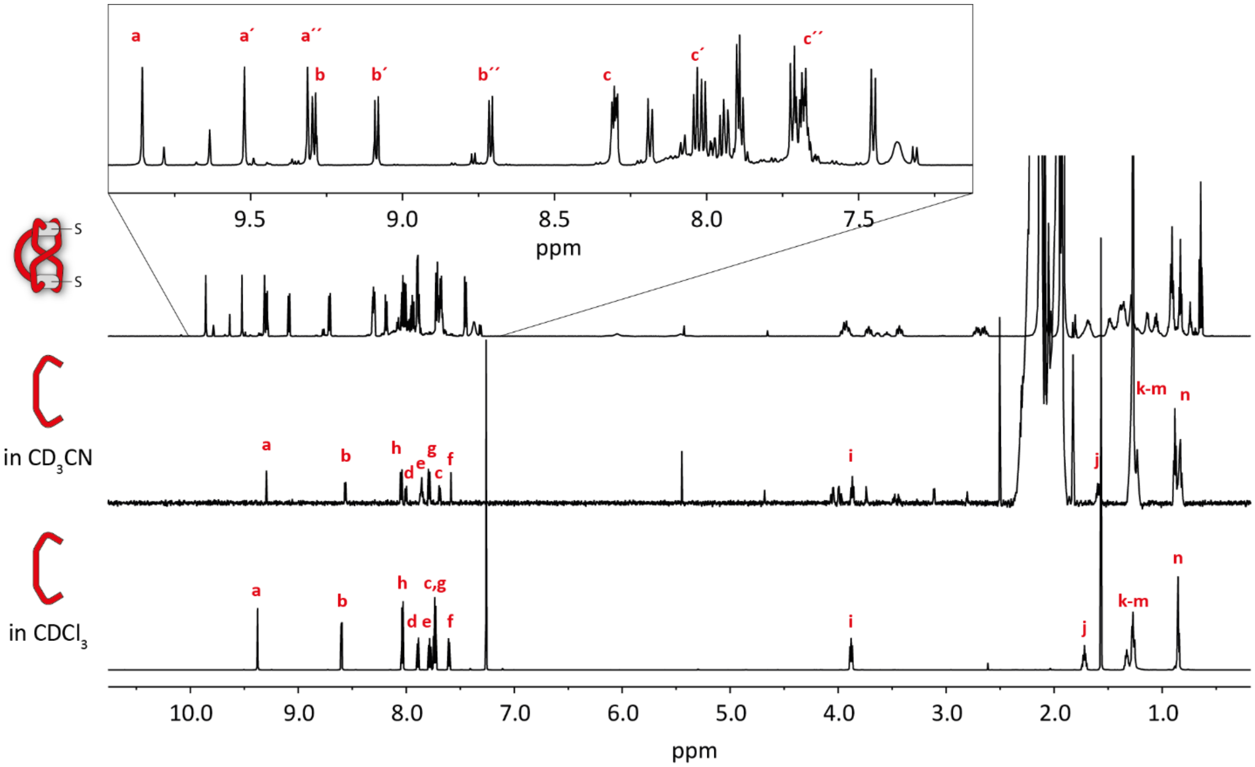

Figure S31: Stacked ^{1}H NMR spectra of ligand LQ in $CDCl_3$ (bottom), ligand LQ in CD_3CN (poorly soluble, middle) and ligand LQ in CD_3CN upon addition of 0.67 equiv. of Pd(II) and heating at 70 °C for 30 min (top).

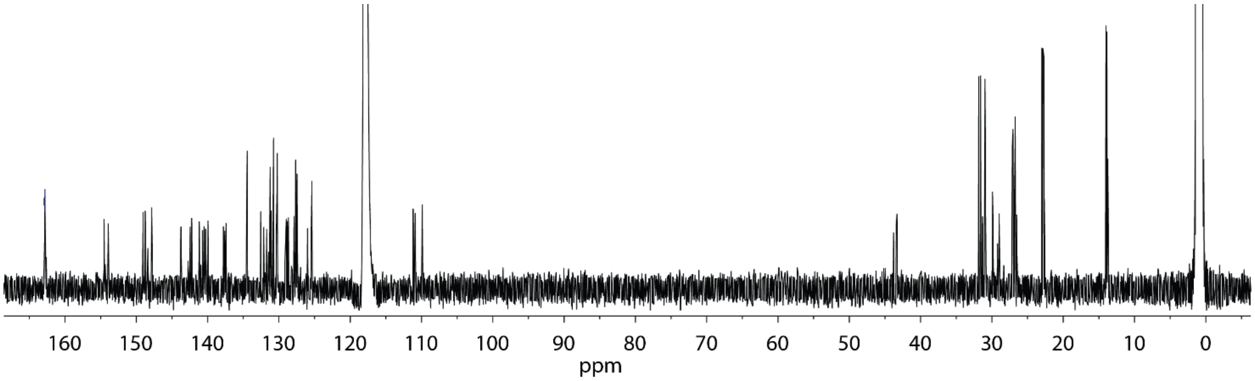

Figure S32: ^{13}C NMR spectrum (151 MHz, 298K, CD_3CN) of the assembly $[Pd_2(LQ)_3(CH_3CN)_2](BF_4)_4$.

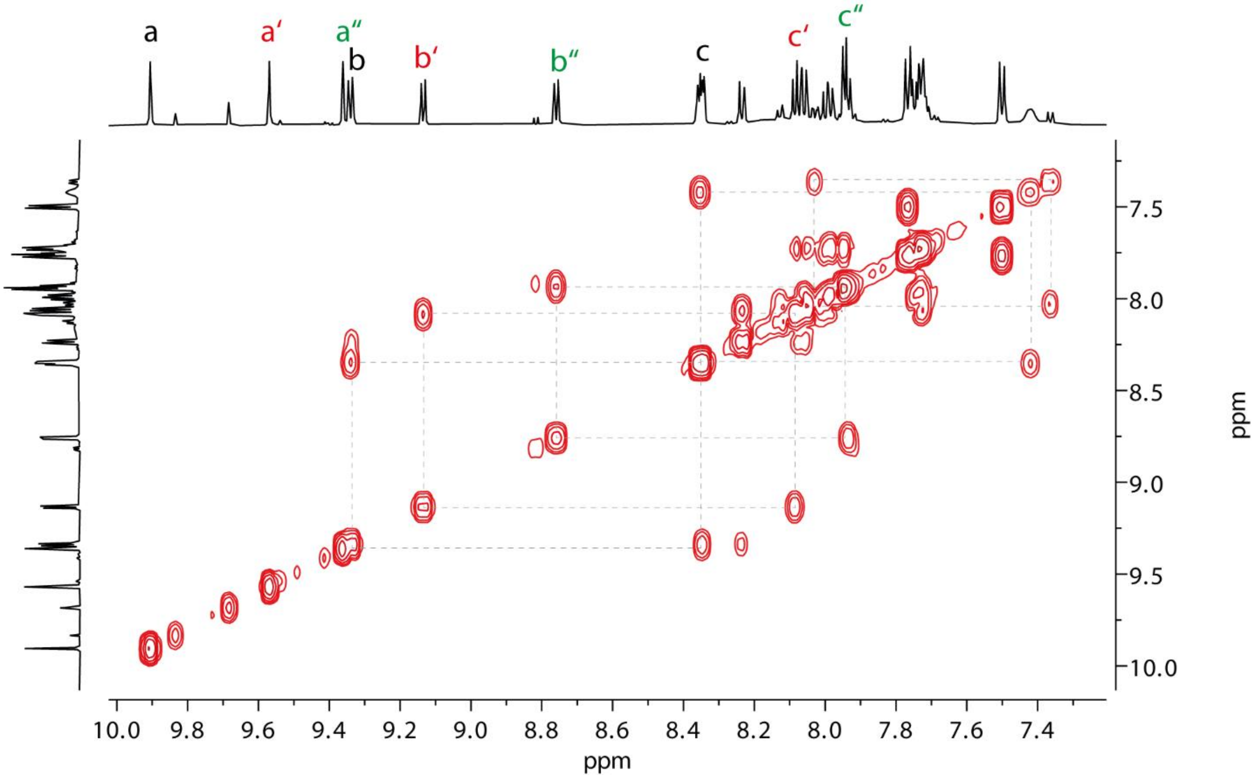

Figure S33: Enlargement of the aromatic region of the ^1H-^1H COSY (600 MHz, 298K, CD_3CN) spectrum of the assembly $[Pd_2(LQ)_3(CH_3CN)_2](BF_4)_4$ with partial assignment.

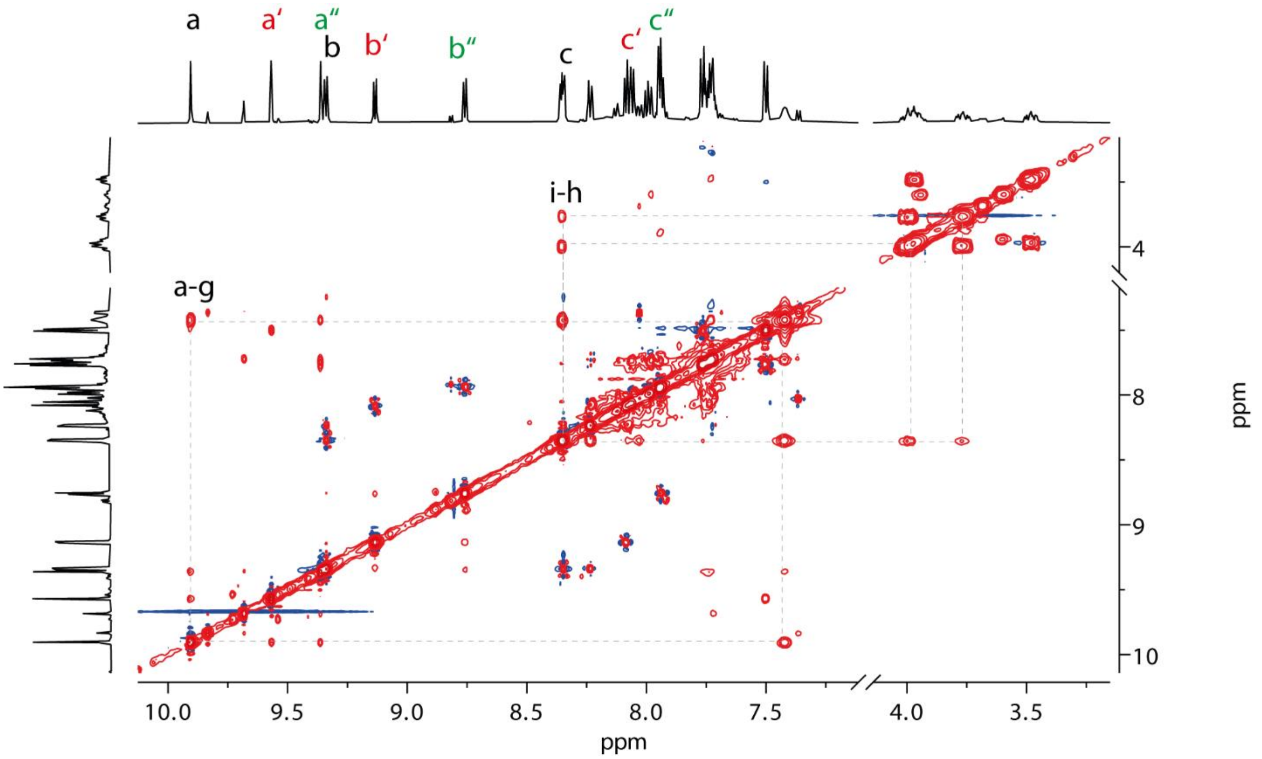

Figure S34: Enlargement of the aromatic region of the ^{1}H-^{1}H NOESY (600 MHz, 298K, CD_3CN) spectrum of the assembly $[Pd_2(LQ)_3(CH_3CN)_2](BF_4)_4$ with partial assignment.

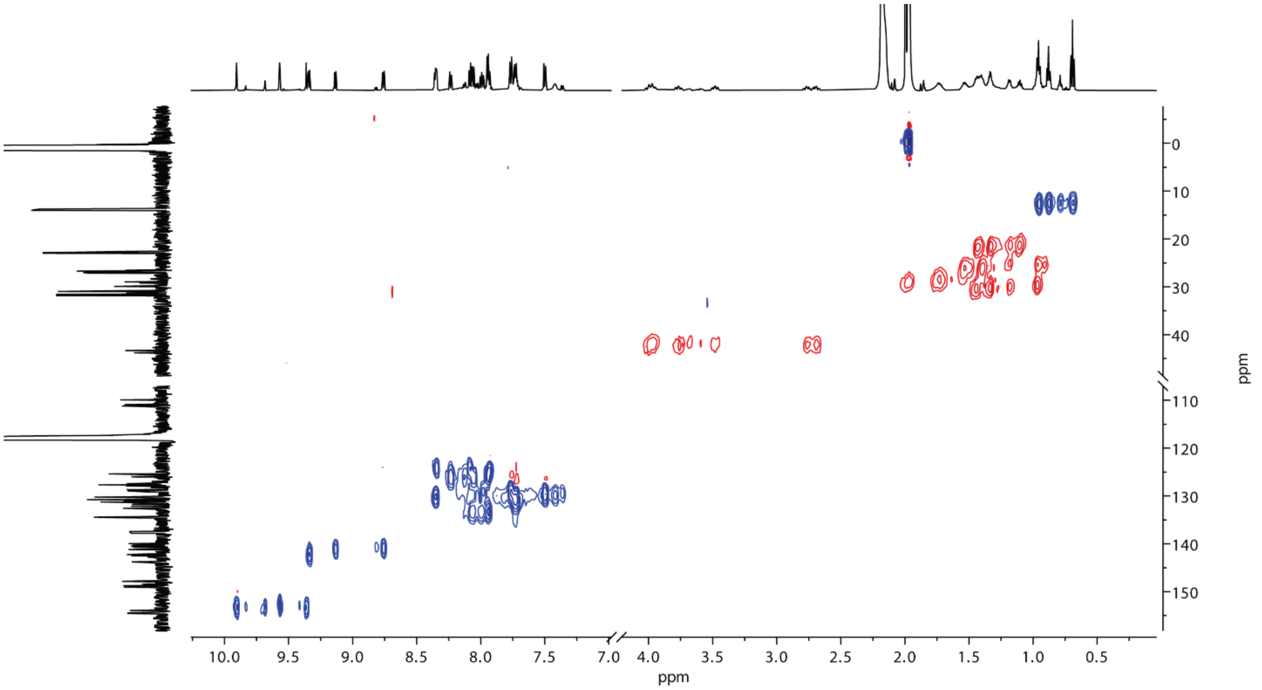

Figure S35: HSQC (600 MHz, 298K, CD_3CN) spectrum of the assembly $[Pd_2(LQ)_3(CH_3CN)_2](BF_4)_4$.

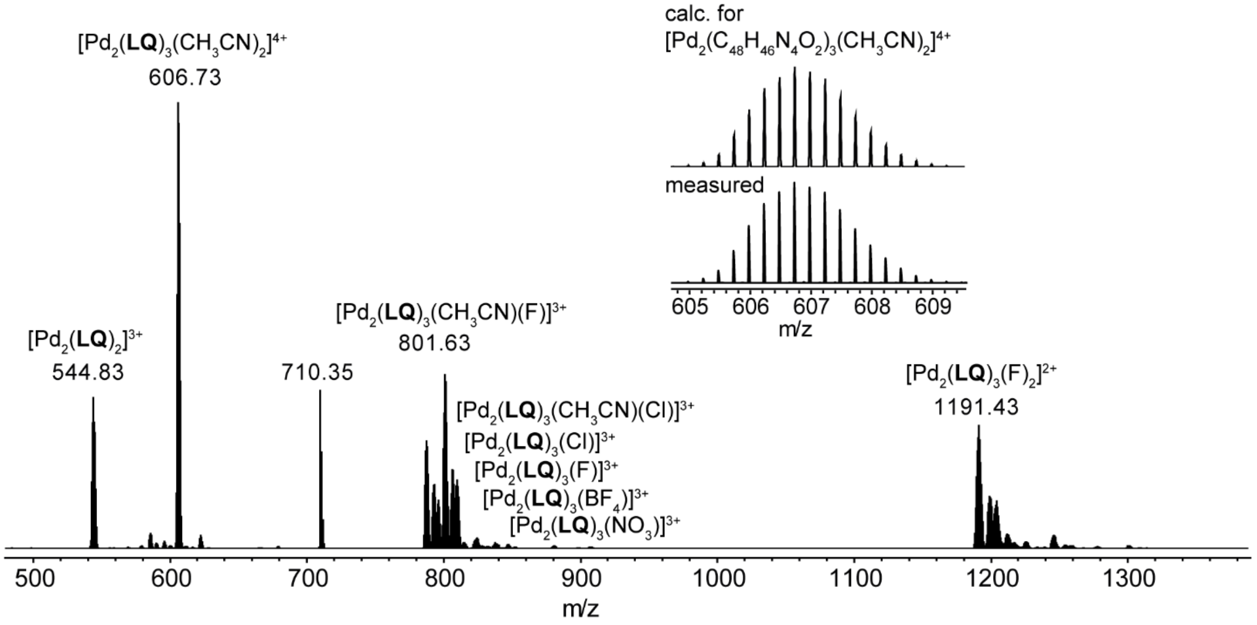

Figure S36: ESI-MS spectrum of $[Pd_2(LQ)_3(CH_3CN)_{0\text{-}2}{+}nX]^{4\text{-}n+}$ with X = F^-, BF_4^-, Cl^-, NO_3^- as anionic contaminants with measured and simulated patterns for $[Pd_2(LQ)_3(CH_3CN)_2]^{4+}$ shown in the inset. Species in which the solvent molecules are exchanged for various anions are assigned in the spectrum.

2.2.4 $[Pd_2(\mathbf{L3})_4](BF_4)_4$

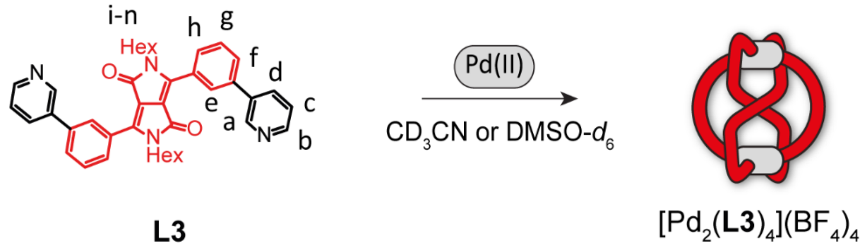

Figure S37: Formation of assembly $[Pd_2(L3)_4](BF_4)_4$.

A mixture of ligand **L3** (450 µL of a 3.11 mM solution) and $[Pd(CH_3CN)_4](BF_4)_2$ (50 µL of a 15 mM solution in the same solvent) was heated at 70 °C for 30 min to afford a 0.7 mM solution of assembly $[Pd_2(\mathbf{L3})_4](BF_4)_4$. The assembly was successfully obtained in DMSO-d_6, CD_3CN, acetone-d_6, THF-d_8, $MeNO_2$-d_3, DMF-d_7.

^{1}H NMR (600 MHz, acetonitrile-d_3) δ 10.66 (d, *J* = 2.1 Hz, 1H, Ha), 10.01 (d, *J* = 1.9 Hz, 1H, He), 9.98 (dd, *J* = 6.0, 1.3 Hz, 1H, Hb’), 9.61 (d, *J* = 2.0 Hz, 1H, Ha’), 8.73 (dd, *J* = 5.6, 1.2 Hz, 1H, Hb), 8.53 (dt, *J* = 8.1, 1.5 Hz, 1H, Hd), 8.38 (dt, *J* = 8.2, 1.6 Hz, 1H, Hd’), 8.03 (dd, *J* = 7.7, 1.9 Hz, 1H, Hf), 7.96 (dd, *J* = 8.1, 6.0 Hz, 1H, Hc’), 7.83 – 7.70 (m, 3H, Hf’, Hc, Hh’), 7.67 (t, *J* = 7.8 Hz, 1H, Hg), 7.58 (t, *J* = 7.7 Hz, 1H, Hg’), 7.40-7.36 (m, He’), 7.34 – 7.24 (m, 1H, Hh), 2.91 (ddd, *J* = 14.8, 12.6, 5.5 Hz, 1H), 2.66 (ddd, *J* = 14.4, 8.2, 6.0 Hz, 1H), 2.57 (ddd, *J* = 15.0, 8.5, 7.0 Hz, 1H), 2.39 (ddd, *J* = 14.8, 12.6, 4.3 Hz, 1H), 0.86 (qd, *J* = 8.4, 7.1, 4.2 Hz, 2H), 0.74 (dd, *J* = 10.9, 4.0 Hz, 2H), 0.70 – 0.57 (m, 6H), 0.53 (t, *J* = 7.4 Hz, 3H), 0.41 (dq, *J* = 13.0, 8.8, 7.7 Hz, 3H), 0.29 (q, *J* = 7.4, 6.7 Hz, 4H), 0.21 – -0.01 (m, 2H).

^{1}H NMR (600 MHz, dimethyl sulfoxide-d_6) δ 10.64 (d, *J* = 2.0 Hz, 1H, Ha), 10.53 – 10.29 (m, 1H, Hb’), 9.98 (s, 1H, He), 9.55 (d, *J* = 1.9 Hz, 1H, Ha’), 8.96 (d, *J* = 5.5 Hz, 1H, Hb), 8.87 (d, *J* = 8.0 Hz, 1H, Hd), 8.68 (d, *J* = 8.1 Hz, 1H, Hd’), 8.48 – 8.26 (m, 1H, Hf), 8.20 (dd, *J* = 8.1, 6.0 Hz, 1H, Hc’), 7.97 (dd, *J* = 8.0, 5.5 Hz,

1H, Hc), 7.89 (d, *J* = 7.8 Hz, 1H, Hf'), 7.85 – 7.72 (m, 2H, Hg, Hh'), 7.65 (t, *J* = 7.7 Hz, 1H, Hg'), 7.36 (s, 1H, He'), 7.22 (d, *J* = 8.0 Hz, 1H, Hh), 2.90 (q, *J* = 11.0, 9.4 Hz, 1H), 2.78 – 2.58 (m, 1H), 2.42 – 2.23 (m, 2H), 0.84 (d, *J* = 16.8 Hz, 1H), 0.77 – 0.55 (m, 6H), 0.55 – 0.42 (m, 4H), 0.36 (q, *J* = 7.4, 5.8 Hz, 5H), 0.28 – 0.16 (m, 1H), 0.14 (t, *J* = 7.3 Hz, 3H), 0.10 – -0.15 (m, 2H).

^{13}C NMR (151 MHz, acetonitrile-d_3) δ 163.97, 161.49, 151.76, 151.45, 148.94, 148.40, 147.36, 141.31, 140.41, 139.15, 139.04, 135.29, 134.66, 132.75, 131.61, 131.55, 131.00, 130.38, 129.90, 129.62, 129.38, 129.20, 128.74, 127.15, 111.51, 110.12, 44.96, 40.23, 31.40, 31.04, 28.71, 27.99, 26.66, 26.23, 23.01, 22.82, 14.10, 13.97 (2 C overlapping).

^{13}C NMR (151 MHz, dimethyl sulfoxide-d_6) δ 171.42, 162.41, 159.89, 151.67, 150.69, 146.88, 146.02, 139.49, 139.18, 138.21, 137.12, 133.88, 133.33, 131.44, 130.81, 130.15, 129.03, 128.84, 127.96, 127.76, 127.67, 127.54, 125.20,118.09, 109.87, 108.31, 43.45, 29.99, 29.36, 27.23, 26.36, 25.33, 24.95, 21.62, 21.42,13.54,13.32.

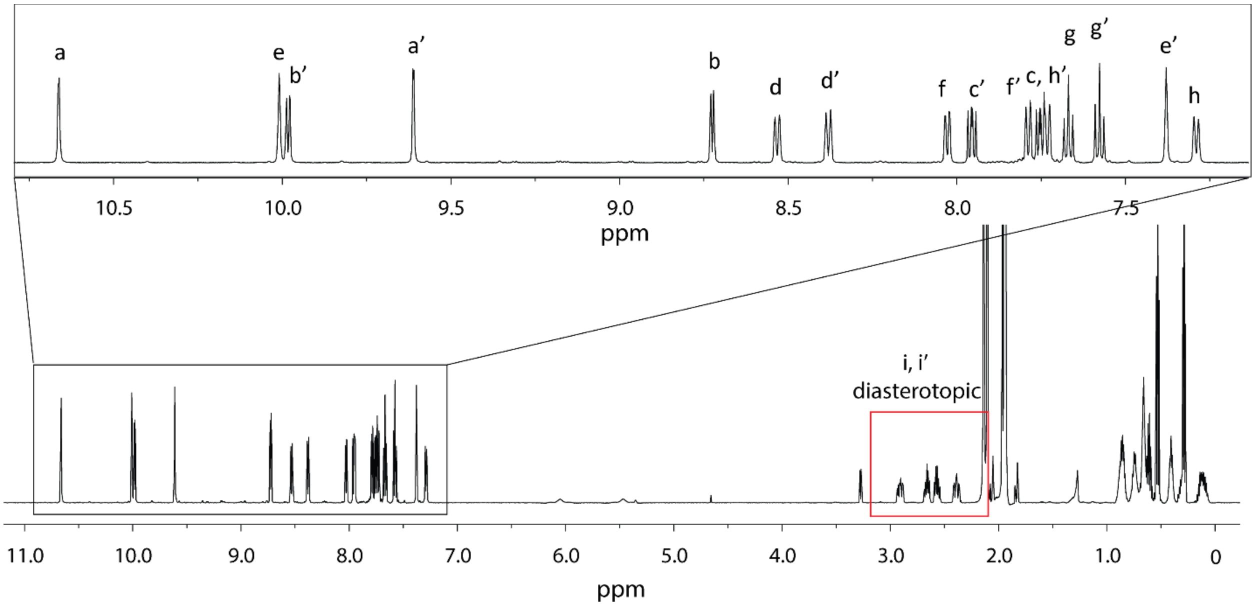

Figure S38: ^{1}H NMR spectrum (600 MHz, 298K, CD_3CN) of $[Pd_2(L3)_4](BF_4)_4$.

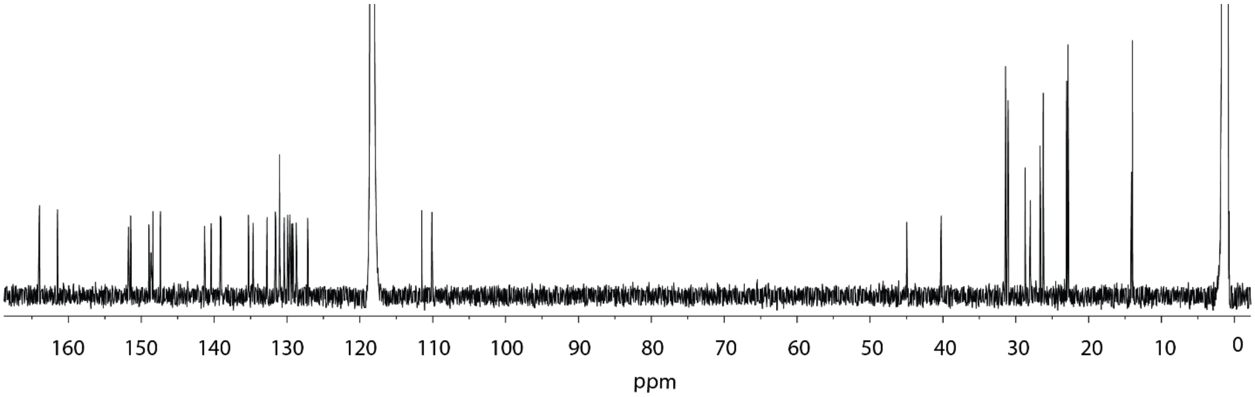

Figure S39: ^{13}C NMR spectrum (151 MHz, 298K, CD_3CN) of $[Pd_2(L3)_4](BF_4)_4$.

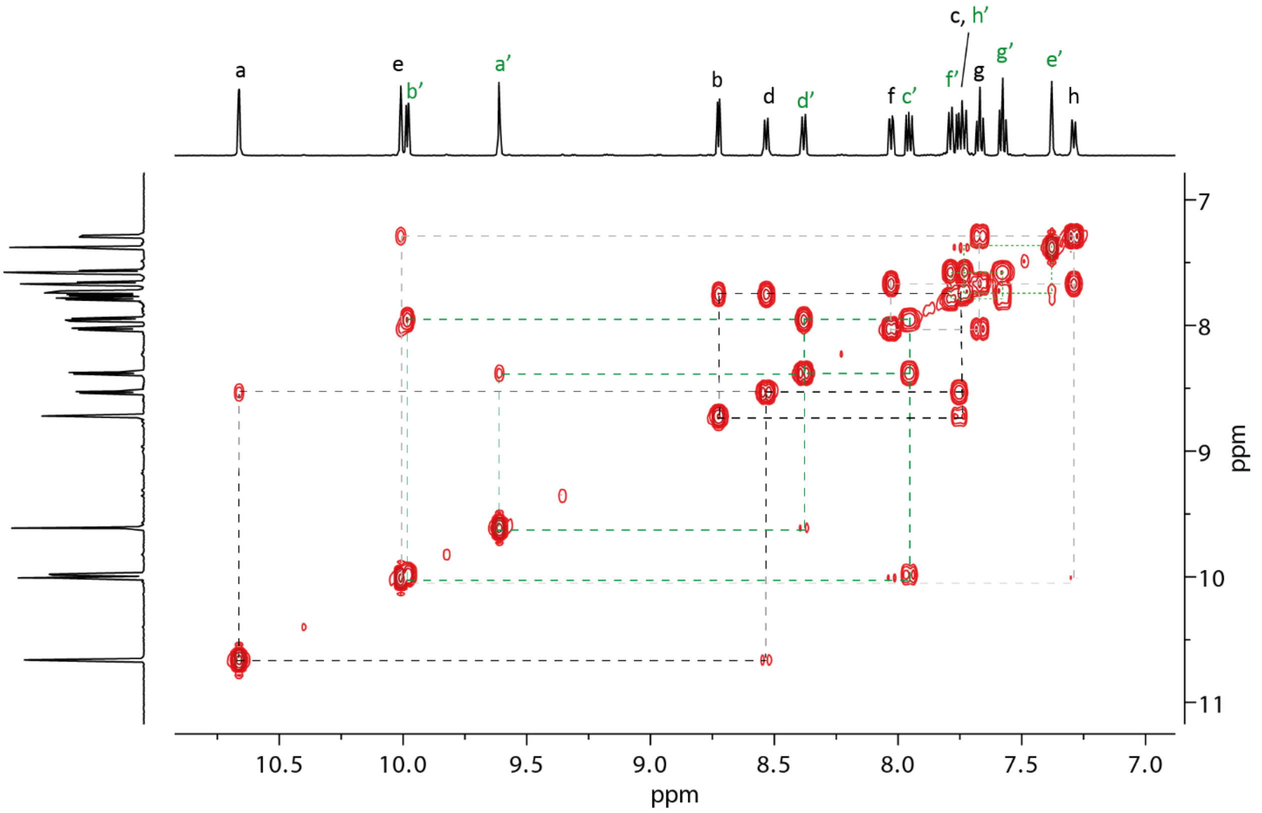

Figure S40: Enlargement of the aromatic spectral region of ^{1}H-^{1}H COSY NMR spectrum (600 MHz, 298K, CD_3CN) of the assembly $[Pd_2(L3)_4](BF_4)_4$.

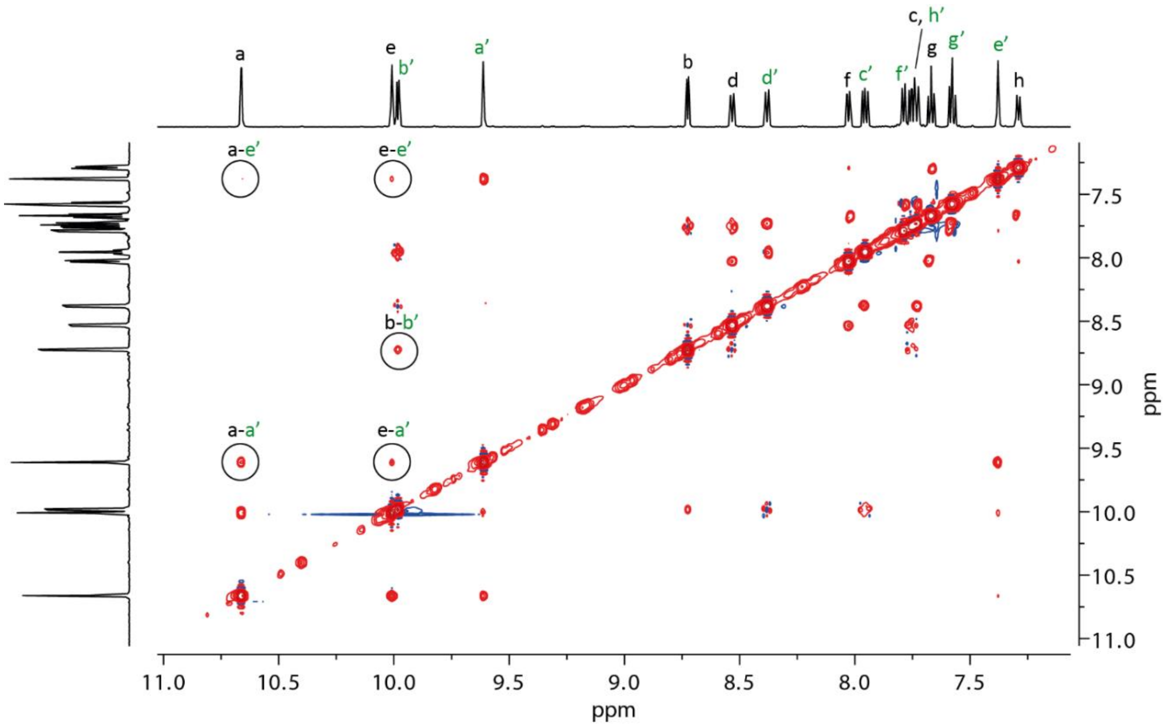

Figure S41: Enlargement of the aromatic spectral region of ^{1}H-^{1}H NOESY NMR spectrum (600 MHz, 298K, CD_3CN) of the assembly $[Pd_2(L3)_4](BF_4)_4$.

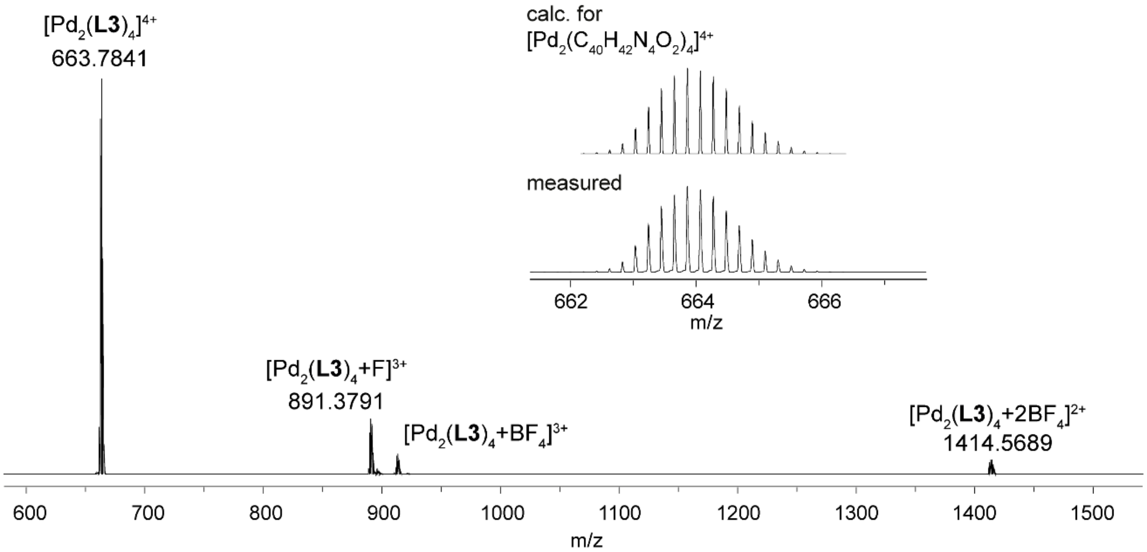

Figure S42: ESI-MS spectrum of $[Pd_2(L3)_4+nBF_4]^{(4-n)+}$ with n=0-2. The observed and calculated isotopic patterns of $[Pd_2(L3)_4]^{4+}$ are shown in the inset.

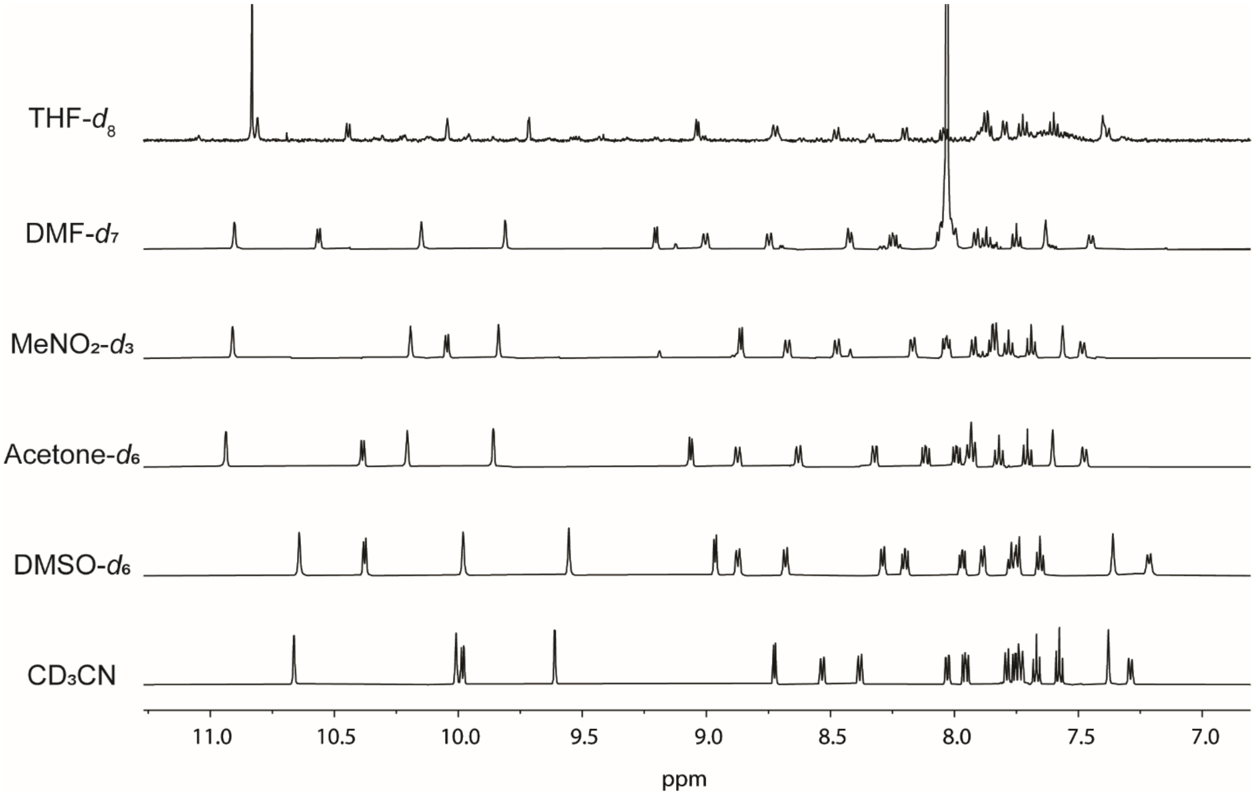

Figure S43: Stacked ^{1}H NMR spectra of cage $[Pd_2(L3)_4](BF_4)_4$ in the different indicated solvents. The same pattern of two sets of equally integrated signals for the ligand L3 are found, thus indicating the formation of the same topology upon Pd(II) addition.

3. Optical Properties and Spectral Characterization

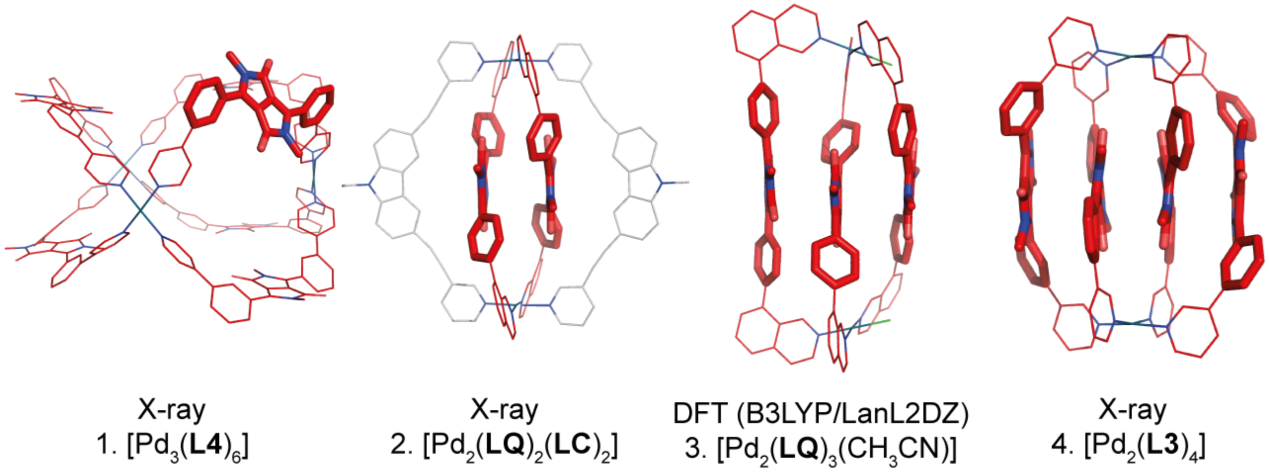

Figure S44: Comparison of the structures of $Pd_3(L4)_6$ (X-ray), $Pd_2(LQ)_2(LC)_2$ (X-ray), $Pd_2(LQ)_3(Cl)_2$ (DFT) and $Pd_2(L3)_4$ (X-ray) with DPP stacking highlighted by thicker sticks.

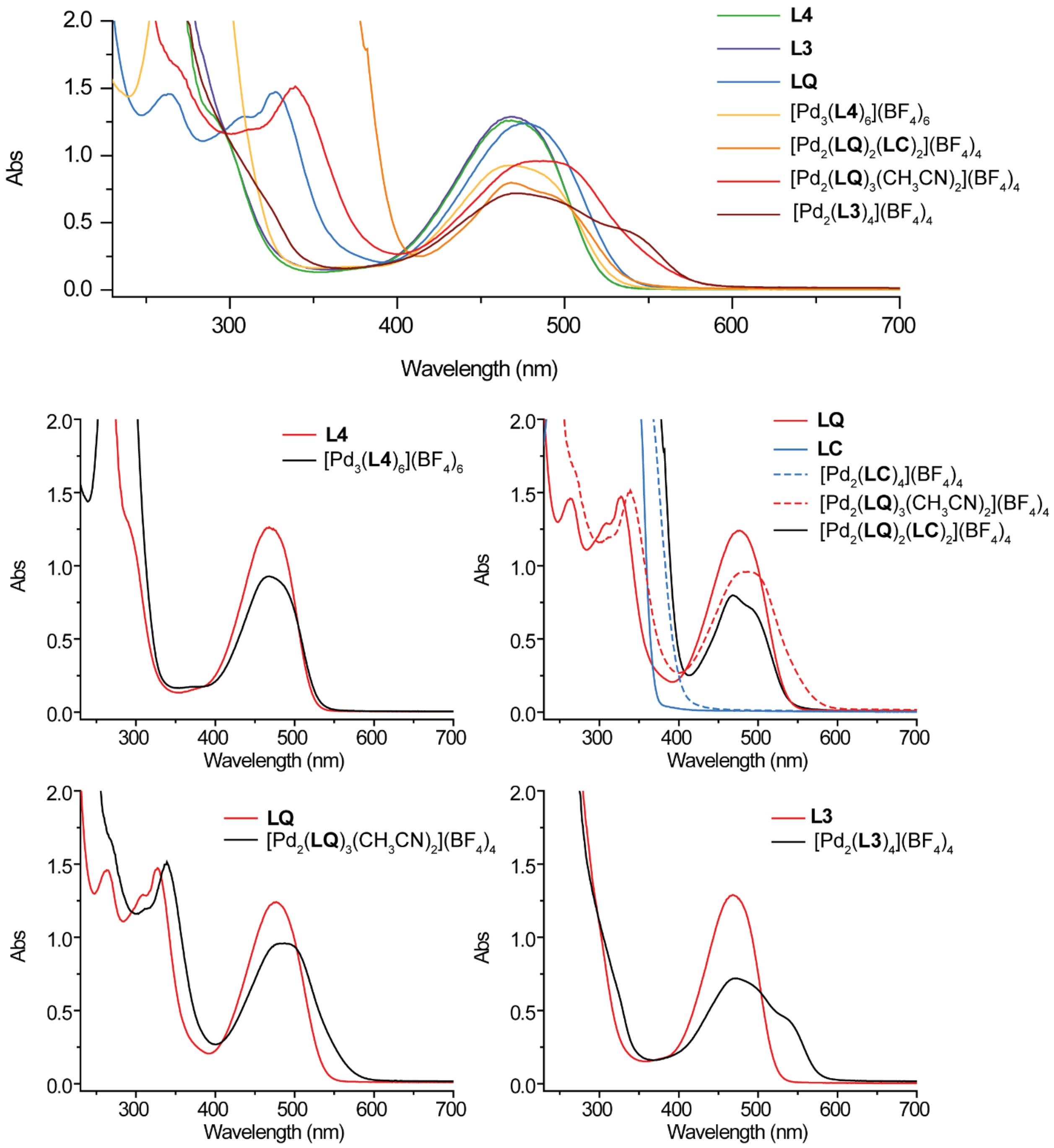

Figure S45: Absorption spectra in acetonitrile of ligands L4, L3, LQ, LC and corresponding homoleptic or heteroleptic assemblies upon addition of the required amount of Pd(II) as explicitly indicated in the figure legends. The same concentration of chromophores, 0.31 mM, was used for all the samples in cuvettes with an optical path of 0.2 cm.

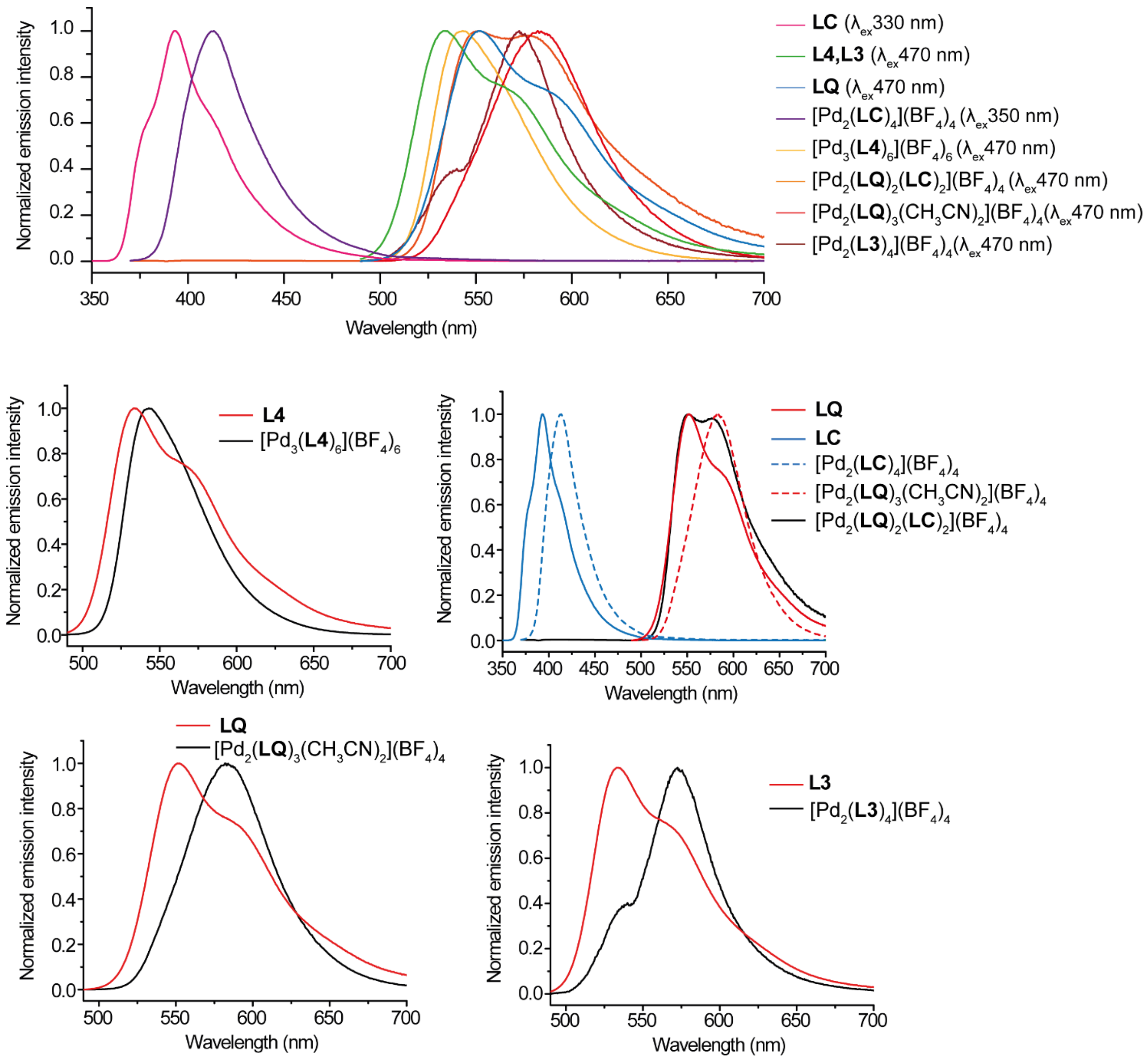

Figure S46: Emission spectra in acetonitrile of the ligands L4, L3, LQ, LC and corresponding homoleptic or heteroleptic assemblies upon addition of the required amount of Pd(II) as explicitly indicated in the figure legends. The same concentration of chromophores, 0.31 mM, was used for all the samples in cuvettes with an optical path of 0.2 cm.

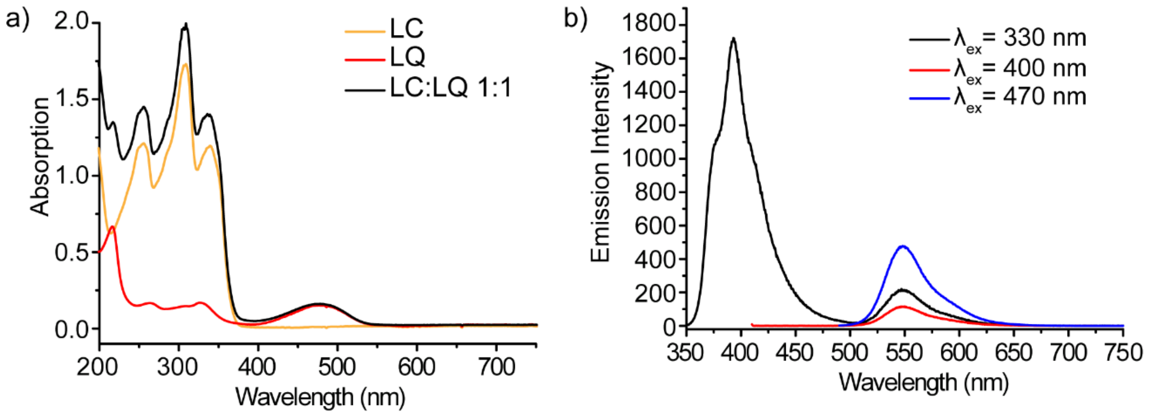

Figure S47: a) absorption spectra in acetonitrile of ligand **LC** (yellow), ligand **LQ** (red) and a 1:1 mixture of ligand **LC** and **LQ** (black); b) emission spectra of a 1:1 mixture of **LQ** and **LC** at different excitation wavelength as indicated in the legend. As it is possible to observe from the Figure, when the two chromophore-based ligands are present in solution without Pd(II) cations, the emission of **LC** is highly predominant in contrast to what it is observed when they are in the assembly (Figure S46) where the emission of **LC** is quenched. This supports that it is only in the assembly that the two chromophores are in sufficient proximity for energy transfer to happen. The same concentration of chromophores of 10 µM was used for all the samples in cuvettes with an optical path of 1 cm.

3.1 Photoluminescence Quantum Efficiency (PLQE) Measurements

The steady-state Photoluminescence Quantum Yield (PLQY) measurements were performed using an integrating sphere (Labsphere 3P-GPS-060-SF) following the Greenham-DeMello method.[5,6] Continuous-wave excitation is provided by a 405 nm diode laser with excitation powers of 0.001-3.2 mW/cm^2. A focussed beam of diameter 500 µm was used to excite the samples. The emission was directed using an optical fiber in a calibrated grating spectrometer (Andor Shamrock 500i) onto a Si-Camera (Andor iDus DU420A).

Table S1: Photoluminescence quantum efficiencies (PLQE) for assemblies and ligands.[a]

Label	Composition	Individual PLQE values recorded (%)	Laser fluence of measurements (µJcm^{-2})	PQLE(%)
1	$[Pd_3(\mathbf{L4})_6](BF_4)_6$ (+ higher order species)	19.9, 21.1, 19.3, 23.9, 20.9	10.0, 8.6, 10.2, 10.0, 8.0<br><br>1000µm effective beam diameter	21.0 ± 1.6
2	$[Pd_2(\mathbf{LQ})_2(\mathbf{LC})_2](BF_4)_4$	49.1, 54.0, 51.1, 52.0, 50.1		51.3 ± 1.7
3	$[Pd_2(\mathbf{LQ})_3(CH_3CN)_2](BF_4)_4$	2.4, 3.1, 3.4, 2.8, 3.3		3.0 ± 0.4
4	$[Pd_2(\mathbf{L3})_4](BF_4)_4$	0.8, 0.7, 0.7, 0.8, 0.6		0.72 ± 0.07
5	**L4**	94.8, 93.9, 93.5, 94.1, 94.4		94.1 ± 0.4

6	**L3**	93.5, 94.4, 90.1, 92.7, 93.8		92.9 ± 1.5
7	**LQ**	96.4, 98.1, 96.8, 93.00, 96.1		96.1 ± 1.7

[a] PLQE of **LC** could not be determined using our integrating sphere setup, as the excitation wavelength (350 nm) that would need to be recorded using the Greenham-DeMello[5,6] method is much lower than the lowest wavelength possible to be recorded using our spectrometer, whose most appropriate grating is blazed at 600 nm, allowing for data recording down to 375 nm only.

In Figure S48 the photoluminescence spectra (1 kHz repetition rate, 100 fs long 400 nm excitation pulses, 20 uW) of the assemblies compared to the respective ligands are reported.

a) $[Pd_3(\mathbf{L4})_6](BF_4)_6$ shows a red shifted emission compared to **L4**. Moreover, **L4** shows a much longer-lived emission compared to the assembly. Both observations are evidence for genuine complex emission.

b) $[Pd_2(\mathbf{LQ})_2(\mathbf{LC})_2](BF_4)_4$ has a slight red shift in the emission, but here the complex has the longest emission.

c) The emission of $[Pd_2(\mathbf{LQ})_3(CH_3CN)_2](BF_4)_4$ is highly quenched and has pronounced red features, which could be indicative of excimer coupling between the stacked ligands or formation of a charge transfer (CT) state. In this case, the decay of the emission is very fast, much faster than for the ligand. See comparison between the traces at 4 ns and 8 ns in Figure S48.

d) Also the emission of $[Pd_2(\mathbf{L3})_4](BF_4)_4$ is highly quenched and has pronounced red features, which could be indicative of excimer coupling between the stacked ligands or formation of a charge transfer (CT) state. Again, the decay of the emission is very fast, much faster than for the ligand. See comparison between the traces at 4 ns and 8 ns in Figure S48.

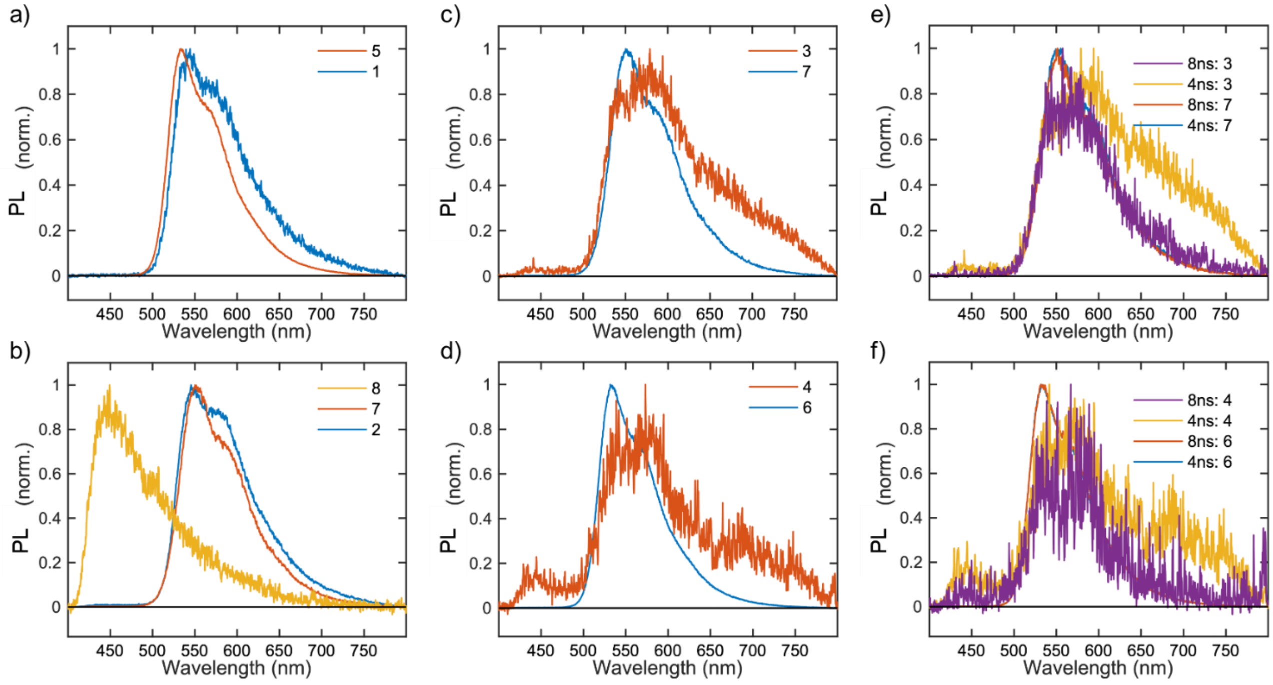

Figure S48: Photoluminescence spectra (1 kHz repetition rate, 100 fs long 400 nm excitation pulses, 20 µW), 4-16 ns integrated.

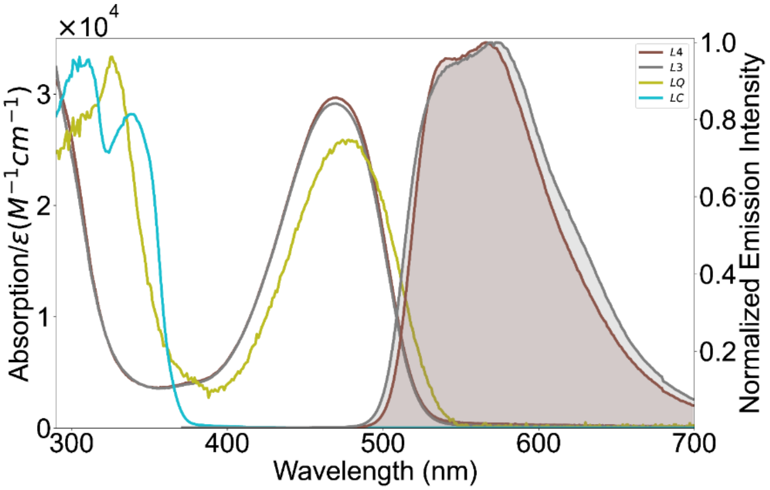

Figure S49: Steady state absorption and continuous wave photoluminescence (PL, filled) spectra of the ligands under 405 nm excitation at 5 µJ/cm^2 fluence.

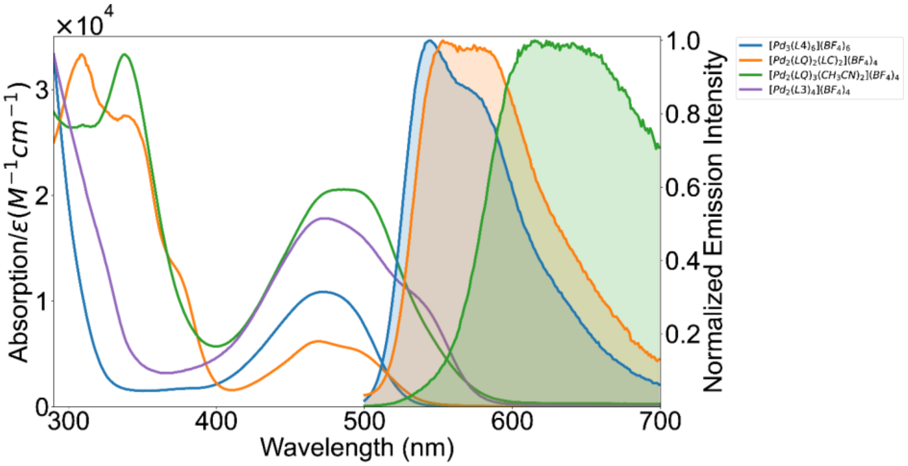

Figure S50: Steady state absorption and continuous wave photoluminescence (PL, filled) spectra of the cages studied in main text under 405 nm excitation at a fluence of 5 µJ/cm^2.

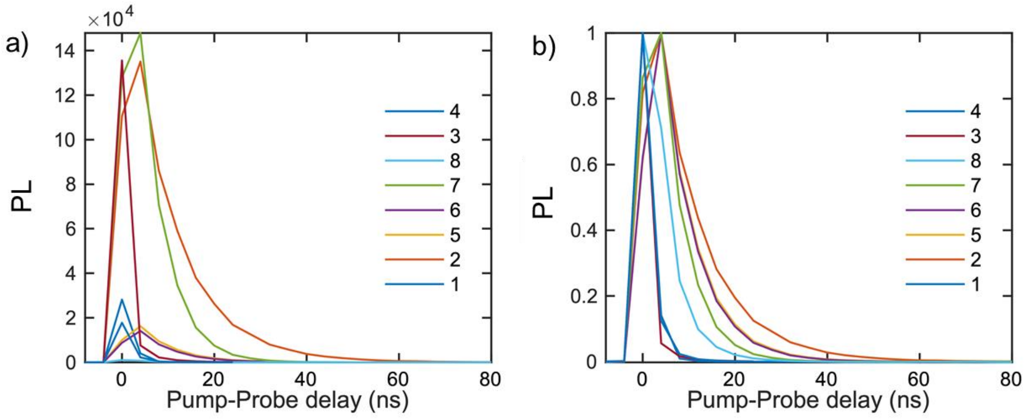

Figure S51: Kinetics of emission, excited at 400 nm pulsed excitation (repetition rate 1 kHz, pulse duration ca. 100 fs).

In order to rule out any partial decomposition of the heteroleptic cage under release of free **LQ** ligand, we performed our measurements on solutions containing an excess of Pd(II) cations. This led to no noticeable changes in the PLQE. If there were free ligands in solution that were responsible for the PLQE we would see a quenching of the PLQE directly upon adding more Pd(II) cations. The absence of this behavior demands our attention on the fact that (a) The solution contains no free ligand and (b) The metal-organic assembly is chemically stable. Furthermore, we performed fluence dependent studies on Pd_2(**LQ**)$_2$(**LC**)$_2$ and **LQ** as shown below in Table S2 and Figure S52.

Table S2: Fluence dependent PLQE measurements of solutions of the ligand LQ and $Pd_2(LQ)_2(LC)_2$.

Name	PLQE at 10µJcm^{-2} excitation fluence (%)	PLQE at 20µJcm^{-2} excitation fluence (%)	PLQE at 30µJcm^{-2} excitation fluence (%)	PLQE at 35µJcm^{-2} excitation fluence (%)
**LQ**	97.0	93.00	84.0	80.0
[Pd_2(**LQ**)$_2$(**LC**)$_2$](BF_4)$_4$	52.0	50.0	52.0	51.0

From this data we find that the PLQE of Pd_2(**LQ**)$_2$(**LC**)$_2$ is significantly less fluence dependent than that of ligand **LQ**. This shows that Pd_2(**LQ**)$_2$(**LC**)$_2$ is more resistant to photobleaching than the constituent high-PLQE ligand **LQ** , whose sharp decline in PLQE with increasing fluence most likely arises from multi-exciton annihilation or photo-degradation. The absence of such a trend for the cage solution further supports the persistence and stability of the cage as the main species measured connected to the PLQE stated for it.

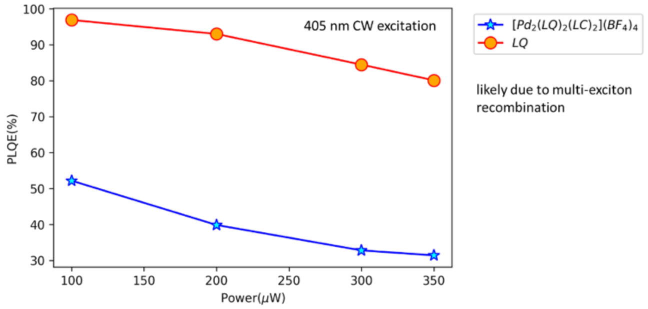

Figure S52: PLQE fluence dependence for LQ and $Pd_2(LQ)_2(LC)_2$, excited using a 405 nm CW laser with an effective beam diameter of approx. 1.0 mm.

4. X-ray crystal structure analysis

Three different supramolecular assemblies were studied using single-crystal X-ray crystallography. In total, we determined five different structures, one crystal form of the Pd_3(**L4**)$_6$ ring; two different polymorphs of heteroleptic Pd_2(**LQ**)$_2$(**LC**)$_2$ from either needle- and block-shaped crystals originating from the same crystallization conditions as well as two different solvates (from diffusion of diethyl and methyl-t-butyl as counter solvent) of homoleptic Pd_2(**L3**)$_4$. The crystals of the supramolecular assemblies were extremely sensitive to loss of organic solvent. Due to very thin plate- or needle-shaped crystals, the analysis was further hampered by the limited scattering power of the samples, only allowing in one case to reach the desired atomic resolution using an in-house microfocussed X-ray CuK_{α} source. In the other four cases, gaining detailed structural insight required cryogenic crystal handling and highly brilliant synchrotron radiation. Hence, diffraction data of most supramolecular assemblies was collected during two beamtime shifts at macromolecular synchrotron beamline P11, PETRA III, at DESY, Hamburg.[7] Counterion and solvent molecule disorder required carefully adapted macromolecular refinement protocols employing geometrical restraint dictionaries, similarity restraints and restraints for anisotropic displacement parameters (ADPs). Details are found in the discussions below.

Table S3: Crystal data and structure refinement for supramolecular assemblies

**Compound**	Pd_3(**L4**)$_6$	Pd_2(**LQ**)$_2$(**LC**)$_2$	Pd_2(**LQ**)$_2$(**LC**)$_2$
**CIF ID**	**ir12a_sq**	**ir36_needle_sq**	**ir36_block_sq**
**CCDC no**	**2168202**	**2168203**	**2168204**
Empirical formula	$C_{240}H_{250}B_5F_{20}N_{24}O_{12}Pd_3$	$C_{157.12}H_{144.25}B_{2.12}F_{8.50}N_{14}O_6Pd_2$	$C_{144}H_{114}B_3F_{12}N_{14}O_4Pd_2$
Formula weight	4415.88	2721.88	2577.72
Temperature [K]	80(2)	100(2)	100(2)
Crystal system	triclinic	triclinic	orthorhombic
Space group (number)	$P\bar{1}$ (2)	$P\bar{1}$ (2)	$Pbcn$ (60)
a [Å]	18.590(4)	34.226(18)	27.0730(19)
b [Å]	24.413(5)	40.395(8)	23.5250(11)
c [Å]	34.288(7)	52.52(3)	31.7190(18)
α [Å]	73.77(3)	92.92(12)	90
β [Å]	82.35(3)	90.18(7)	90
γ [Å]	84.15(3)	90.65(3)	90
Volume [Å^3]	14774(6)	72510(62)	20202(2)
Z	2	16	4
ρ_{calc} [g/cm^3]	0.993	0.997	0.848
μ [mm^{-1}]	0.221	0.677	0.607
F(000)	4594	22594	5292
Crystal size [mm^3]	0.060×0.060×0.010	0.230×0.010×0.010	0.015×0.010×0.005
Crystal color	red	orange	orange
Crystal shape	plate	needle	block
Radiation	synchrotron (λ=0.6888 Å)	synchrotron (λ=1.0332 Å)	synchrotron (λ=1.0332 Å)
2Θ range [°]	1.21 to 34.24 (1.17 Å)	1.47 to 45.71 (1.33 Å)	3.33 to 42.98 (1.41 Å)
Index ranges	-15 ≤ h ≤ 15<br>-20 ≤ k ≤ 20<br>-29 ≤ l ≤ 29	-25 ≤ h ≤ 25<br>-30 ≤ k ≤ 30<br>-39 ≤ l ≤ 39	-19 ≤ h ≤ 19<br>-16 ≤ k ≤ 16<br>-22 ≤ l ≤ 22
Reflections collected	64206	215628	24451
Independent reflections	18536<br>R_{int} = 0.0437<br>R_{sigma} = 0.0407	58114<br>R_{int} = 0.1262<br>R_{sigma} = 0.0977	3758<br>R_{int} = 0.0915<br>R_{sigma} = 0.0517
Completeness to θ = 25.242°	96.0 %	90.0 %	99.5 %
Data / Restraints / Parameters	18536/5722/2893	58114/27429/13748	3758/1738/846
Goodness-of-fit on F^2	1.676	1.383	1.725
Final *R* indexes [$I \geq 2\sigma(I)$]	R_1 = 0.1367<br>wR_2 = 0.3786	R_1 = 0.1239<br>wR_2 = 0.3483	R_1 = 0.1404<br>wR_2 = 0.4070
Final *R* indexes [all data]	R_1 = 0.1647<br>wR_2 = 0.4041	R_1 = 0.1734<br>wR_2 = 0.3827	R_1 = 0.1821<br>wR_2 = 0.4388
Largest peak/hole [eÅ^3]	1.24/-0.84	0.78/-0.50	0.87/-0.40

Table S4: Crystal data and structure refinement for supramolecular assemblies

**Compound**	Pd_2(**L3**)$_4$	Pd_2(**L3**)$_4$
**CIF ID**	**ir13b_sq**	**ir9b sq**
**CCDC no**	**2168205**	**2168206**
Empirical formula	$C_{166}H_{177}B_3F_{12}N_{19}O_8Pd_2$	$C_{160}H_{168}B_3F_{12}N_{16}O_8Pd_2$
Formula weight	3039.49	2916.32
Temperature [K]	100(2)	80(2)
Crystal system	triclinic	triclinic
Space group (number)	$P\bar{1}$ (2)	$P\bar{1}$ (2)
a [Å]	19.3748(10)	19.108(4)
b [Å]	20.4931(10)	20.566(4)
c [Å]	41.073(2)	20.919(4)
α [Å]	88.174(3)	89.12(3)
β [Å]	83.403(3)	69.94(3)
γ [Å]	83.358(3)	83.03(3)
Volume [Å^3]	16088.3(14)	7662(3)
Z	4	2
ρ_{calc} [g/cm^3]	1.255	1.264
μ [mm^{-1}]	2.418	0.282
F(000)	6340	3038
Crystal size [mm^3]	0.150×0.100×0.050	0.200×0.200×0.005
Crystal color	red	red
Crystal shape	block	plate
Radiation	CuK_α (λ=1.54178 Å)	synchrotron (λ=0.6888 Å)
2Θ range [°]	4.33 to 133.19 (0.84 Å)	1.93 to 37.55 (1.07 Å)
Index ranges	-20 ≤ h ≤ 23<br>-24 ≤ k ≤ 24<br>-48 ≤ l ≤ 48	-17 ≤ h ≤ 17<br>-19 ≤ k ≤ 19<br>-19 ≤ l ≤ 19
Reflections collected	323162	42556
Independent reflections	55587<br>R_{int} = 0.1341<br>R_{sigma} = 0.0884	12452<br>R_{int} = 0.0595<br>R_{sigma} = 0.0600
Completeness to<br>θ = 25.242°	97.8 %	95.0 %
Data / Restraints / Parameters	55587/9566/4468	12452/4579/2102
Goodness-of-fit on F^2	1.344	1.581
Final *R* indexes<br>[I≥2σ(I)]	R_1 = 0.1268<br>wR_2 = 0.3377	R_1 = 0.1304<br>wR_2 = 0.3654
Final *R* indexes<br>[all data]	R_1 = 0.1738<br>wR_2 = 0.3725	R_1 = 0.1668<br>wR_2 = 0.4037
Largest peak/hole [eÅ^3]	4.21/-1.30	0.87/-0.61

Crystal structure of ring $Pd_3(L4)_6$ (ir12a)

Colorless, needle-shaped crystals of Pd_3(**L4**)$_6$ were grown by slow vapor diffusion of diisopropylether into a solution of Pd_3(**L4**)$_6$ in acetonitrile at 4°C. A single crystal in mother liquor was pipetted onto a glass slide containing NVH oil. To avoid collapse of the crystal lattice, the crystal was quickly mounted onto a 0.2 mm nylon loop and immediately flash-cooled in liquid nitrogen. Crystals were stored at cryogenic temperature in dry shippers, in which they were safely transported to macromolecular beamline P11 at Petra III,[7] DESY, Hamburg, Germany. A wavelength of λ = 0.6888 Å was chosen using a liquid N_2 cooled double crystal monochromator. Single crystal X-ray diffraction data was collected at 80(2) K on a single axis goniometer, equipped with an Oxford Cryostream 800 and a Pilatus 6M detector. 3600 diffraction images were collected in a 360° φ sweep at a detector distance of 200 mm, 100% filter transmission, 0.1° step width and 0.1 seconds exposure time per image. Data integration and reduction were undertaken using XDS.[8] The structure was solved by intrinsic phasing/direct methods using SHELXT[9] and refined with SHELXL[10] using 22 CPU cores for full-matrix least-squares routines on F^2 and ShelXle[11] as a graphical user interface and the DSR program plugin was employed for modeling.[12,13] Stereochemical restraints for the **L4** ligands (residue MMF) were generated by the GRADE program using the GRADE Web Server (http://grade.globalphasing.org) and applied in the refinement. A GRADE dictionary for SHELXL contains target values and standard deviations for 1,2-distances (DFIX) and 1,3-distances (DANG), as well as restraints for planar groups (FLAT). All displacements for non-hydrogen atoms were refined anisotropically. The refinement of ADP's for carbon, nitrogen and oxygen atoms was enabled by a combination of similarity restraints (SIMU) and rigid bond restraints (RIGU).[14] The contribution of the electron density from disordered counterions and solvent molecules, which could not be modeled with discrete atomic positions were handled using the SQUEEZE[15] routine in PLATON.[16] The solvent mask file (.fab), computed by PLATON, was included in the SHELXL refinement via the ABIN instruction leaving the measured intensities untouched.

Specific refinement details of $Pd_3(L4)_6$ ring (ir12a)

Besids the supramolecular ring, five out of six tetrafluoroborate counterions were modelled. One tetrafluoroborate counterion and two of the hexyl side chains (residue 8 and 9) were disordered and hence modelled with two conformations using a free variable for the occupancy factors.

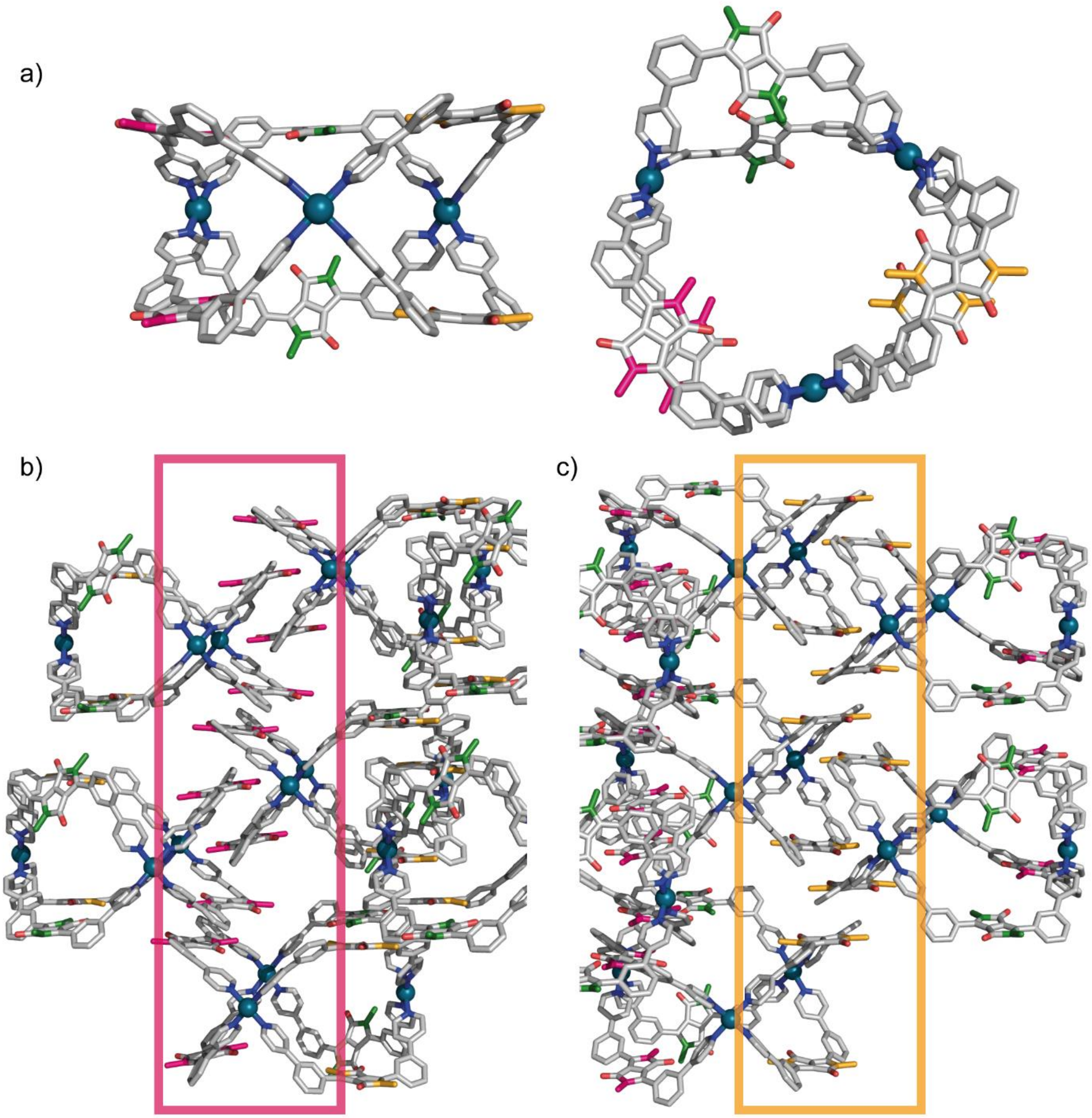

Figure S53: a) Side and top views of the crystal structure of ring $Pd_3(L4)_6$. The hexyl chains have been omitted for clarity. b) and c) Exemplary packing arrangements.

Crystal structure of heteroleptic $Pd_2(LQ)_2(LC)_2$ (ir36_needle)

Orange needle-shaped crystals of $Pd_2(\mathbf{LQ})_2(\mathbf{LC})_2$ were grown by slow vapor diffusion of methyl,*t*-butylether into the solution of $Pd_2(\mathbf{LQ})_2(\mathbf{LC})_2$ in acetonitrile at room temperature. Single crystals in mother liquor were pipetted onto a glass slide containing NVH oil. To avoid collapse of the crystal lattice, the crystal was quickly mounted onto a 0.2 mm nylon loop and immediately flash-cooled in liquid nitrogen. Crystals were stored at cryogenic temperature in dry shippers, in which they were safely transported to macromolecular beamline P11 at Petra III[17], DESY, Hamburg, Germany. A wavelength of λ = 1.0332 Å was chosen using a liquid N_2 cooled double crystal monochromator. Single crystal X-ray diffraction data was collected at 100(2) K on a single axis goniometer, equipped with an Oxford Cryostream 800 and an Eiger 2x 16M detector. 3600 diffraction images were collected in a 360° φ sweep at a detector distance of 154 mm, 100% filter

transmission, 0.1° step width and 0.1 seconds exposure time per image. Data integration and reduction were undertaken using XDS.[8] The structure was solved by intrinsic phasing/direct methods using SHELXT[9] and refined with SHELXL[10] using 22 CPU cores for full-matrix least-squares routines on F^2 and ShelXle[11] as a graphical user interface and the DSR program plugin was employed for modeling.[12,13]

Specific refinement details for $Pd_2(LQ)_2(LC)_2$ (ir36_needle)

Stereochemical restraints for the **LQ** (residue QPD) and **LC** (residue COP) ligands were generated by the GRADE program using the GRADE Web Server (http://grade.globalphasing.org) and applied in the refinement. A GRADE dictionary for SHELXL contains target values and standard deviations for 1,2-distances (DFIX) and 1,3-distances (DANG), as well as restraints for planar groups (FLAT). Additionally, non-crystallographic symmetry restraints (NCS) were applied to the highly flexible alkyl chains. All displacements for non-hydrogen atoms were refined anisotropically. The refinement of ADP's for carbon, nitrogen and oxygen atoms was enabled by a combination of similarity restraints (SIMU) and rigid bond restraints (RIGU).[14] The contribution of the electron density from disordered counterions and solvent molecules, which could not be modeled with discrete atomic positions, were handled using the SQUEEZE[15] routine in PLATON.[16] The solvent mask file (.fab) computed by PLATON was included in the SHELXL refinement via the ABIN instruction leaving the measured intensities untouched.

The compound has crystallized in triclinic space group P-1 with 8 heteroleptic assemblies and respective counterions in the asymmetric unit. The crystallographic analysis was hampered by the large number of atoms to be modelled, but especially the highly flexible alkyl chains, which have a strong tendency to disorder. For improved solubility, the ligands **LQ** with the DPP backbone (residue QPD) each have two hexyl chains attached, the ligands **LC** with the carbazole backbone (residue COP) each have one octyl chain attached. In all eight cages in the asymmetric unit, this sums up to 32 hexyl chains and 16 octyl chains, which we modelled in the best possible way. Some of the terminal atoms of the alkyl chains appeared to be disordered. However, we were unable to model this correlated disorder of several neighboring chains at the experimental resolution reached (1.3 A). Instead, we only modelled the alkyl chains as far as supported by the F_o map and as long as no symmetry clashes of neighboring chains or backbones were observed. This results in a reduced chain length for several of the ligands and is comparable to leaving out highly flexible loop regions of proteins in the modelling process in case no clear electron density can be observed experimentally. We have detailed the unmodelled atoms of the alkyl chains in the following table. While the unmodelled portion of the structure seems quite substantial when looking at this table, it should be emphasized, that 264 out of 320 alkyl carbon atoms (82.8%) have been successfully modelled and the chemically most interesting core of the cage structure is clearly structurally characterized. In the assembly's cavity, there are two small-volume binding sites close to each of the Pd(II) cations. These appear to be occupied by a water molecule as the scattering factor of oxygen is fitting well. The positions of hydrogen atoms for all of the 16 encapsulated water molecules in the entire structure were refined with the help of 1.2 and 1.3 bond restraints using DFIX and DANG from a GRADE dictionary for water. Additionally, distance restraints to the acceptor oxygen atoms were used. Only for one out of sixteen water sites (residue numbers 52 and 53) the refinement of hydrogen atoms did not converge, so that coordinates of both H1 and H2 in this residue were fixed after initial refinement with with conjugate gradients (CLGS) for the final refinement using full matrix least squares (LS) by appying AFIX 1 command.

Table S5: Unmodelled atoms of alkyl chains for $Pd_2(LQ)_2(LC)_2$ (ir36_needle)

Residue number	Residue name	Original alkyl chain length	Modelled alkyl chain	Unmodeled atoms
2	COP	C_8	C_6	C17G, C17H
3	COP	C_8	C_4	C17E, C17F, C17G, C17H
4	QPD	C_6	C_5	C24
7	COP	C_8	C_5	C17F, C17G, C17H
8	COP	C_8	C_6	C17G, C17H
10	QPD	C_6<br>C_6	C_3<br>C_5	C16, C17, C18<br>C24
12	COP	C_8	C_6	C17G, C17H
13	COP	C_8	C_6	C17H

14	QPD	C_6	C_3	C16, C17, C18
15	QPD	C_6<br>C_6	C_5<br>C_3	C18<br>C22, C23, C24
18	COP	C_8	C_5	C17F, C17G, C17H
19	QPD	C_6	C_5	C18
20	QPD	C_6<br>C_6	C_5<br>C_4	C18<br>C23, C24
22	COP	C_8	C_4	C17E, C17F, C17G, C17H
23	COP	C_8	C_8	---
24	QPD	C_6<br>C_6	C_5<br>C_5	C18<br>C24
25	QPD	C_6	C_5	C18
27	COP	C_8	C_7	C17H
28	COP	C_8	C_7	C17H
32	COP	C_8	C_6	C17G, C17H
33	COP	C_8	C_6	C17G, C17H
34	QPD	C_6	C_5	C18
35	QPD	C_6	C_5	C24
37	COP	C_8	C_6	C17G, C17H
38	COP	C_8	C_5	C17F, C17G, C17H
39	QPD	C_6	C_5	C18
40	QPD	C_6	C_4	C23, C24

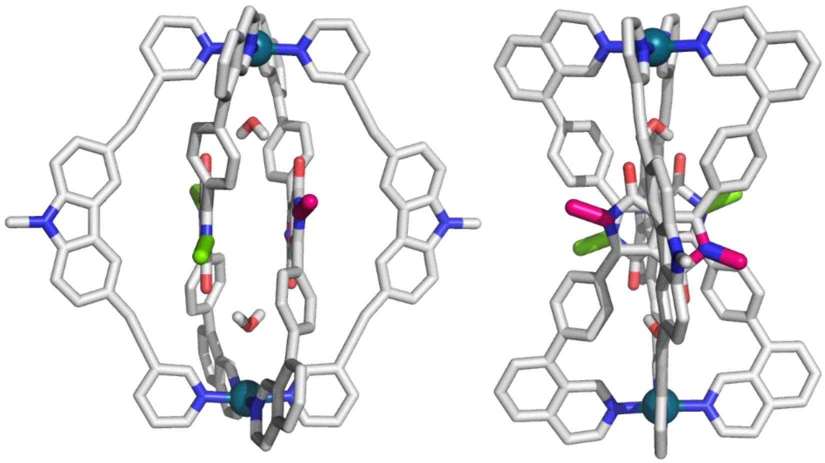

Figure S54: Two side views of one of the eight assemblies with figure-eight topology in the asymmetric unit of the X-ray crystal structure of $Pd_2(LQ)_2(LC)_2$. The alkyl chains have been omitted for clarity.

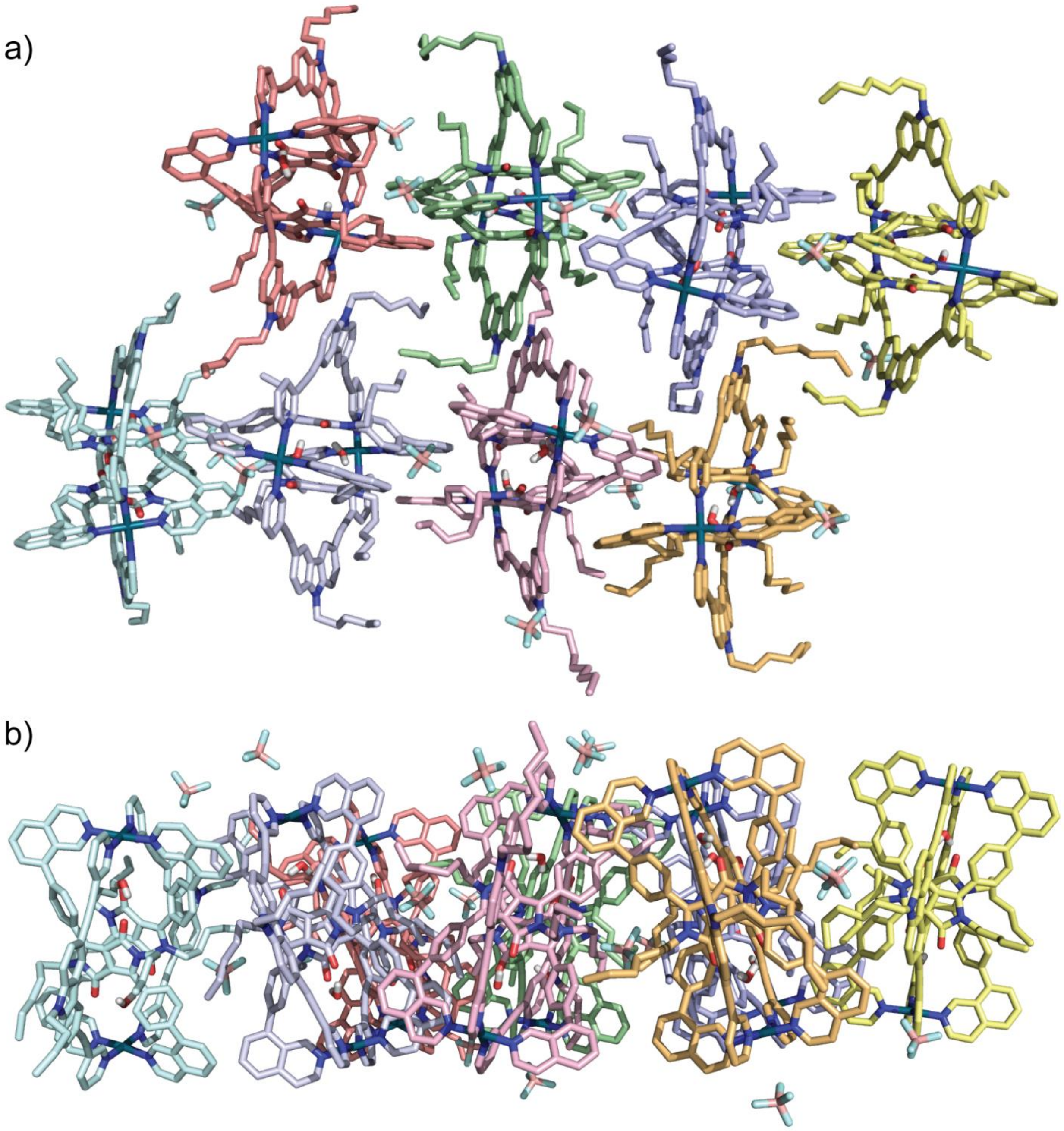

Figure S53: a) Top and b) side view of the asymmetric unit for $Pd_2(LQ)_2(LC)_2$ (ir36_needle). The 8 different assemblies are represented in different colors to facilitate the comprehension of their organization in space.

Crystal structure of $Pd_2(LQ)_2(LC)_2$ (ir36_block)

Orange block-shaped crystals of Pd_2(**LQ**)$_2$(**LC**)$_2$ were grown from exactly the same conditions and in the same vial as the needle-shaped crystals (ir36_needle), by slow vapor diffusion of methyl,*t*-butylether into the solution of Pd_2(**LQ**)$_2$(**LC**)$_2$ in acetonitrile at room temperature. Single crystals in mother liquor were pipetted onto a glass slide containing NVH oil. To avoid collapse of the crystal lattice, the crystal was quickly mounted onto a 0.2 mm nylon loop and immediately flash-cooled in liquid nitrogen. Crystals were stored at cryogenic temperature in dry shippers, in which they were safely transported to macromolecular beamline

P11 at Petra III[7], DESY, Hamburg, Germany. A wavelength of λ = 1.0332 Å was chosen using a liquid N_2 cooled double crystal monochromator. Single crystal X-ray diffraction data was collected at 100(2) K on a single axis goniometer, equipped with an Oxford Cryostream 800 and an Eiger 2x 16M detector. 1800 diffraction images were collected in a 180° φ sweep at a detector distance of 154 mm, 100% filter transmission, 0.1° step width and 0.15 seconds exposure time per image. Data integration and reduction were undertaken using XDS.[8] The structure was solved by intrinsic phasing/direct methods using SHELXT[9] and refined with SHELXL[10] using 22 CPU cores for full-matrix least-squares routines on F^2 and ShelXle [11] as a graphical user interface and the DSR program plugin was employed for modeling.[12,13]

Specific refinement details for $Pd_2(LQ)_2(LC)_2$ (ir36_block)

Stereochemical restraints for the **LQ** (residue QPD) and **LC** (residue COP) ligands were generated by the GRADE program using the GRADE Web Server (http://grade.globalphasing.org) and applied in the refinement. A GRADE dictionary for SHELXL contains target values and standard deviations for 1,2-distances (DFIX) and 1,3-distances (DANG), as well as restraints for planar groups (FLAT). All displacements for non-hydrogen atoms were refined anisotropically. The refinement of ADP's for carbon, nitrogen and oxygen atoms was enabled by a combination of similarity restraints (SIMU) and rigid bond restraints (RIGU).[14] The contribution of the electron density from disordered counterions and solvent molecules, which could not be modeled with discrete atomic positions were handled using the SQUEEZE[15] routine in PLATON.[16] The solvent mask file (.fab) computed by PLATON was included in the SHELXL refinement via the ABIN instruction leaving the measured intensities untouched.

The tiny block-shaped crystalized in orthorhombic space group $P\mathrm{bcn}$ (60) and only contains half a cage in the asymmetric unit, so that the structure modelling was by far less complex compared to the gigantic ir36_needle structure. However, also in this case not all side chains could be refined. Ligand **LQ** with DPP backbone (residue QPD) has two hexyl chains attached, one was modelled as propyl the other as butyl chain. Ligand **LC** with carbazole backbone (residue COP) has one octyl chain attached, which could only be modelled as propyl chain. In total only 10 out of 20 alkyl carbon atoms (50%) have been successfully modelled. In the assembly's cavity, there are two small-volume binding sites close to each of the Pd(II) cations. These appear to be occupied by a water molecule as the scattering factor of oxygen is fitting well. Positions of hydrogen atoms of both encapsulated water molecules were refined with the help of 1.2 and 1.3 bond restraints using DFIX and DANG from a GRADE dictionary for water. Due to the special position (twofold axis) restraints from GRADE restraint dictionary were adapted to the symmetry equivalent position of the hydrogen atom using EQIV command.

Crystal structure of $Pd_2(L3)_4$ (ir13b)

Colorless, needle-shaped crystals of Pd_2(**L3**)$_4$ were grown by slow vapor diffusion of diethyl ether into solution of Pd_2(**L3**)$_4$ in acetonitrile at room temperature. A single crystal in mother liquor was pipetted onto a glass slide containing NVH oil. To avoid collapse of the crystal lattice, the crystal was quickly mounted onto a 0.2 mm nylon loop and immediately flash-cooled in liquid nitrogen. Diffraction data were collected at 100(2) K on a Bruker D8 Venture with INCOATEC microfocus sealed tube, Iμs 3.0 using multilayer optics as monochromator and a Photon 2 detector. The diffractometer was equipped with an Oxford Cryostream 800 low temperature device and used CuK_α radiation (λ = 1.54178 Å). In total 21 sweeps with a detector distance of 45 mm and exposure times from 4s/° for low order (2Θ = 0°) up to 140s/° for high order (2Θ = 104°) were collected with an increment of 0.5° per image. All data were integrated with SAINT and a multi-scan absorption correction using SADABS was applied. The structure was solved by intrinsic phasing/direct methods using SHELXT[9] and refined with SHELXL[10] using 22 CPU cores for full-matrix least-squares routines on F^2 and ShelXle [11] as a graphical user interface and the DSR program plugin was employed for modeling.[12,13] Stereochemical restraints for the **L3** ligands (residue type DPP) were generated by the GRADE program using the GRADE Web Server (http://grade.globalphasing.org) and applied in the refinement. A GRADE dictionary for SHELXL contains target values and standard deviations for 1,2-distances (DFIX) and 1,3-distances (DANG), as well as restraints for planar groups (FLAT). All displacements for non-hydrogen atoms were refined anisotropically. The refinement of ADP's for carbon, nitrogen and oxygen atoms was enabled by a combination of similarity restraints (SIMU) and rigid bond restraints (RIGU).[14] The contribution of the electron density from disordered counterions and solvent

molecules, which could not be modeled with discrete atomic positions were handled using the SQUEEZE[15] routine in PLATON.[16] The solvent mask file (.fab) computed by PLATON was included in the SHELXL refinement via the ABIN instruction leaving the measured intensities untouched.

Crystal structure of Pd_2(L3)$_4$ (ir9b)

Red, plate-shaped crystals of Pd_3(**L4**)$_6$ were grown by slow vapor diffusion of methyl,*t*-buthyl ether into a solution of Pd_2(**L3**)$_4$ in acetonitrile at room temperature . A single crystal in mother liquor was pipetted onto a glass slide containing NVH oil. To avoid collapse of the crystal lattice, the crystal was quickly mounted onto a 0.2 mm nylon loop and immediately flash-cooled in liquid nitrogen. Crystals were stored at cryogenic temperature in dry shippers, in which they were safely transported to macromolecular beamline P11 at Petra III[7], DESY, Hamburg, Germany. A wavelength of λ = 0.6888 Å was chosen using a liquid N_2 cooled double crystal monochromator. Single crystal X-ray diffraction data was collected at 80(2) K on a single axis goniometer, equipped with an Oxford Cryostream 800 and a Pilatus 6M detector. 3600 diffraction images were collected in a 360° φ sweep at a detector distance of 200 mm, 100% filter transmission, 0.1° step width and 0.15 seconds exposure time per image. Data integration and reduction were undertaken using XDS.[8] The structure was solved by intrinsic phasing/direct methods using SHELXT[9] and refined with SHELXL[10] using 22 CPU cores for full-matrix least-squares routines on F^2 and ShelXle[11] as a graphical user interface and the DSR program plugin was employed for modeling.[12,13] Stereochemical restraints for the **L3** ligands were generated by the GRADE program using the GRADE Web Server (http://grade.globalphasing.org) and applied in the refinement. A GRADE dictionary for SHELXL contains target values and standard deviations for 1,2-distances (DFIX) and 1,3-distances (DANG), as well as restraints for planar groups (FLAT). All displacements for non-hydrogen atoms were refined anisotropically. The refinement of ADP's for carbon, nitrogen and oxygen atoms was enabled by a combination of similarity restraints (SIMU) and rigid bond restraints (RIGU).[14] The contribution of the electron density from disordered counterions and solvent molecules, which could not be modeled with discrete atomic positions were handled using the SQUEEZE[15] routine in PLATON.[16] The solvent mask file (.fab) computed by PLATON was included in the SHELXL refinement via the ABIN instruction leaving the measured intensities untouched.

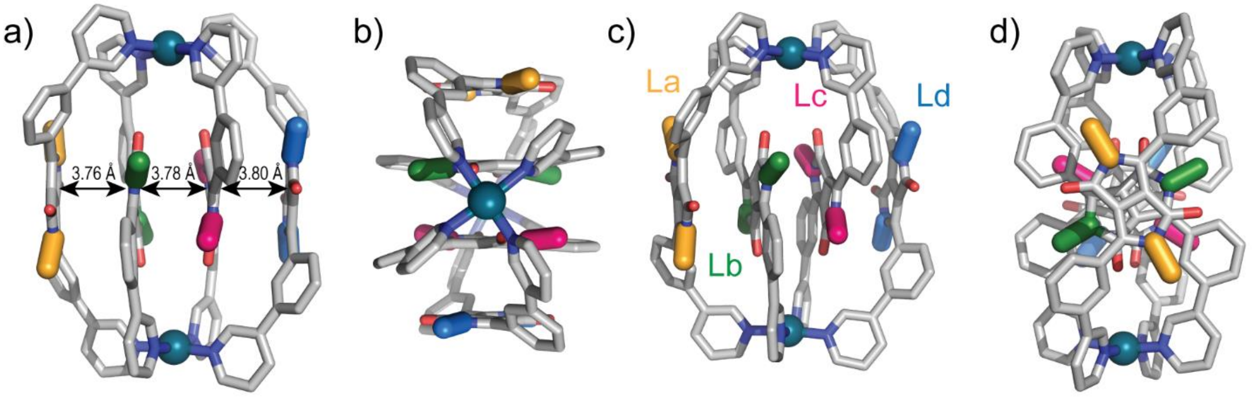

Figure S54: Crystal structure of Pd_2(L3)$_4$. a) Side view with indicated distances between the stacked chromophore units b) top view, c) and d) further side views. The C-N bond of different dye backbones are differently colored and labelled. The different colors serve the purpose of better highlighting the alternating rotation of the dyes within the stack.

Table S6: Angles between the vectors defined by the N atoms of each DPP moiety (pictured in different colors in Figure S53). The angles in bold along the diagonal of the tables refer to the orientation of two neighboring DPP units.

	Lb	Lc	Ld
La	**89.60°**	33.55°	63.81°
Lb	/	**57.32°**	27.07°
Lc	/	/	**84.39°**

5. Supplemental References

[1] Spartan`18 Parallel Suite, Wavefunction, Inc., Irvine.

[2] Gaussian 16, Revision C.01, Frisch, M. J. et. al Gaussian, Inc., Wallingford CT, 2016.

[3] R. Zhu, J. Lübben, B. Dittrich, G. H. Clever, *Angew. Chem. Int. Ed.* **2015**, 54, 2796.

[4] W. M. Bloch, J. J. Holstein, W. Hiller, G. H. Clever, *Angew. Chem. Int. Ed.* **2017**, *56*, 8285.

[5] J. C. de Mello, H. F. Wittmann, R. H. Friend, *Adv. Mater.* **1997**, *9*, 230.

[6] N. C. Greenham, I. D. W. Samuel, G. R. Hayes, R. T. Phillips, Y. A. R. R. Kessener, S. C. Moratti, A. B. Holmes, R. H. Friend, *Chem. Phys. Lett.* **1995**, *241*, 89.

[7] A. Burkhardt, T. Pakendorf, B. Reime, J. Meyer, P. Fischer, N. Stübe, S. Panneerselvam, O. Lorbeer, K. Stachnik, M. Warmer, P. Rödig, D. Göries, A. Meents, *Eur. Phys. J. Plus.* **2016**, *131*, 56.

[8] W. Kabsch, *Acta Crystallogr. Sect. D* **2010**, *66*, 133–144.

[9] G. M. Sheldrick, *Acta Crystallogr. Sect. A* **2015**, *71*, 3.

[10] G. M. Sheldrick, *Acta Crystallogr. Sect. C* **2015**, *71*, 3.

[11] C. B. Hubschle, G. M. Sheldrick, B. Dittrich, *J. Appl. Crystallogr.* **2011**, *44*, 1281.

[12] D. Kratzert, J. J. Holstein, I. Krossing, *J. Appl. Crystallogr.* **2015**, *48*, 933.

[13] Kratzert, D., and Krossing, I. (2018). Recent improvements in DSR. J. Appl. Crystallogr. *51*, 928–934.

[14] A. Thorn, B. Dittrich, G. M. Sheldrick, *Acta Crystallogr. Sect. A* **2012**, *68*, 448.

[15] A. Spek, *Acta Crystallogr. Sect. C* **2015**, *71*, 9.

[16] A. Spek, *Acta Crystallogr. Sect. D* **2009**, *65*, 148.
